# Dynamics of the Energy Transfer Process in Eu(III) Complexes Containing Polydentate Ligands Based on Pyridine, Quinoline, and Isoquinoline as Chromophoric Antennae

**DOI:** 10.1021/acs.inorgchem.2c02330

**Published:** 2022-10-06

**Authors:** Albano N. Carneiro Neto, Renaldo T. Moura, Luís D. Carlos, Oscar L. Malta, Martina Sanadar, Andrea Melchior, Elfi Kraka, Silvia Ruggieri, Marco Bettinelli, Fabio Piccinelli

**Affiliations:** † Physics Department and CICECO-Aveiro Institute of Materials, University of Aveiro, 3810-193Aveiro, Portugal; ‡ Department of Chemistry and Physics, Federal University of Paraíba, 58397-000Areia, Brazil; § Department of Chemistry, Southern Methodist University, Dallas, Texas75275-0314, United States; ∥ Department of Fundamental Chemistry, Federal University of Pernambuco, 50740-560Recife, Brazil; ⊥ Dipartimento Politecnico di Ingegneria e Architettura, Laboratorio di Tecnologie Chimiche, University of Udine, 33100Udine, Italy; # Luminescent Materials Laboratory, Department of Biotechnology, University of Verona and INSTM, UdR Verona, 37134Verona, Italy

## Abstract

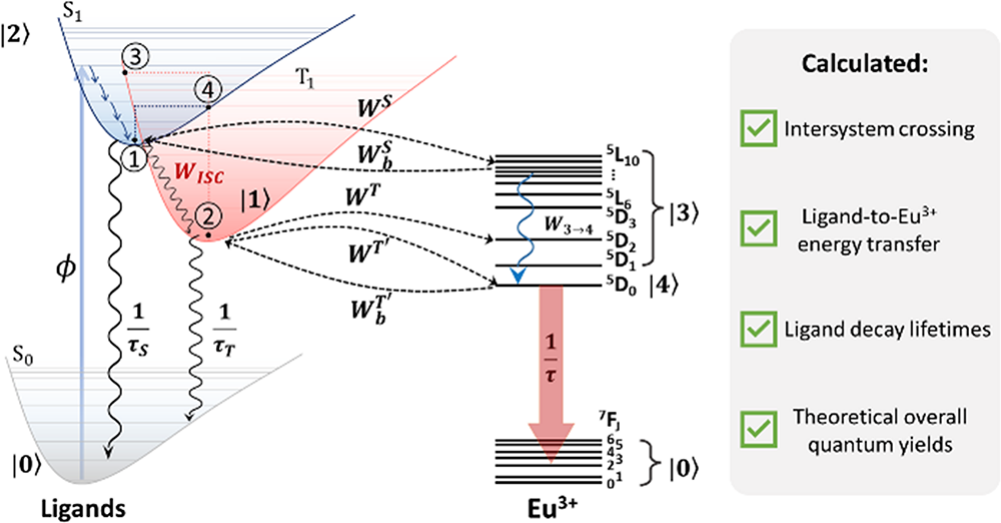

In this work, we investigated from a theoretical point of view the dynamics of the energy transfer process from the ligand to Eu(III) ion for 12 isomeric species originating from six different complexes differing by nature of the ligand and the total charge. The cationic complexes present the general formula [Eu(L)(H_2_O)_2_]^+^ (where L = bpcd^2–^ = *N*,*N*′-bis(2-pyridylmethyl)-*trans*-1,2-diaminocyclohexane *N*,*N*′-diacetate; bQcd^2–^ = *N*,*N*′-bis(2-quinolinmethyl)-*trans*-1,2-diaminocyclohexane *N*,*N*′-diacetate; and b*iso*Qcd^2–^ = *N*,*N*′-bis(2-isoquinolinmethyl)-*trans-*1,2-diaminocyclohexane *N*,*N*′-diacetate), while the neutral complexes present the Eu(L)(H_2_O)_2_ formula (where L = PyC3A^3–^ = *N*-picolyl-*N*,*N*′,*N*′-*trans*-1,2-cyclohexylenediaminetriacetate; QC3A^3–^ = *N*-quinolyl-*N*,*N*′,*N*′-*trans-*1,2-cyclohexylenediaminetriacetate; and *iso*QC3A^3–^ = *N*-isoquinolyl-*N*,*N*′,*N*′-*trans-*1,2-cyclohexylenediaminetriacetate). Time-dependent density functional theory (TD-DFT) calculations provided the energy of the ligand excited donor states, distances between donor and acceptor orbitals involved in the energy transfer mechanism (*R*_L_), spin-orbit coupling matrix elements, and excited-state reorganization energies. The intramolecular energy transfer (IET) rates for both singlet-triplet intersystem crossing and ligand-to-metal (and vice versa) involving a multitude of ligand and Eu(III) levels and the theoretical overall quantum yields (ϕ_ovl_) were calculated (the latter for the first time without the introduction of experimental parameters). This was achieved using a blend of DFT, Judd–Ofelt theory, IET theory, and rate equation modeling. Thanks to this study, for each isomeric species, the most efficient IET process feeding the Eu(III) excited state, its related physical mechanism (exchange interaction), and the reasons for a better or worse overall energy transfer efficiency (η_sens_) in the different complexes were determined. The spectroscopically measured ϕ_ovl_ values are in good agreement with the ones obtained theoretically in this work.

## Abstract 2

Photophysical properties of 12 Eu(III) complexes with pyridine, quinoline, and isoquinoline ligands in aqueous solutions were elucidated and predicted through a theoretical protocol using a blend of DFT, Judd−Ofelt theory, intramolecular energy transfer theory, and coupled rate equation modeling calculations. The theoretical procedure is general and can be extended to any lanthanide-based complexes.

## Introduction

The nonradiative intramolecular energy transfer (IET) process (also called *antenna effect*) is broadly exploited to increase the brightness of luminescent metal complexes, in particular for trivalent lanthanide ions^1^ when, for example, these molecules are employed as optical probes for imaging^1−4^ and luminescent sensing.^5−14^ For such a class of compounds, brightness is defined as *B* = ε · ϕ_ovl_ = ε · η_sens_ · ϕ_int_, where ε is the molar absorption coefficient, ϕ_ovl_ is the overall quantum yield, i.e., the ratio of emitting/absorbed photons by the matrix/ligand, η_sens_ is the overall energy transfer efficiency, and ϕ_int_ is the intrinsic quantum yield, i.e., the ratio of emitting/absorbed photons upon direct excitation into a luminescent level of the lanthanide ion. Thus, to obtain high values of brightness, the combination of high ε, η_sens_, and ϕ_int_ values is required (η_sens_ = η_ISC_ · η_IET_; where η_ISC_ and η_IET_ are the efficiency of the intersystem crossing (ISC)^15^ and the IET processes, respectively). In this direction, one should avoid nonradiative processes, which negatively impact the ϕ_int_ value. These processes consist of (i) multiphonon relaxation (MPR) and (ii) backward energy transfer. They both may induce the nonradiative relaxation of the emitting levels, and the MPR process is particularly effective when high-energy vibrational modes (e.g., C–H, N–H, and O–H stretching) couple with the lanthanide electronic levels.^16−18^ To obtain high ε and η_sens_ values, chromophoric ligands are employed, since they are capable, at the same time, of strong absorption of light (high ε value, usually in the UV spectral range) and efficiently transfer the excitation energy to the lanthanide ion (high η_sens_ values).

From Dexter’s theory,^19^ nonradiative energy transfer processes from a donor (D, in the present case the ligand) to an acceptor (A, the lanthanide ion) can occur mainly by electric dipole–dipole, electric dipole–quadrupole, and exchange interactions. To optimize the efficiency of this process, the following requirements must be satisfied: (i) selection rules in the transitions of both D and A, (ii) short D–A distance (*R*_L_), and (iii) strong spectral overlap of the emission band of D and the absorption band of A. In order to control all these features, precise knowledge of the D–A interaction is required. A complete and detailed description of the mechanism based on this interaction can be obtained by means of a combined experimental and theoretical study.^20^ The photon absorption by D leads to an electron excitation that can result in S_0_ → S_1_ or S_0_ → T_1_ transitions. The S_1_ state may nonradiatively transfer energy to a triplet excited state T_1_ (a process known as intersystem crossing—ISC) or transfer energy to A (lanthanide ion), while the S_0_ → T_1_ excitation involves a spin-forbidden transition and is much less probable that a triplet state will form. Ln(III)-based complexes favor ISC as a result of their more intense spin-orbit coupling (SOC),^21^ which grows with the fourth power of the atomic number *Z*. Consequently, the theoretical methodology applied to account the D–A interaction requires the proper treatment of relativistic effects, which can be included by different methodologies, namely, the zero-order regular approximation (ZORA),^22,23^ the Douglas–Kroll–Hess (DKH)^24,25^ Hamiltonian, or the normalized elimination of the small component (NESC) method^26^ and its updates and new implementations.^27−31^ These methods offer different strengths and computational costs and are scalar relativistic corrections for the contraction of s- or p-orbitals and the expansion of d- or f-orbitals.

The S_0_ → T_1_ and S_1_ → T_1_ ISC at the scalar relativistic level is spin-forbidden, but SOC induces fast ISC by mixing singlet and triplet states, enabling these processes. Thus, accurate theoretical methods that can treat excited states with SOC in the presence of heavy elements are necessary for a precise description of the D–A interaction.^32^ While different works in literature^33^ approach the ISC for organic and d-metal complexes, few works^34^ apply this type of calculation in lanthanide spectroscopy. However, the direct utilization of ISC for S_1_ → T_1_ and T_1_ → S_0_ nonradiative energy transfer rates on the calculation of the theoretical overall quantum yield (ϕ_ovl_), as far as we know, is not found in the literature for a big set of Eu(III) excited states.

For this reason, in this paper, we present a detailed theoretical investigation, employing a blend of density functional theory (DFT), Judd–Ofelt theory, IET theory, and rate equation modeling, aimed to determine the relevant photophysical properties for 12 isomeric species originating from six different complexes previously designed and synthesized. These results, combined with an experimental spectroscopic study, mainly carried out previously by some of us,^10,12,35^ give a detailed picture of the energy transfer mechanism in the different compounds. In particular, for Eu(III) complexes, we have determined (i) the nature and energy of the ligand levels (S_1_ and T_1_ donors) from DFT calculations; (ii) the D–A distances; (iii) the SOC matrix elements and reorganization energies for S_1_ → T_1_ (λ_M_) to calculate ISC rates (*W*_ISC_); (iv) the reliable S_0_ → S_1_ and S_0_ → T_1_ (via SOC) dipole strengths to calculate S_0_ and T_1_ lifetimes; and (v) the contribution of dipole–dipole, dipole–quadrupole, and exchange mechanisms to the energy transfer process.

For the first time, the five points described above are used to perform simulations of the emitting-level populations from an appropriate rate equation model. Furthermore, these simulations permit the quantification of theoretical overall quantum yield (ϕ_ovl_) without the introduction of experimental parameters. Thanks to this detailed study, we have been able to determine for each complex the main ligand and Eu(III) levels involved in the IET processes, the related physical mechanism, and the reasons for the better or worse overall energy transfer efficiency observed.

The deep knowledge of these photophysical properties is crucial in order to properly employ these classes of Eu(III) complexes as optical probes for the detection of important bioanalytes (i.e., citrate,^10^ serum proteins,^11^ hydrogen carbonate,^12^ and lactate^13^).

## Theoretical and Computational Procedures

### Computational Procedures

All molecular structures of the complexes were obtained by means of DFT calculations run in Gaussian 16 (version A.03).^36^ The paramagnetic Eu(III) ion has been replaced by Y(III), which is a suitable substitute as shown in a previous work^13^ and as can be deduced from the isostructural complexes with the analogous hexa-dentate ligands ethylenediaminetetraacetic acid (EDTA) and cyclohexanediaminetetraacetic acid (CDTA). In the crystal structures with these ligands, Y(III) and Eu(III) ions are nine-coordinated with EDTA (three water molecules bound) and eight-coordinated with CDTA (two bound waters).^37−39^

The functional B3LYP^40,41^ was used with the 6–31+G(d) basis set for all ligand atoms and MWB28 pseudo-potential and the valence electron basis set for the metal ion.^42^ Geometry optimizations were carried out at the DFT level with the solvent effect accounted by the polarizable continuum model (PCM) with the parameters for water.^43^

The excited states (T_1_ and S_1_) were obtained employing the time-dependent DFT approach (TD-DFT)^44^ using the same procedure described for the geometry optimizations (B3LYP/MWB28(Y)/6–31+G(d)/PCM).

*W*_ISC_ was calculated using the Marcus–Levich^45−47^ formalism. The values of λ_M_ were calculated utilizing the same procedure (B3LYP/MWB52(Eu)/6–31+G(d)/PCM) but selecting S_1_ and T_1_ excited states (from the TD-DFT calculations) for the geometry optimizations. The SOC matrix elements were calculated using SOC-TD-DFT^48^ in ORCA5.0,^49^ utilizing the B3LYP functional with ZORA^23^ relativistic corrections. The recontracted ZORA-DEF2-TZVP^50^ basis set for ligands and segmented all-electron relativistically contracted (SARC)-ZORA-TZVP for Eu atoms^51^ were utilized. The calculations were done with the SARC/J auxiliary basis^51,52^ and the resolution of the identity spin-orbit mean-field RI-SOMF^53^ approximation. The calculated values of λ_M_ and SOC matrix elements, as well as the *W*_ISC_ rates, can be found in Table S15. Taking advantage of the SOC-TD-DFT approach, the dipole strengths of S_0_ → T_1_ and S_0_ → S_1_ excitations were obtained for calculating the S_1_ and T_1_ decay lifetimes (τ_S_ and τ_T_, respectively) from Eq. S12.

### Intramolecular Energy Transfer

The IET rates from organic ligands to Eu(III) ion were estimated taking into consideration the dipole–dipole (*W*_d – d_), dipole–multipole (*W*_d – m_), and exchange (*W*_ex_) mechanisms:^20,54−57^
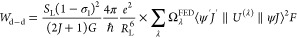
1
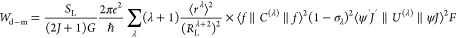
2
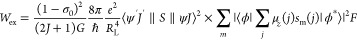
3where *R*_L_ is the donor–acceptor state distance and Ω_λ_^FED^ (ESI) are the intensity parameters (or Judd–Ofelt parameters) by the forced electric dipole (FED) mechanism.^58,59^ The simple overlap model^60,61^ was employed to calculate these quantities through the JOYSpectra web platform.^62^ The values of the squared reduced matrix elements ⟨ψ′*J*′∥*U*^(λ)^∥ψ*J*⟩^2^ were taken from Carnall et al.,^63^ whereas the ⟨ψ′*J*′∥*S*∥ψ*J*⟩ matrix elements were calculated using free-ion wavefunctions in the intermediate coupling scheme.^64,65^ The values of ⟨ψ′*J*′∥*S*∥ψ*J*⟩ for allowed (|Δ*J*| = 0 or 1) ^7^F_0_ → ^5^D_1_, ^7^F_1_ → ^5^D_0_, ^7^F_1_ → ^5^D_1_, ^7^F_1_ → ^5^D_2_, and ^7^F_1_ → ^5^G_2_ acceptor transitions are reported in Table S2 in the ESI. *S*_L_ is the dipole strength of the ligand transition involved in IET (10^–36^ and 10^–40^ (esu)^2^ · cm^2^ for S_1_ and T_1_, respectively^20^), ⟨*r*^λ^⟩ are the 4*f* radial integrals,^66^*G* is the ligand state degeneracy (*G* = 1 or 3 for S_1_ or T_1_, respectively), ⟨*f*∥*C*^(λ)^∥*f*⟩ is the reduced matrix element of Racah’s tensor operators,^67^ and (1 – σ_*k*_) (for *k* = 1 and 2) are the shielding factors, which have a relation with the overlap integrals between valence orbitals of the pair Ln–*X* (*X* as the ligating atom in the first coordination sphere).^57,68^ The values of (1 – σ_*k*_) for *k*= 4 and 6 can be found in ref (66). *s_m_* (in eq 3) is the spin operator in the ligand, and μ_*z*_ is the dipole operator (*z* component); the value of the element matrix of these coupled operators is 10^–36^ (esu)^2^ · cm^2^.^20,69^

The *F* term in eqs 1–3 is the density of states (proportional to the spectral overlap) that considers the energy mismatch condition between donor (ligands) and acceptor states (Ln(III) ion).^20,54^*F* can be estimated by
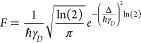
4where Δ is the energy difference between the donor barycenter state and the lanthanide ion acceptor state (Δ = *E_D_* – *E_A_*). γ*_D_* is the bandwidth at half-height of the donor states (S_1_ and T_1_), assumed here to have a typical value of γ*_D_* = 3000 cm^–1^ for both S_1_ and T_1_ states.^70,71^

The forward energy transfer rates (*W*) involving the Eu(III) ions as acceptors are calculated by the sum over eqs 1–3 in the same pathway:
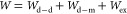
5If Δ is negative, for a given energy transfer pathway, *W* must be multiplied by the energy barrier factor exp(Δ/*k*_B_*T*), where *k*_B_ is the Boltzmann constant and *T* is the temperature (considered to be 298.15 K in the present work). For example, consider that a given forward pathway T_1_ → [^7^F_0_ → ^2S+1^L_*J*_] has Δ = −500 cm^–1^, then the total rate (eq 5) of this specific pathway should be multiplied by 0.09 (barrier factor). The barrier factor is not applied for the backward energy transfer [^2S+1^L_*J*_ → ^7^F_0_] → T_1_ because Δ = 500 cm^–1^. In addition, depending on the Eu(III) initial state involved in the energy transfer pathway (e.g., ^7^F_1_ in the T_1_ → [^7^F_1_ → ^5^D_1_] pathway or ^7^F_0_ in the S_1_ → [^7^F_0_ → ^5^L_6_] pathway), *W* is multiplied by the thermal population fraction at room temperature (0.64 for ^7^F_0_ and 0.33 for ^7^F_1_).^65,72^

The IET rates from the ligands to the Eu(III) ion were calculated using both S_0_ ← S_1_ and S_0_ ← T_1_ decay transitions as energy donors localized in the ligands and a total of 32 energy transfer pathways involving ^7^F_0_ and ^7^F_1_ as initial states and ^5^D_0_, ^5^D_1_, ^5^D_2_, ^5^D_3_, ^5^L_6_, ^5^L_7_, ^5^G_2_, ^5^G_3_, ^5^G_4_, ^5^G_6_, ^5^D_4_, ^5^G_5_, ^5^L_8_, ^5^D_4_, ^5^L_9_, and ^5^L_10_ as final states at the Eu(III) ion. Consequently, 64 IET pathways were computed for each one of the 12 studied complexes, totalizing 768 IET rates analyzed (half of them are non-zero due to selection rules on the *J* quantum number^20^).

### Rate Equations and Overall Emission Quantum Yield

Once the IET rates are determined, the system of rate equations constituted by coupled ordinary differential equations (ODEs) can be solved to determine the relative population of each level. The set of ODEs can be solved in the analytical form, which assumes that the system is in a steady-state regime (all derivatives equal to zero) and the ground levels are very little depleted. Thus, the population of the Ln(III) emitting level is given by analytical expressions.^20,65,73,74^ On the other hand, the set of coupled ODEs can be solved by numerical methods through time propagation. This approach was adopted in this work.

The set of ODEs can be described as follows:^14,20,75^
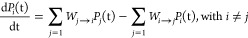
6where both summations run all levels of the system. *P_i_* and *P_j_* are the populations of the levels |*i*⟩ and |*j*⟩ and *W*_*j* → *i*_ and *W*_*i* → *j*_ are the energy transfer rates between these states. Thus, an *N*-level rate equation model can be described by a set formed by *N*-coupled ODEs. The appropriate set of rate equations, with their respective initial conditions, can be numerically solved using several methods such as fourth-order Runge–Kutta with fixed-step or adaptive-step size, Radau, and Adams–Bashforth, among others.^76^ We adopted the Radau method^77^ in the simulations since it was applied in other Ln-based complexes and provided very consistent results^14,78−83^ with a low computational cost. Each simulation was done in a time interval from 0 to 10 ms with a step-size of 2 μs.

The solution of the rate equation model permits the estimation of the emitting level population *P*_E_ of the Ln(III) (e.g., ^5^D_0_ of the Eu(III) ion), and consequently, the emission intensity *I* = *A*_rad_*P*_E_, where *A*_rad_ (Table S1) is the spontaneous emission coefficients, which can be calculated from the Judd–Ofelt intensity parameters.^20,58,59,62,63,84^

The overall quantum yield ϕ_ovl_ is defined by the ratio of the numbers of photons emitted and absorbed by the matrix/ligand,^1,20,85,86^
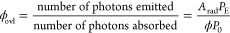
7where *P*_0_ is the population of the ground level and ϕ is the pumping rate of the populations from this level (e.g., S_0_ → S_1_ intra-ligand absorptions). The latter can be estimated by ϕ = σρλ_exc_/*hc*, where σ is the absorption cross section of the ligand (in the order of ∼10^–16^ cm^2^·molecule^−1^),^87^ ρ is the power density in units of watts per square centimeter, λ_exc_ is the excitation wavelength, *h* is Planck’s constant, and *c* is the speed of light.^14,78,79,88^

## Experimental Measurements

The overall quantum yields ϕ_ovl_ for the complexes [Eu(b*iso*Qcd)(H_2_O)_2_]^+^ and [Eu(*iso*QC3A)(H_2_O)_2_] were experimentally obtained by secondary methods described in the literature^89^ by measuring the visible emission spectrum of quinine bisulfate in 0.5 M H_2_SO_4_ solution, a fluorescence quantum yield reference sample (ϕ_s_ = 54.6%). ϕ_ovl_ for the complexes is calculated by the equation , where the u subscript refers to unknown (the sample under investigation) and s to the standard, and other symbols have the following meanings: ϕ_s_ is the overall quantum yield of the reference sample, *A* is the absorbance at the excitation wavelength, *F* is the integrated emission area across the band, and *n*’s are respectively the index of refraction of the solvent containing the unknown (*n*) and the standard (*n*_o_) at the sodium D line and the temperature of the emission measurement (see ESI, Figures S16 and S17). The measurements were repeated three times, and the averaged value is provided.

## Results and Discussion

### Structural and Thermodynamic Properties of the Complexes in Aqueous Solution

**Figure 1:**
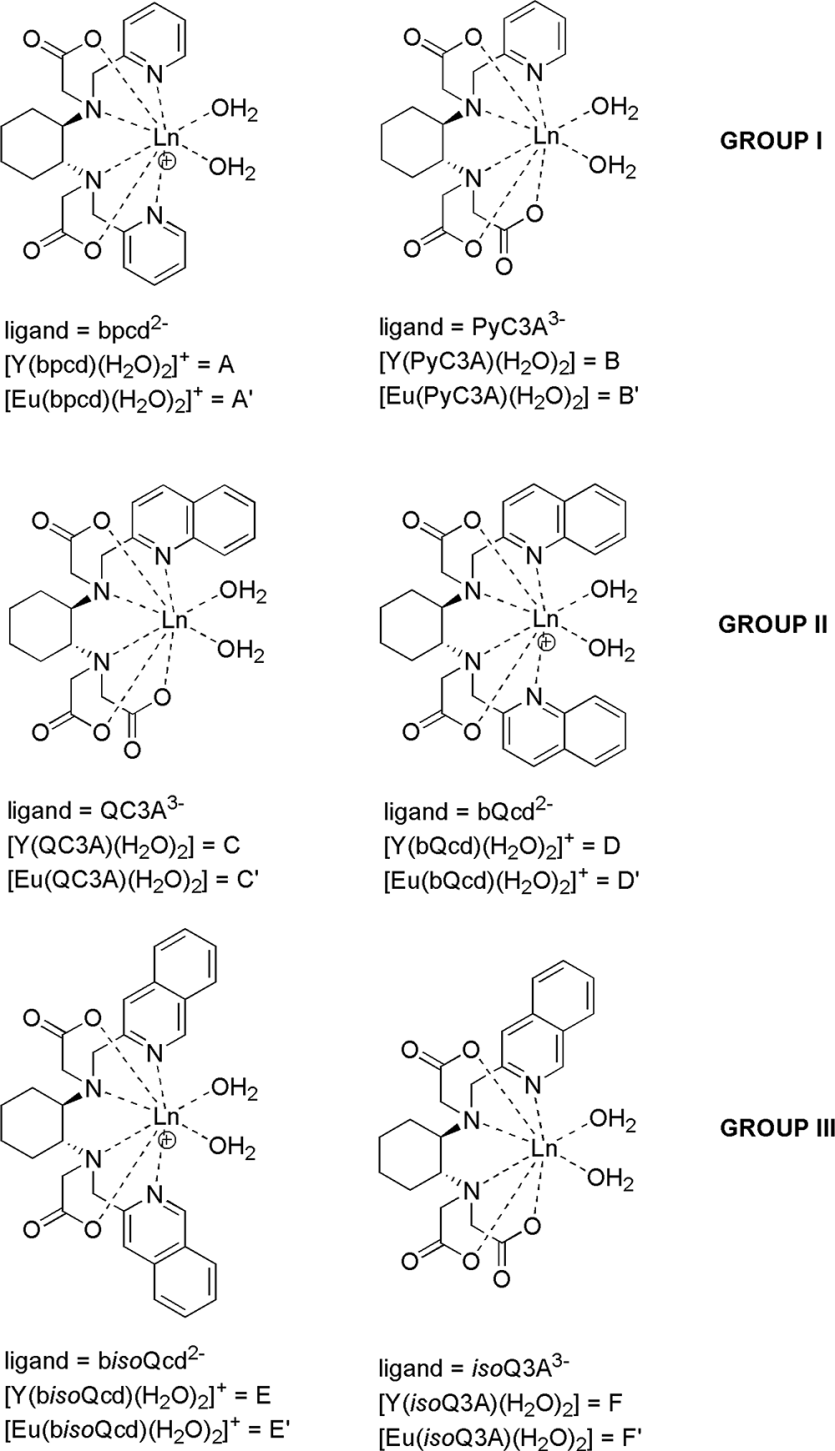
Chemical structures of the Ln(III) complexes studied in this work. Ln = Y(III) and Eu(III).

The chemical structures and the labels of the Eu(III) and Y(III) complexes described in this work are shown in Figure 1. The Eu(III) compounds were previously synthesized and spectroscopically characterized, and the Y(III) counterparts have been used exclusively as a computational structural model of the paramagnetic Eu(III) analogs, in view of the similar ionic radii of the two cations.^90,91^

For the sake of clarity, the investigated complexes have been divided into three groups, differing by the nature of the heteroaromatic ring: pyridine (group **I**), quinoline (group **II**), and *iso*quinoline (group **III**).

The protonation constants (log *K*) of the considered ligands are reported in Table 1. These values indicate that two fairly strong acidic and two weakly acidic sites are present. As previously discussed,^10−12,35^ the values for the first protonation constants of the ligands are in agreement with those reported for tertiary amines (log *K* ∼ 6.9–10.7, depending on the substituents),^92^ suggesting that the first protonation constant can be assigned to an aliphatic amino group, as previously reported for CDTA.^93^ The remaining protonation constants are ascribed to heteroaromatic (pyridine, quinoline, and isoquinoline) rings and acetate moieties.^10−12,35^

**Table 1:** Protonation Constants (log *K_j_*) and Formation Constants (log β) for the Complexes with Eu(III) Described in This Work at 298.15 K and μ = 0.1 M NaCl^a^

reaction	bpcd^b^	PyC3A^c^	bQcd^c^	QC3A^c^	b*iso*Qcd^d^	*iso*QC3A^e^	CDTA^f^
log *K_j_*							
L + H ⇆ HL	9.72	10.26	9.37	10.53	9.27	9.43	9.43
HL + H ⇆ H_2_L	5.87	6.33	5.85	6.29	5.86	7.37	6.01
H_2_L + H ⇆ H_3_L	2.94	3.67	3.46	3.60	3.43	3.32	3.68
H_3_L + H ⇆ H_4_L	2.22	2.01	1.79	2.81	1.62	2.16	2.51
log β							
L + Eu ⇆ EuL	11.19	15.68	9.97	12.55	10.53	14.63	19.60

a Additional protonation and formation constants data of the analog CDTA ligand are also reported for comparison. Charges are omitted for simplicity.

b Data from ref (35).

c Data from ref (12).

d Data from ref (11).

e Data from ref (10).

f CDTA: cyclohexanediaminetetraacetic acid; data taken from ref (95).

As for the speciation in aqueous solutions of the investigated complexes, at the physiological pH = 7.4, the ML species is largely predominant in all cases (>99%), with the exception of bpcd-based complexes for which a small amount (around 5%) of the neutral hydroxo [Eu(bpcd)(OH)(H_2_O)] complex was also detected.^35^

Because of the strong oxophilicity of the Ln(III) ions,^91^ the stability constants (log β) for the triacetate ligands [PyC3A, QC3A, and *iso*QC3A] are higher than their diacetate analogues [bpcd, bQcd, and b*iso*Qcd, respectively]. Besides, the stability constants of the Eu(III) complexes with the quinoline- and isoquinoline-substituted ligands are lower than for their pyridine analogues (bpcd and PyC3A). This result could be due to a weaker interaction of the quinoline moieties with the metal ion with respect to the pyridine ones and to the increased steric hindrance. From the perspective of the biological applications, the values of log β appear promising, in particular for the triacetate-based ligands (PyC3A, QC3A, and *iso*QC3A) whose stability is close to that of macrocyclic ligands possessing a similar coordination ability and already employed in molecular imaging applications (i.e., DO3A derivatives with log β values in the 18–21 range^94^).

**Figure 2:**
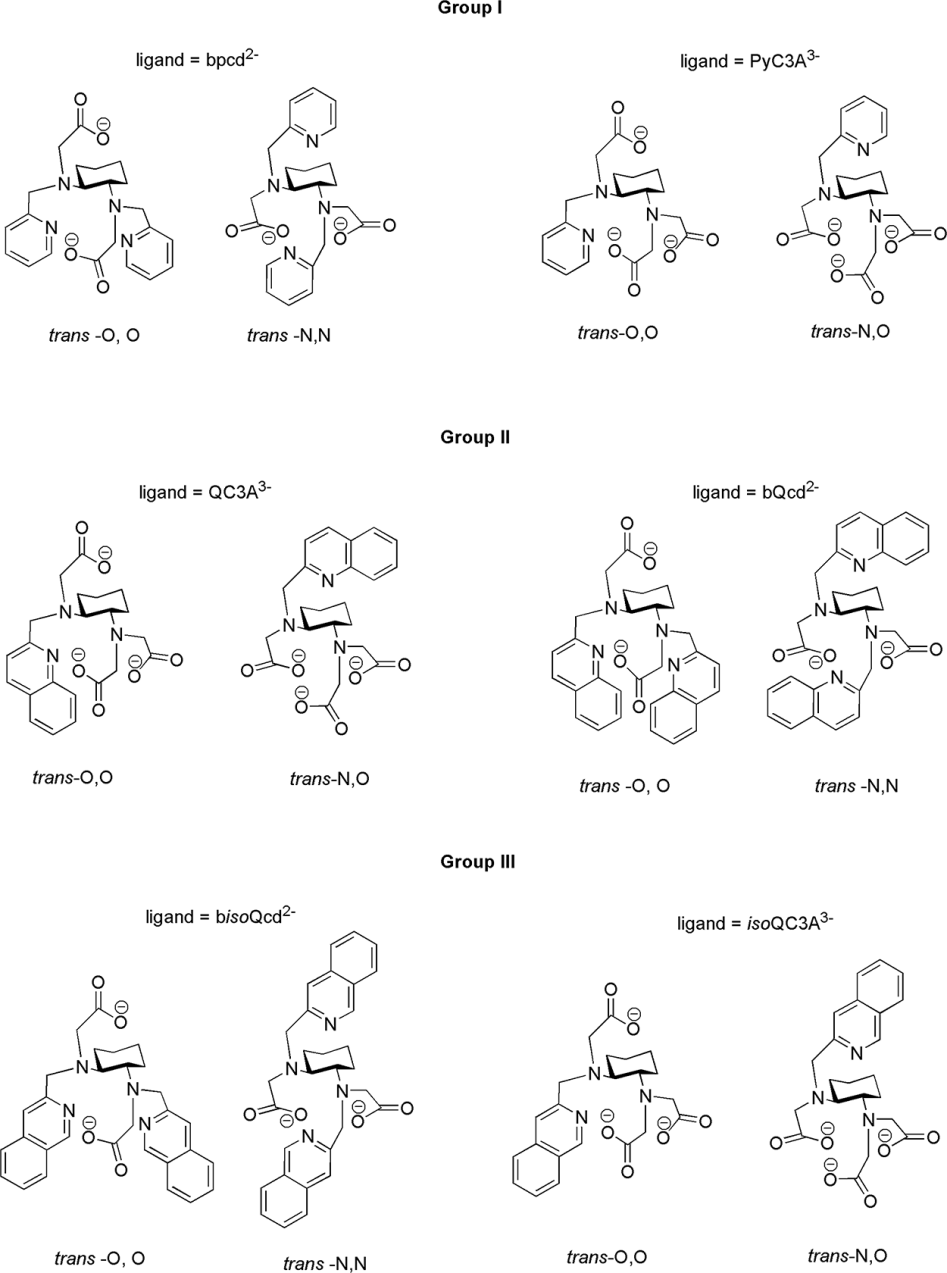
Possible coordination geometries of the ligands in this paper.

**Figure 3:**
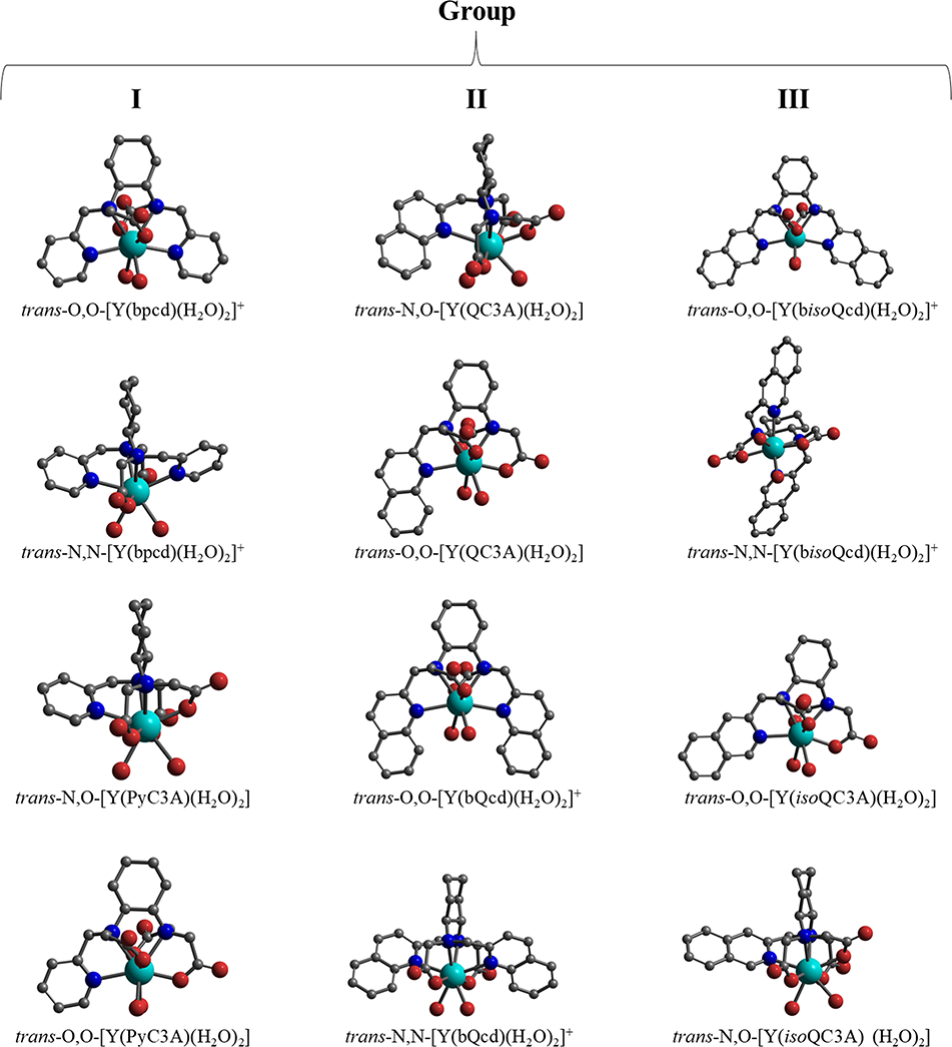
Group assignment, formula, and minimum energy DFT structure of the possible isomers of the Y(III) counterparts of the investigated Eu(III) complexes. Hydrogen atoms are removed for clarity.

Molecular models obtained from DFT calculations on the Y(III) counterparts (complexes **A–F**) of Eu(III) complexes show that Y(III) is in all cases eight-fold coordinated with six donor atoms belonging to the ligand and two oxygen atoms to coordinated water molecules, the general formula being [Y(L)(H_2_O)_2_], as illustrated in Figure 1. As two different coordination geometries, differing by the stereochemistry of the sp^3^ nitrogen atoms, are possible for each ligand (Figure 2), DFT calculations are performed on 12 species, whose labeling is reported in Table 2 while the minimum energy structures are shown in Figure 3.

**Table 2:** Labels, Group Membership, and Formula of the Complexes in This Paper

complex	isomer	formula	group
A	1	*trans*-*O*,*O*-[Y(bpcd)(H_2_O)_2_]^+^	**I**
	2	*trans*-*N*,*N*-[Y(bpcd)(H_2_O)_2_]^+^	
A′	1′	*trans*-*O*,*O*-[Eu(bpcd)(H_2_O)_2_]^+^	
	2′	*trans*-*N*,*N*-[Eu(bpcd)(H_2_O)_2_]^+^	
B	3	*trans*-*N*,*O*-[Y(PyC3A)(H_2_O)_2_]	
	4	*trans*-*O*,*O*-[Y(PyC3A)(H_2_O)_2_]	
B′	3′	*trans*-*N*,*O*-[Eu(Pyc3A)(H_2_O)_2_]	
	4′	*trans*-*O*,*O*-[Eu(PyC3A)(H_2_O)_2_]	
C	5	*trans*-*N*,*O*-[Y(QC3A)(H_2_O)_2_]	**II**
	6	*trans*-*O*,*O*-[Y(QC3A)(H_2_O)_2_]	
C′	5′	*trans*-*N*,*O*-[Eu(QC3A)(H_2_O)_2_]	
	6′	*trans*-*O*,*O*-[Eu(QC3A)(H_2_O)_2_]	
D	7	*trans*-*O*,*O*-[Y(bQcd)(H_2_O)_2_]^+^	
	8	*trans*-*N*,*N*-[Y(bQcd)(H_2_O)_2_]^+^	
D′	7′	*trans*-*O*,*O*-[Eu(bQcd)(H_2_O)_2_]^+^	
	8′	*trans*-*N*,*N*-[Eu(bQcd)(H_2_O)_2_]^+^	
E	9	*trans*-*O*,*O*-[Y(b*iso*Qcd)(H_2_O)_2_]^+^	**III**
	10	*trans*-*N*,*N*-[Y(b*iso*Qcd)(H_2_O)_2_]^+^	
E′	9′	*trans*-*O*,*O*-[Eu(b*iso*Qcd)(H_2_O)_2_]^+^	
	10′	*trans*-*N*,*N*-[Eu(b*iso*Qcd)(H_2_O)_2_]^+^	
F	11	*trans*-*O*,*O*-[Y(*iso*QC3A) (H_2_O)_2_]	
	12	*trans*-*N*,*O*-[Y(*iso*QC3A) (H_2_O)_2_]	
F′	11′	*trans*-*O*,*O*-[Eu(*iso*QC3A) (H_2_O)_2_]	
	12′	*trans*-*N*,*O*-[Eu(*iso*QC3A) (H_2_O)_2_]	

From a structural point of view, the increase in steric crowding when passing from pyridine- to the quinoline-substituted ligands is clearly seen upon inspection of the structures shown in Figure 3. This steric increase may reflect the change in Judd–Ofelt intensity parameters, more precisely in the general interpretation that Ω_4_ and Ω_6_ parameters correlate with the rigidity of a compound.^96,97^ Accordingly, an increase in the average Ω̅_4_ and Ω̅_6_ (Table S1) between groups is observed: Ω̅_4,6_(**I**) < Ω̅_4,6_(**II**) < Ω̅_4,6_(**III**).

From the resulting bond distances (Table 3), it emerges that the substitution of pyridine by quinoline or isoquinoline has nearly no effect on the Y(III)–*O*_acetate_ bonds (average variation, ΔPy → *Q* ∼ −0.001 and + 0.005 Å for the di- and tri-acids, respectively), and the Y(III)–*N*_amine_ distances are marginally affected (ΔPy → *Q* ∼ −0.019 and – 0.006 Å). It can also be noted that Y(III)–*O*_water_ bonds are slightly longer in the pyridine triacid isomers as could be expected on the basis of the decreased charge on the metal ion, while in the quinoline complexes, they are only slightly affected. However, the most remarkable finding is that the average Y(III)–*N*_heterocycle_ bond distance increases in the Py → *isoQ*→*Q* order. In the case of quinoline-based complexes, the increase of this bond distance is 0.11 Å with respect to the pyridine ligands (ΔPy → *Q* ∼ +0.11 Å), indicating the weaker interaction of quinoline with the metal ion; this possibly contributes to the drop of stability of the quinoline complexes compared to the pyridine analogues (on average ∼1.4 and 3.4 log units for the di- and tri-acetate ligands). However, it is expected that quinoline also has a notable impact on the solvation properties of the complex, which often have a strong influence on the stability.

**Table 3:** Selected Bond Distances (Å) of the Complexes in Table 2

group	formula (isomer)	Y–*O*_acetate_	Y–*N*_amine_	Y–*N*_heterocycle_	Y–*O*_water_
**I**	[Y(*trans*-*O*,*O* bpcd)(H_2_O)_2_]^+^ (**1**)	2.262	2.550	2.525	2.448
	[Y(*trans*-*N*,*N* bpcd)(H_2_O)_2_]^+^ (**2**)	2.292	2.610	2.503	2.492
	[Y(*trans*-*O*,*O* PyC3A)(H_2_O)_2_] (**3**)	2.286	2.568	2.550	2.474
	[Y(*trans*-*N*,*O* PyC3A)(H_2_O)_2_] (**4**)	2.300	2.595	2.546	2.539
**II**	[Y(*trans*-O,O QC3A)(H_2_O)_2_] (**5**)	2.286	2.574	2.654	2.458
	[Y(*trans*-*N*,*O* QC3A)(H_2_O)_2_] (**6**)	2.290	2.576	2.642	2.478
	[Y(*trans*-*O*,*O* bQcd)(H_2_O)_2_]^+^ (**7**)	2.268	2.557	2.661	2.464
	[Y(*trans*-*N*,*N* bQcd)(H_2_O)_2_]^+^ (**8**)	2.284	2.567	2.594	2.482
**III**	[Y(*trans*-*O*,*O* b*iso*Qcd)(H_2_O)_2_]^+^ (**9**)	2.262	2.577	2.526	2.451
	[Y(*trans*-*N*,*N* b*iso*Qcd)(H_2_O)_2_]^+^ (**10**)	2.283	2.565	2.614	2.463
	[Y(*trans*-*O*,*O iso*QC3A)(H_2_O)_2_] (**11**)	2.297	2.594	2.581	2.509
	[Y(*trans*-*N*,*O iso*QC3A)(H_2_O)_2_] (**12**)	2.297	2.579	2.553	2.471

### Spectroscopic Properties of the Complexes

As reported in Table 4, the experimental spectroscopic study on the Eu(III) complexes shows low to moderate intrinsic quantum yields (ϕ_int_ in the 5–10% range) for all compounds, which reflect the presence of MPR quenching, induced by water molecules in the proximity of the metal ion (hydration number *q* calculated by the Horrocks equation^98−100^), which is detrimental for the luminescence efficiency. This number, in the 2.5–2.8 range (Table 4), even if slightly larger than that found by DFT calculation (*q* = 2), is still in agreement with it. It must be pointed out that the *q* number is also sensitive to the presence of water in the outer coordination sphere of the metal ion. On the other hand, the presence of water molecules bound to Eu(III) is required to use these metal complexes as optical probes to sense biomolecules or bioanalytes. In fact, some of us reported how the displacement of this bound water by the target molecule is based on the optical sensing mechanism of important bioanalytes such as HCO_3_^–^,^12^ citrate,^10^ and bovine serum albumin (BSA).^11^

**Table 4:** Experimental Intrinsic ϕ_int_, Overall ϕ_ovl_ Quantum Yields (in %), and η_sens_ (in %) Reported for the Investigated Eu(III) Complexes^e^

group	complex	formula	ϕ_int_	η_sens_	ϕ_ovl_	*q*
**I**	A′	[Eu(bpcd)(H_2_O)_2_]^+^ (**1′** + **2′**)	10.0(1)^b^	61^b^	6.1(3)^b^	2.7(1)^a^
	B′	[Eu(PyC3A)(H_2_O)_2_] (**3′** + **4′**)	9.0(1)^b^	67^b^	6.0(3)^b^	2.7(1)^b^
**II**	C′	[Eu(QC3A)(H_2_O)_2_] (**5′** + **6′**)	9.9(1)^b^	40^b^	4.0(2)^b^	2.5(1)^b^
	D′	[Eu(bQcd)(H_2_O)_2_]^+^ (**7′** + **8′**)	9.0(1)^b^	29^b^	2.6(3)^b^	2.8(1)^b^
**III**	E′	[Eu(b*iso*Qcd)(H_2_O)_2_]^+^ (**9′** + **10′**)	5.0(1)^c^	26	1.3(1)^d^	2.8(1)^c^
	F′	[Eu(*iso*QC3A)(H_2_O)_2_] (**11′** + **12′**)	5.4(1)^c^	40	2.2(2)^d^	2.8(1)^c^

a Ref (35).

b Ref (12).

c Ref (10).

d The data were determined in this work by using the reference standard.

e *q* is the calculated number of water molecules.

**Figure 4:**
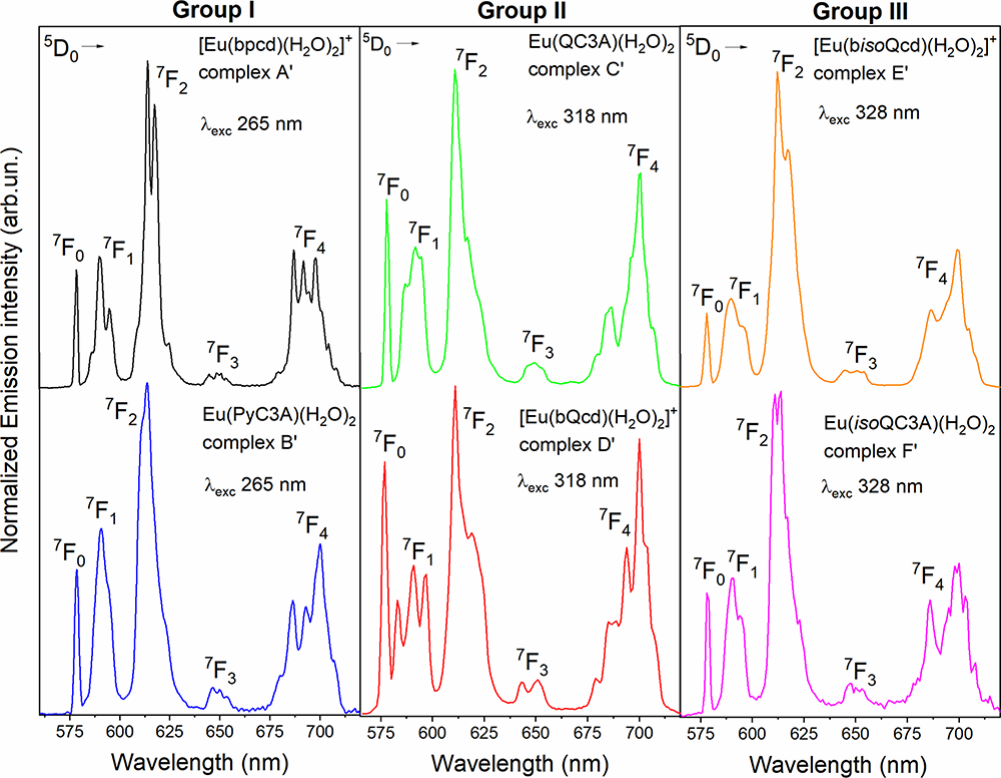
Room temperature luminescence emission spectra of the investigated Eu(III) complexes in aqueous solution (100 mM) upon excitation of the ligand (the λ_exc_ values are reported in the figure).

As for the overall quantum yield (ϕ_ovl_), we noticed a significant drop in its value in the case of the quinoline- and isoquinoline-based complexes (group **II** and **III** molecules) due to a worse efficiency of the sensitization process (η_sens_), which includes the ISC and the IET phenomena. η_sens_ is about 25% in the case of [Eu(b*iso*Qcd)(H_2_O)_2_]^+^ (**9′** + **10′**) complexes (Table 4), corresponding to an overall quantum yield of around 1%. The reasons for this behavior will be discussed in detail in the next sections of the paper. Finally, upon excitation into the ligand absorption bands (Figure 4), the luminescence of Eu(III) is sensitized and the emission spectra of the related complexes are all compatible with an emitting ion located in a non-centrosymmetric site. As it is well known in this case, the ^5^D_0_ → ^7^F_2_ hypersensitive transition dominates the spectra^101^ (Figure 4). Nevertheless, the ^5^D_0_ → ^7^F_4_ transitions are also very strong in comparison to ^5^D_0_ → ^7^F_1_ (used as a reference), suggesting that the Eu(III) ion is in a distorted cubic symmetry.^102,103^ The theoretical intensity parameters Ω_λ_ (Table S1) agree with the spectra in Figure 4.

Furthermore, all complexes exhibit a relatively strong ^5^D_0_ → ^7^F_0_ transition (particularly in the case of quinoline-based complexes). This feature is compatible with an axial character of the Eu(III) point symmetry.^101^*C*_n_, *C*_nv_, and *C*_s_ are the only possible point symmetries in the presence of sizeable intensity of the ^5^D_0_ → ^7^F_0_ transition,^104,105^ even if the *C*_s_ symmetry can be ruled out due to the presence of the chiral ligand. The relatively high intensity of ^5^D_0_ → ^7^F_0_ suggests that the studied compounds experience a strong *J*-mixing effect.^106−109^

### Excited States of the Ligands

From TD-DFT calculations, donor energy levels (S_1_ and T_1_) were obtained together with their respective donor–acceptor distances, fundamental quantities used in eq 4 (Δ value in *F*) and eqs 1–3 (*R*_L_), respectively.

Table 5 summarizes the calculated S_1_ and T_1_ energy positions, and Figures S1–S12 show the main compositions of these excited states. Once S_1_ and T_1_ states are both characterized as an electronic density displacement to the same portion in the compound (see unoccupied molecular orbitals in Figures S1–S12 in the ESI), the values of donor–acceptor distances (*R*_L_, the distance from the unoccupied molecular orbitals centroid to the Eu(III) ion) for IET involving S_1_ and T_1_ states can be considered the same for each compound (Table 5). For example, Figure S1 shows that the unoccupied molecular orbitals of the *trans*-*O*,*O*-[Y(bpcd)(H_2_O)_2_]^+^ (**1**) are localized at pyridine group for both S_1_ and T_1_ states. Thus, *R*_L_ is the distance between the center of the pyridine group to Y(III).

**Table 5:** Donor–Acceptor Distances *R*_L_ (in Å) and Energies of the S_1_ and T_1_ States (in cm^–1^) Estimated from TD-DFT Calculations

group	formula (isomer)	*R*_L_	S_1_	T_1_
**I**	**1**	3.91	39,412	31,435
	**2**	3.89	38,185	31,361
	**3**	3.93	38,546	31,368
	**4**	3.93	39,818	31,415
**II**	**5**	4.33	32,445	22,198
	**6**	4.32	33,271	22,696
	**7**	4.32	33,391	22,859
	**8**	4.28	31,818	22,052
**III**	**9**	5.04	32,087	21,843
	**10**	5.03	32,136	21,817
	**11**	5.08	32,000	21,995
	**12**	5.06	32,235	21,863

As discussed above, the 12 studied isomers were separated into three groups according to their distinct chemical characteristics (i.e., the nature of the heteroaromatic ring). This classification also finds parallelism in the values of the electronic level energy of the ligands involved in the IET process (S_1_ and T_1_ states; see Table 5): group **I** is composed of complexes **1–4** where both S_1_ and T_1_ are at higher energy (S_1_ around 38,900 cm^–1^ and T_1_ around 31,400 cm^–1^) than the complexes in groups **II** (complexes **5–8**) and **III** (complexes **9–12**). However, the complexes in group **I** present *R*_L_ shorter than complexes in groups **II** and **III**. As expected, this finding is connected to the shorter Y–*N*_heterocycle_ observed in the DFT structures of the [Y(bpcd)(H_2_O)_2_]^+^ and [Y(PyC3A)(H_2_O)_2_] complexes (Table 3). The complexes in groups **II** and **III** have similar donor energies (S_1_ around 32,400 cm^–1^ and T_1_ around 21,100 cm^–1^), but they are distinguished from each other by the value of *R*_L_ (Table 5), where complexes in group **III** present the highest values of *R*_L_ among all groups.

The calculated values of λ_M_ and SOC matrix elements allow estimating the *W*_ISC_ rates (Table S15) through the Marcus–Levich formalism.^45−47^ In addition, from the spin-orbit interaction, the decay lifetimes τ_S_ and τ_T_ were estimated (Eq. S12) from the dipole strengths of S_0_ → S_1_ and S_0_ → T_1_ excitations (*S*_S_ and *S*_T_ in Table S16).

A trend between the average of experimental ϕ̅_ovl_ and computationally obtained *W*_ISC_, 1/τ_S_, and 1/τ_T_ for the studied compounds can be observed (Figure S15). As these Eu(III) complexes present IET dominated by the T_1_ channel, a more effective *W*_ISC_ is expected to produce a higher ϕ_ovl_. On the same hand, a smaller 1/τ_T_ rate induces a higher T_1_ population to be transferred to Eu(III), also increasing the overall quantum yield (Figure S15). For these cases, 1/τ_S_ rates do not follow such trends. These results make clear the importance of the proper computation of *W*_ISC_, τ_S_, and τ_T_ quantities, highlighting the importance of the improvements shown in the present work.

### Intramolecular Energy Transfer

With the energy values of the S_1_ and T_1_ states and their donor–acceptor distances (*R*_L_) presented in Table 5, the IET rates can be calculated for each studied compound using eqs 1–5.

**Figure 5:**
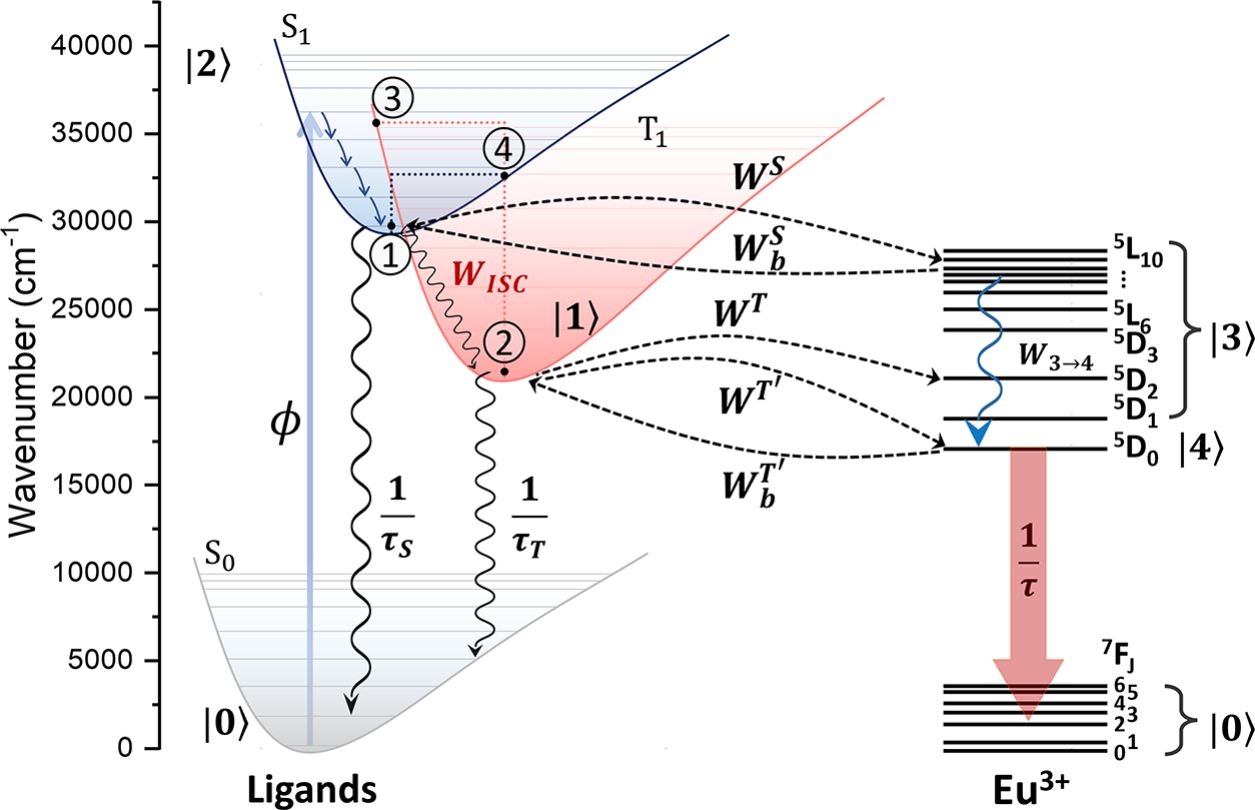
Schematic energy level diagrams for Eu-based complexes in this work. The S_1_ and T_1_ states for compounds **1′**–**4′** (group **I**) range from 38,185 to 39,818 cm^–1^ and from 31,361 to 31,435 cm^–1^, respectively, while those for **5′**–**12′** (groups **II** and **III**) range from 31,818 to 33,391 cm^–1^ and 22,052 to 22,859 cm^–1^, respectively (see Table 5). S_0_ is the ligand ground level. ϕ is the pumping rate. *W*_ISC_ is the S_1_ → T_1_ ISC rate (Table S15). The blue waved arrow represents the non-radiative decay from higher levels of Eu(III) to the emitting ^5^D_0_ while the black ones are the ligand’s decay lifetimes (τ_S_ and τ_T_, Table S16). *W*^S^ and *W*^T^ are the forward energy transfer rates from the S_1_ and T_1_ states, respectively. Compounds **5′**–**12′** present significant backward energy transfer rates to the T_1_ state (*W*_b_^T^). The notation |*N*⟩ (*N* = 0, 1, ..., 5) represents levels (or set of levels), and they will be used in the rate equation modeling to determine the theoretical value of the overall quantum yield (ϕ_ovl_). The points 1, 2, 3, and 4 in the S_1_ and T_1_ states are related to the Marcus reorganization energies to estimate the ISC rate for each group (see the Supporting Information file).

**Figure 6:**
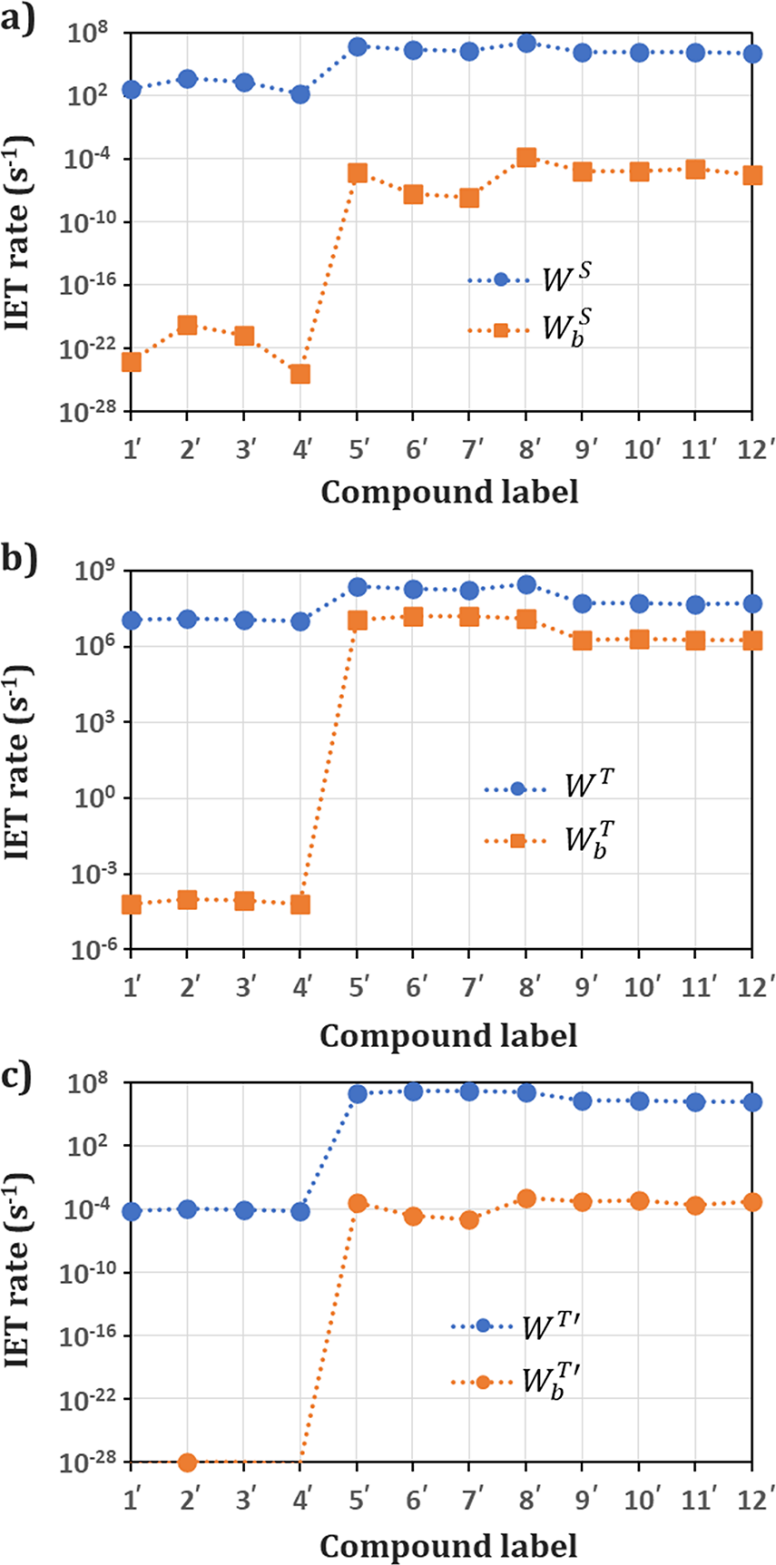
IET rates involving S_1_ (a) and T_1_ (b, c) states. Involvement of the ^5^D_0_ level is reported in (c). The blue circles represent forward rates (*W*^S^, *W*^T^, and *W*^T′^) and the orange squares the backward rates (*W*_b_^S^, *W*_b_^T^, and *W*_b_^T′^). With exception of *W*_b_^T^ for groups **II** and **III** (compounds from **5′** to **12′**), backward IET rates can be neglected in the rate equation model.

Figure 5 shows energy level diagrams that illustrate the ligand-to-Eu(III) energy transfer process. A total of 768 IET rates (64 for each complex, of which 32 for the forward and 32 for backward energy transfer) were obtained with non-zero contributions (see Tables S3–S14). The complete data for forward (*W*^S^ and *W*^T^) and backward (*W*_b_^S^ and *W*_b_^T^) IET rates, including the *W*_d – d_, *W*_d – m_, and *W*_ex_ mechanisms contributions (eqs 1–3), are presented in Tables S3–S14 while Figure 6 summarizes all IET rates.

Pathways from 1 to 16 (see Tables S3–S14) represent the contributions from S_1_, while pathways from 17 to 32 represent the T_1_ contributions. *W*^S^ and *W*^T^ are the sum over all forward pathways (ligand-to-Eu(III)) while *W*_b_^S^ and *W*_b_^T^ are the rates for the backward ones (Eu(III)-to-ligand).

The IET results indicate that the channel T_1_ → Eu(III) is the most effective energy transfer channel for all complexes. For group **I**, pathway 29 (from T_1_ to ^7^F_1_ → ^5^G_2_) has the highest forward IET rate, the exchange mechanism (*W*_ex_) being the dominating one in the overall IET process. Also, the backward IET rates (from Eu(III) levels to T_1_) can be neglected due to a high energy barrier involved (large values of |Δ|). Groups **II** and **III** have the forward rate dominated by pathway 18 (from T_1_ to ^7^F_0_ → ^5^D_1_, also governed by the exchange mechanism) and present a considerable *W*_b_^T^ rate dominated by pathway 29 (from ^7^F_1_ → ^5^G_2_ to T_1_). Thus, a consequence of these energies differences in the donor states, particularly regarding the T_1_ states, is reflected in the backward energy transfer, where the complexes in group **I** do not have a significant rate while groups **II** and **III** do (*W*_b_^T^ is around one order of magnitude lower than *W*^T^); see Figure 6. Compounds in Groups **II** and **III** also have a great contribution to the energy transfer directly to the Eu(III) ^5^D_0_ emitting level (sum of pathways 17 and 23, represented by the quantity *W*^T′^), which is a reflection of the ^7^F_1_ participation in the IET process.^110^

### Rate Equations and Overall Quantum Yield

Based on schematic energy level diagrams in Figure 5, the population kinetics can be described by the following general rate equations model for all complexes:
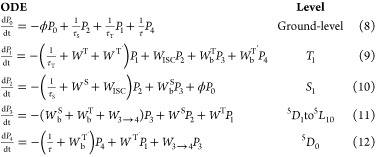
with *P_N_* representing the population of the |*N*⟩ level as depicted in Figure 5. τ_S_, τ_T_, and τ are the decay lifetimes of the S_1_, T_1_, and ^5^D_0_ levels. The values of these quantities for Ln-based complexes range from 10^–9^ to 10^–6^ s for τ_S_, 10^–6^ to 10^–3^ s for τ_T_, and ∼10^–3^ s for τ.^20,78,80^

Table S16 shows the values of τ_S_ and τ_T_ obtained from DFT calculations. As stressed before, *W*_ISC_ is the S_1_ → T_1_ ISC rate, and this rate was obtained for one complex of each group due to the complexity of the computational process. Hence, the *W*_ISC_ for isomers **1′**, **5′**, and **11′** were considered as representative examples of their respective groups (see Table S15 and related discussions in the Supporting Information file). These values were estimated according to the Marcus–Levich framework.^45−47^

*W*^T^ and *W*^S^ are the forward ligand-to-Eu(III) energy transfer rates while *W*_b_^T^ and *W*_b_^S^ are their respective backward energy transfer (Figure 6).

To obtain estimates of the Eu(III) ^5^D_0_ emitting level population, the ^5^D_0_ state (|4⟩ in Figure 5) was separated from Eu(III) upper levels (state |3⟩ in Figure 5). Thus, the quantities *W*_b_^T^′ and *W*^T^′ represent the backward energy transfer rates involving only the Eu(III) emitting level ^5^D_0_ and they are obtained by the sum of pathways 17 and 23 in Tables S3–S14.

The population simulations using eqs 8–12 consider the boundary conditions, which guarantee that the sum of the populations on all energy levels should be constant at any time *t*.^83,110^ Thus, the following relationship must be preserved (eq 13):
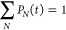
13where *P_N_*(*t*) is the population of state *N* at time *t* (0 ≤ *t* ≤ *t_f_*).

Table 6 summarizes all rates used in eqs 8–12. Since all compounds present T_1_ above the ^5^D_0_ level, the values of *W*_b_^T^′ (energy transfer from ^5^D_0_ to T_1_) are very low and can be neglected. On the other hand, the lower energy position of T_1_ provided high values of *W*^T^′ (energy transfer rates from T_1_ to ^5^D_0_ level) for compounds in groups **II** and **III**. The sensitizing process of the Eu(III) ion for all compounds is via the T_1_ state.

**Table 6:** IET Rates (in Units of S^–1^) Used in eqs 8–12 for each Eu(III) Complex

group	isomer	*W*^S^	*W*_b_^S^	*W*^T^	*W*^T^′	*W*_b_^T^	*W*_b_^T^′
**I**	**1′**	3.9 × 10^2^	10^–24^	1.2 × 10^7^	9.6 × 10^1^	6.1 × 10^–5^	10^–29^
	**2′**	4.5 × 10^3^	10^–20^	1.3 × 10^7^	1.2 × 10^2^	1.0 × 10^–4^	10^–28^
	**3′**	2.0 × 10^3^	10^–21^	1.2 × 10^7^	1.1 × 10^2^	8.5 × 10^–5^	10^–29^
	**4′**	1.6 × 10^2^	10^–25^	1.1 × 10^7^	9.4 × 10^1^	6.5 × 10^–5^	10^–29^
**II**	**5′**	5.1 × 10^6^	4.8 × 10^–6^	2.5 × 10^8^	4.1 × 10^7^	1.2 × 10^7^	4.1 × 10^–4^
	**6′**	2.2 × 10^6^	5.0 × 10^–8^	2.0 × 10^8^	2.8 × 10^7^	1.6 × 10^7^	2.6 × 10^–5^
	**7′**	1.9 × 10^6^	2.4 × 10^–8^	1.8 × 10^8^	2.3 × 10^7^	1.7 × 10^7^	1.0 × 10^–5^
	**8′**	1.0 × 10^7^	1.6 × 10^–4^	3.0 × 10^8^	5.2 × 10^7^	1.3 × 10^7^	1.1 × 10^–3^
**III**	**9′**	1.4 × 10^6^	6.9 × 10^–6^	5.5 × 10^7^	1.0 × 10^7^	2.0 × 10^6^	5.7 × 10^–4^
	**10′**	1.4 × 10^6^	6.7 × 10^–6^	5.6 × 10^7^	1.1 × 10^7^	2.0 × 10^6^	6.7 × 10^–4^
	**11′**	1.5 × 10^6^	1.2 × 10^–5^	4.8 × 10^7^	8.3 × 10^6^	1.9 × 10^6^	2.3 × 10^–4^
	**12′**	1.2 × 10^6^	3.2 × 10^–6^	5.2 × 10^7^	9.6 × 10^6^	1.9 × 10^6^	4.9 × 10^–4^

Figure S13 shows the transient curves of ^5^D_0_ and the ground levels for all 12 complexes. It can be noted that group **I** (complexes **1′**, **2′**, **3′**, and **4′**) has the highest ^5^D_0_ populations in comparison to the others. This is related to a higher *W*_ISC_ rate due to a lower S_1_–T_1_ energy gap (Δ*E*_ST_) and the absence of significant backward energy transfer (*W*_b_^S^, *W*_b_^T^, and *W*_b_^T^′). Despite the complexes in group **I** having shorter donor–acceptor distances (Table 5), they presented relatively low rates among the groups. However, the complexes in group **I** presented very high energies of the T_1_ state (∼31,300 cm^–1^), and this interesting condition allows the uncommon T_1_ → [^7^F_1_ → ^5^G_2_] pathway to be the dominating route (see pathway 29 in Tables S3–S5, and S6) and suppressing the Eu-to-ligand backward transfer. On the other hand, the complexes in groups **II** and **III** presented higher *W*^T^ rates (also the backward ones) due to T_1_ lower energies (∼22,000 cm^–1^) being in good resonance with the ^5^D_1_ level (see pathway 18 in Tables S7–S14).

With the values of populations in the steady-state regime (i.e., the ^5^D_0_ and ground-level populations at *t* > 2 ms in Figure S13, *P*_4_ and *P*_0_ respectively), the pumping rate, and radiative rates (Table S1), we obtain the theoretical value of the overall quantum yield (ϕ_ovl_, eq 7). All these values are summarized in Table 7.

**Table 7:** Populations of the ^5^D_0_ (*P*_4_) and Ground (*P*_0_) Levels (Unitless), Pumping (ϕ), and Radiative (*A*_rad_, Table S1) rates (in Units of S^–1^), and the Overall Quantum Yield ϕ_ovl_ (in %) for Each Eu(III) Complex

group	isomer	*P*_4_	*P*_0_	ϕ	*A*_rad_	ϕ_ovl_
**I**	**1′**	3.0 × 10^–2^	0.969	128.2	152.6	3.7
	**2′**	3.1 × 10^–2^	0.968	132.4	188.4	4.6
	**3′**	3.3 × 10^–2^	0.967	131.1	250.2	6.5
	**4′**	3.2 × 10^–2^	0.968	126.9	207.9	5.4
**II**	**5′**	3.8 × 10^–2^	0.962	155.8	207.1	5.2
	**6′**	3.6 × 10^–2^	0.964	151.9	204.4	5.0
	**7′**	3.6 × 10^–2^	0.964	151.4	179.3	4.4
	**8′**	4.3 × 10^–2^	0.957	158.8	120.4	3.4
**III**	**9′**	6.9 × 10^–3^	0.993	157.5	132.1	0.6
	**10′**	1.1 × 10^–2^	0.989	157.3	186.4	1.4
	**11′**	2.2 × 10^–2^	0.978	157.9	263.7	3.7
	**12′**	8.3 × 10^–3^	0.992	156.8	236.2	1.3

It is important to mention that the ϕ_ovl_ value, in downconversion systems, is independent of the excitation power density since if ϕ(the pumping rate) is *n*-fold increased, the emitting level population *P*_4_ increases while the ground state is more depleted *P*_0_, but the ratio *P*_4_/*P*_0_ is also *n*-fold increased. In other words, the ϕ value in the rate equation model does not affect the ϕ_ovl_ calculations.^14^

It can be noted that the compounds in group **I** present higher values of theoretical ϕ_ovl_, as compound **3′** reaches ϕ_ovl_ = 6.5%. On the other hand, compounds **9′** and **10′** have lower values of ϕ_ovl_ among all compounds due to a low radiative rate (*A*_rad_) and ^5^D_0_ population (*P*_4_), quantities ascribed to the small values of Ω_λ_ (mainly the Ω_2_, Table S1) and high backward IET rates, respectively. These results are in complete agreement with those determined experimentally for complexes **9′** and **10′** (Table 4). As for the values of the ϕ_ovl_ for the different complexes, we also noticed a good agreement between theoretical and experimental data (see Tables 4 and 7), which confirms the effectiveness of the theoretical modeling without the inclusion of experimental data. A question can be raised regarding whether a better luminescent performance of these compounds could be obtained in the direct excitation of the Eu^3+^ levels (e.g., ^5^D_0_ and ^5^D_1_). Of course, this avoids non-radiative losses in the process and the ϕ_ovl_ values tend to ϕ_int_. However, this kind of excitation involves a drastic decrease by order of magnitudes of the molar absorption extinction coefficient ε. In this way, the brightness (*B* = ε · ϕ_ovl_) is lower in comparison to the excitation through singlet states of the ligands. While ϕ_ovl_ varies between 0 and 1, ε may vary by orders of magnitude. Therefore, it is convenient to optimize energy transfer processes beneficially for the sake of higher brightness.

## Conclusions

In the present work, we have theoretically analyzed 12 Eu(III)-based complexes (all the possible geometric isomers were considered) with the general formula [Eu(L)(H_2_O)_2_]^+^ (where L = bpcd^2–^; bQcd^2–^and b*iso*Qcd^2–^) and Eu(L)(H_2_O)_2_ (where L = PyC3A^3–^; QC3A^3–^ and *iso*QC3A^3–^). TD-DFT calculations revealed the ligand excited donor states and the donor–acceptor distance (*R*_L_). Thus, a total of 768 Ligand↔Eu(III) IET rates were calculated considering the transition of both S_1_ and T_1_ as donor states (localized in the ligands) and 16 acceptors, involving ^7^F_0_ and ^7^F_1_ as initial states and ^5^D_0_, ^5^D_1_, ^5^D_2_, ^5^D_3_, ^5^L_6_, ^5^L_7_, ^5^G_2_, ^5^G_3_, ^5^G_4_, ^5^G_6_, ^5^D_4_, ^5^G_5_, ^5^L_8_, ^5^D_4_, ^5^L_9_, and ^5^L_10_ as final states. The IET rates are affected by (i) donor–acceptor distances (*R*_L_); (ii) the donor (S_1_ and T_1_) state energy position; and (iii) selection rules on *J* quantum numbers (|*J* – *J* ′ | ≤ *λ* ≤ *J* + *J*′ for dipolar and Δ*J* = *J* – *J* ′ = 0, ± 1 for exchange mechanisms). Once (i) and (ii) are associated with the donor excited states, good theoretical treatment is necessary for an appropriate description of IET rates and, therefore, a rate equation model, which enables the prediction of the emitting level population. The energy transfer process for all studied compounds is dominated via the T_1_ state, with *W*^T^ in some cases more than 10^4^ times higher than *W*^S^ (compounds **1′** and **4′**, Figure 6). The reason for the higher efficiency of the sensitization process (η_sens_), around 60–65% in the case of the complexes of group **I** is connected to a shorter *R*_L_ combined with a high ISC rates (*W*_ISC_) and a negligible backward energy transfer from Eu(III) to S_1_ and T_1_ level of the ligands. On the contrary, the worst sensitization efficiency (around 25%) recorded in the case of group **II** and **III** complexes (in particular for [Eu(b*iso*Qcd)(H_2_O)_2_]^+^, isomers **9′** and **10′**) seems to be due to a significant rate of the Eu(III)-to-T_1_ backward energy transfer process (*W*_b_^T^≈ 2 × 10^6^ s^–1^) to a lower *W*_ISC_ and to a larger *R*_L_. To sum up, the forward IET mechanisms mainly involve the Eu(III) ^5^G_2_ level for the compounds of the group **I** and ^5^D_1_ and ^5^D_0_ levels for the compounds of groups **II** and **III**. The backward IET process, significant only for these last complexes, mainly involves the ^5^G_2_ level. All the IET processes are dominated by the exchange mechanism.

For the first time, theoretical overall quantum yields (ϕ_ovl_) were calculated without the introduction of experimental parameters (e.g., decay lifetimes, Judd–Ofelt parameters, energy levels). This was achieved using a blend of DFT, Judd–Ofelt theory, IET theory (including calculated ISC from SOC-TD-DFT), and rate equation modeling. The present work represents a milestone in a detailed description of the luminescence properties of Ln-based chelates. Such a combined experimental/computational study provides deep knowledge of all the variables governing the IET mechanism, and we believe that its extension to other luminescent Ln(III) complexes can make easier the design of new and ever more efficient chromophoric antennae to sensitize the Ln(III) luminescence.

## Supporting Information Available

Calculation of the intensity parameters and radiative rates, the matrix elements, the IET and ISC rates, and the decay lifetimes of S_1_ and T_1_ states; pictures of the molecular orbitals composition of S_1_ and T_1_ states; and luminescence emission spectra for the calculation of the experimental quantum yields (PDF)
