# Supplementary material for: Dynamics of the Energy Transfer Process in Eu(III) Complexes Containing Polydentate Ligands Based on Pyridine, Quinoline, and Isoquinoline as Chromophoric Antennae
Source: Inorg Chem. 2022 Oct 6;61(41):16333–46. doi: 10.1021/acs.inorgchem.2c02330 (PMC9580001; doi:10.1021/acs.inorgchem.2c02330)
Supplement: Supplementary file 1 — ic2c02330_si_001.pdf [file ic2c02330_si_001.pdf]

Supporting Information

Dynamics of the energy transfer process in Eu(III) complexes containing polydentate ligands based on pyridine, quinoline, and *iso*quinoline as chromophoric *antennae*.

Albano N. Carneiro Neto,[1,*] Renaldo T. Moura Jr.,[2,3] Luís D. Carlos,[1] Oscar L. Malta,[4] Martina Sanadar,[5] Andrea Melchior,[5,*] Elfi Kraka,[3] Silvia Ruggieri,[6] Marco Bettinelli,[6] and Fabio Piccinelli[6,*]

[1] Physics Department and CICECO-Aveiro Institute of Materials, University of Aveiro, 3810-193 Aveiro, Portugal

[2] Department of Chemistry and Physics, Federal University of Paraíba, 58397-000 Areia, Brazil

[3] Department of Chemistry, Southern Methodist University, 75275-0314 Dallas, United States

[4] Department of Fundamental Chemistry, Federal University of Pernambuco, 50740-560 Recife, Brazil

[5] Dipartimento Politecnico di Ingegneria e Architettura, Laboratorio di Tecnologie Chimiche, University of Udine, 33100 Udine, Italy

[6] Luminescent Materials Laboratory, Department of Biotechnology, University of Verona and INSTM, UdR Verona, 37134 Verona, Italy

*** Corresponding Authors:**
Albano N. Carneiro Neto: albanoneto@ua.pt
Andrea Melchior: andrea.melchior@uniud.it
Fabio Piccinelli: fabio.piccinelli@univr.it

Contents

S1. Intensity parameters and radiative rates 2
S2. Matrix elements calculations........................... 4
S3. Molecular orbitals 6
Figure S1 6
Figure S2 6
Figure S3 7
Figure S4 7
Figure S5 8
Figure S6 8
Figure S7 9
Figure S8 9
Figure S9 10
Figure S10 10
Figure S11 11
Figure S12 11
Figure S13 12
S4. IET rates 13
Table S3... 13
Table S4... 14
Table S5 15
Table S6 16
Table S7 17
Table S8 18
Table S9 19
Table S10 20
Table S11 21
Table S12 22
Table S13 23
Table S14 24
S5. ISC rates and decay lifetimes of the S_1 and $T_1$25
Figure S14..25
Table S15 26
Table S16 26
Figure S15..27
S6. Experimental quantum yields 28
Figure S16..28
Figure S17..28
References...29

S1. Intensity parameters and radiative rates

The forced electric dipole (FED – Judd-Ofelt theory) and dynamic coupling (DC) mechanisms are the most responsible for the $4f$-$4f$ intensities when the lanthanide occupies a non-centrosymmetric site [1–3]. The theoretical expressions here used for the intensity parameters, Ω_λ^{theo}, have been described in detail in several references [4–6]. Here, a brief recall of them is given:

$$\Omega_\lambda^{theo} = (2\lambda + 1)\sum_{t,p}\frac{\left|B_{\lambda tp}\right|^2}{2t+1} \quad , \qquad B_{\lambda tp} = B_{\lambda tp}^{FED} + B_{\lambda tp}^{DC} \tag{S1}$$

where,

$$B_{\lambda tp}^{FED} = \frac{2}{\Delta E}\langle r^{t+1}\rangle \Theta(t,\lambda)\left(\frac{4\pi}{2t+1}\right)^{\frac{1}{2}}\sum_j \frac{e^2\rho_j g_j \left(2\beta_j\right)^{t+1}}{R_j^{t+1}}\left(Y_p^{t*}\right)_j \tag{S2}$$

$$B_{\lambda tp}^{DC} = -\left[\frac{(\lambda+1)(2\lambda+3)}{(2\lambda+1)}\right]^{\frac{1}{2}}\langle r^\lambda\rangle\langle f\|C^{(\lambda)}\|f\rangle\left(\frac{4\pi}{2t+1}\right)^{\frac{1}{2}}\sum_j\frac{\left[\left(2\beta_j\right)^{t+1}\alpha_{OP,j}+\alpha'_j\right]}{R_j^{t+1}}\left(Y_p^{t*}\right)_j\delta_{t,\lambda+1} \tag{S3}$$

with t and p being the ranks and components of the spherical harmonics $\left(Y_p^{t*}\right)$, $\langle f\|C^{(2)}\|f\rangle = -1.366$, $\langle f\|C^{(4)}\|f\rangle = 1.128$, $\langle f\|C^{(6)}\|f\rangle = -1.27$, ρ is the overlap integral between the valence subshells of the ligating atom and the $4f$ subshell of the lanthanide ion, $\beta = 1/(1 \pm \rho)$ is a parameter that defines the centroid of the electronic density of the chemical bond Ln–X (X= ligating atom), α' is the effective polarizability from each ligand around the Ln^{3+} and reasonable values of α' = 0.3 and 0.5 Å^3 were considered for N and O atoms of the ligands while α' = 0.2 Å^3 was set for O atoms of coordinated water molecules [7]. g is the charge factor and, in the present work, it was assumed values of $g = 1$ [8]. Eq. (S2) is the Simple Overlap Model (SOM) [5] expression for the odd component of the ligand field. Eq. (S3) is the Bond Overlap Model (BOM) for the polarizability dependent term (Dynamic Coupling mechanism) of 4f-4f transitions [6].

The overlap polarizabilities α_{OP} are quantities related to the covalent fraction of a chemical bond [6,9–11], and it is given by:

$$\alpha_{OP} = \frac{e^2\rho^2R^2}{2\Delta\varepsilon} \tag{S4}$$

where e is the electron charge, R is the length of the bond, and $\Delta\varepsilon$ is excitation energy associated with the chemical bond.

The radiative rates A_{rad} (also known as Einstein coefficient for spontaneous emission) were obtained using the following expression [12]:

$$A_{rad} = \sum_J A_{J'\to J} = \frac{4\pi}{3\hbar c^3}\sum_J \left(\omega_{J'\to J}\right)^3 \left(\frac{n(n^2+2)^2}{9} S_{ED}(J' \to J) + n^3 S_{MD}(J' \to J)\right) \tag{S5}$$

where $\omega_{J'\to J}$ is the angular frequency and n is the index of refraction of the medium (considered as n = 1.333 for all studied complexes in aqueous solution [13]). $S_{ED}(J' \to J)$ and $S_{MD}(J' \to J)$ are the electric and magnetic dipole strengths of the $J' \to J$ transition (in units of e^2). Once we are interested in Eu^{3+} emissions from the $^5D_0$ level, the A_{rad} will be the sum of the contributions of $^5D_0 \to {}^7F_J$ ($J = 1, 2, 4,$ and 6) transitions. By selection rules, the $^5D_0 \to {}^7F_1$ is allowed by magnetic dipole and forbidden by the electric dipole (*i.e.* $S_{MD}(0 \to 1) \neq 0$ and $S_{ED}(0 \to 1) = 0$) whilst the others transitions ($^5D_0 \to {}^7F_2$, $^5D_0 \to {}^7F_4$, and $^5D_0 \to {}^7F_6$) are allowed by the electric dipole and forbidden by the magnetic one (*i.e.* $S_{MD}(0 \to 2,4,6) = 0$ and $S_{ED}(0 \to 2,4,6) \neq 0$). The magnetic and electric dipole strengths can be calculated as:

$$S_{MD} = \frac{1}{2J'+1}\left(\frac{\mu_B}{ec}\right)^2 \langle \psi J \| \mathbf{L} + g_S \mathbf{S} \| \psi' J' \rangle^2 \tag{S6}$$

$$S_{ED} = \frac{1}{2J'+1}\sum_{\lambda=2,4,6} \Omega_\lambda \langle \psi J \| U^{(\lambda)} \| \psi' J' \rangle^2 \tag{S7}$$

where $\langle \psi J \| U^{(\lambda)} \| \psi' J' \rangle^2$ are the squared reduced matrix elements ($\langle {}^7F_\lambda \| U^{(\lambda)} \| {}^5D_0 \rangle^2 = 0.0032, 0.0023,$ and 0.0002 for $\lambda = 2, 4,$ and 6, respectively) [14] and $\langle {}^7F_1 \| \mathbf{L} + g_S \mathbf{S} \| {}^5D_0 \rangle^2 = 0.116$ for the Eu^{3+} $^5D_0 \to {}^7F_1$ transitions calculated from Ofelt's wavefunctions in the intermediate coupling scheme [15–17]. μ_B is Bohr's magneton and c is the speed of light in a vacuum.

The values of Ω_λ^{FED}, obtained using only Eq. (S3) into Eq. (S1), are presented in Table S1. It is worth emphasizing that only the FED contributions are used in the calculations of IET rates involving dipole interaction once opposite parity configuration mixing by the odd components of the ligand field is considered [12,18], just like treated in the original Judd-Ofelt theory. Table S1 also presents the values of the total Ω_λ (with both FED and DC contributions) as well as the radiative rates for each compound. These calculations were done using the JOYSpectra web platform [19].

Table S1. Calculated values of $\mathbf{\Omega_\lambda}$ and $\mathbf{\Omega_\lambda^{FED}}$ (both in units of 10^{-20} cm^2) and radiative rates A_{rad} (in units of s^{-1}) for all studied systems. $\bar{\mathbf{\Omega}}_\lambda$ are the average values of $\mathbf{\Omega_\lambda}$ and $\mathbf{\Omega_\lambda^{FED}}$ for each group.

Label	Ω_2	Ω_2^{FED}	Ω_4	Ω_4^{FED}	Ω_6	Ω_6^{FED}	A_{rad}
**1’**	3.15	0.01	4.61	0.25	2.03	0.56	152.6
**2’**	5.12	0.11	4.07	0.23	2.75	0.60	188.4
**3’**	7.96	0.19	4.31	0.32	3.36	0.77	251.4
**4’**	5.16	0.17	5.84	0.39	3.86	0.93	207.9
$\bar{\Omega}_\lambda$	5.35	0.12	4.71	0.30	3.00	0.72	
**5’**	5.49	0.27	5.12	0.33	3.24	0.80	163.1
**6’**	5.24	0.28	5.34	0.37	3.85	0.91	204.4
**7’**	4.24	0.09	4.94	0.31	3.32	0.80	179.7
**8’**	2.10	0.10	3.55	0.22	3.01	0.66	119.6
$\bar{\Omega}_\lambda$	4.27	0.19	4.74	0.31	3.36	0.79	
**9’**	2.22	0.08	4.47	0.27	2.60	0.67	131.7
**10’**	4.24	0.05	5.61	0.35	3.74	0.90	187.1
**11’**	8.11	0.23	5.29	0.35	3.89	0.86	265.2
**12’**	6.78	0.17	5.34	0.35	3.30	0.78	237.4
$\bar{\Omega}_\lambda$	5.34	0.13	5.18	0.33	3.38	0.80	

S2. Matrix elements calculations

The matrix elements of the spin ($\boldsymbol{S}$) operator between the states $\psi LSJ \rightarrow \psi' L' S' J'$ can be calculated in the following way:

$$\langle \psi LSJ \| S \| \psi' L' S' J' \rangle = \sum_i a_i \langle \psi LS || S || \psi' L' S' \rangle_i \quad \text{(S8)}$$

The matrix element above can be obtained using the intermediate coupling scheme [15]. In the present work, these matrix elements were expanded as a sum over the product of eigenfunctions $|\xi LSJ\rangle$ and $|\xi L'S'J'\rangle$ coefficients ($a_i = \langle \xi LSJ | \xi L'S'J' \rangle$).

$$\langle \psi LS || S || \psi' L' S' \rangle = (-1)^{L+S+J'+1}[(2J+1)(2J'+1)S(S+1)(2S+1)]^{\frac{1}{2}} \begin{Bmatrix} S & J & L \\ J' & S & 1 \end{Bmatrix} \quad \text{(S9)}$$

The quantities $\begin{Bmatrix} j_1 & j_2 & j_3 \\ j_4 & j_5 & j_6 \end{Bmatrix}$ are the 6-j symbols and their subsets (j_1, j_2, j_3), (j_1, j_5, j_6), (j_4, j_2, j_6) and (j_4, j_5, j_3) must satisfy the triangle rule or the 6-j symbol will be null.

Table S2 summarizes all the matrix elements for Eu^{3+} transitions considered in the present work.

Table S2. Values of the squared reduced matrix elements. The values of $\langle \|U^{(K)}\| \rangle^2$ were taken from Ref. [14].

Transition	$\langle \|U^{(2)}\| \rangle^2$	$\langle \|U^{(4)}\| \rangle^2$	$\langle \|U^{(6)}\| \rangle^2$	$\langle \|S\| \rangle^2$
$^7F_0 \rightarrow {}^5D_0$	8.0×10^{-6} *	0	0	0
$^7F_0 \rightarrow {}^5D_1$	0	0	0	2.73×10^{-2}
$^7F_0 \rightarrow {}^5D_2$	0.0008	0	0	0
$^7F_0 \rightarrow {}^5L_6$	0	0	0.0153	0
$^7F_0 \rightarrow {}^5G_6$	0	0	0.0037	0
$^7F_0 \rightarrow {}^5D_4$	0	0.0011	0	0
$^7F_1 \rightarrow {}^5D_0$	0	0	0	0.117
$^7F_1 \rightarrow {}^5D_1$	0.0025	0	0	2.81×10^{-5}
$^7F_1 \rightarrow {}^5D_2$	0	0	0	4.58×10^{-3}
$^7F_1 \rightarrow {}^5D_3$	0.0004	0.0012	0	0
$^7F_1 \rightarrow {}^5L_6$	0	0	0.0091	0
$^7F_1 \rightarrow {}^5L_7$	0	0	0.0181	0
$^7F_1 \rightarrow {}^5G_2$	0	0	0	1.28×10^{-2}
$^7F_1 \rightarrow {}^5G_3$	0.0002	0.0012	0	0
$^7F_1 \rightarrow {}^5G_6$	0	0	0.0049	0
$^7F_1 \rightarrow {}^5G_5$	0	0.0004	0.0097	0

* Considering *J*-mixing effect of 5%.

S3. Molecular orbitals

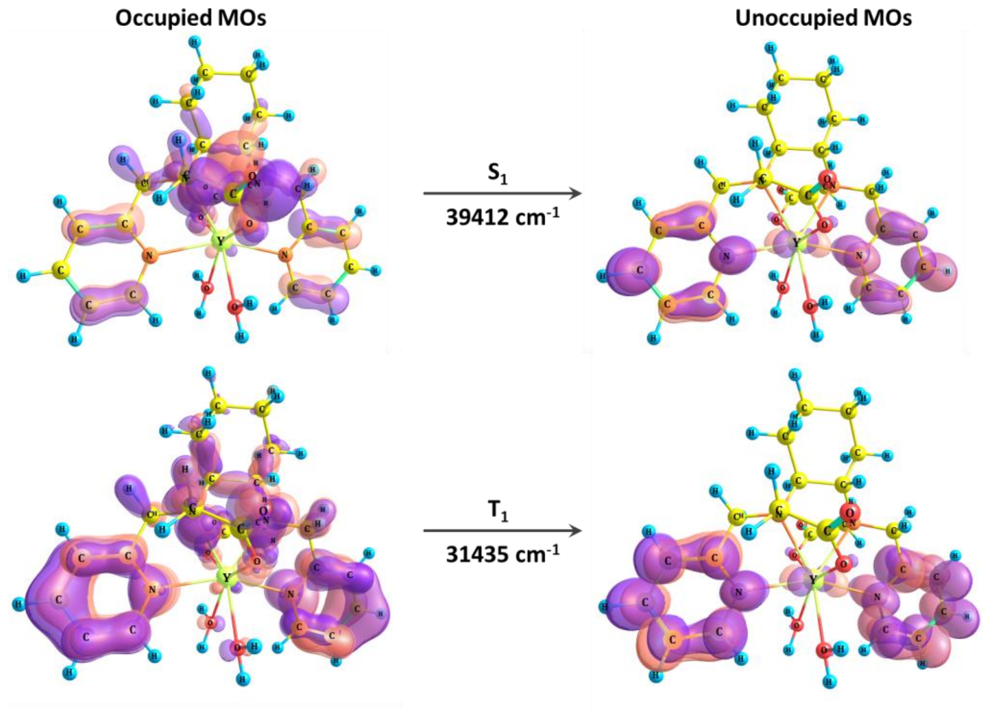

Figure S1. Kohn−Sham molecular orbitals composition of S_1 and T_1 states for *trans*-O,O-$[Y(bpcd)(H_2O)_2]^+$ (**1**).

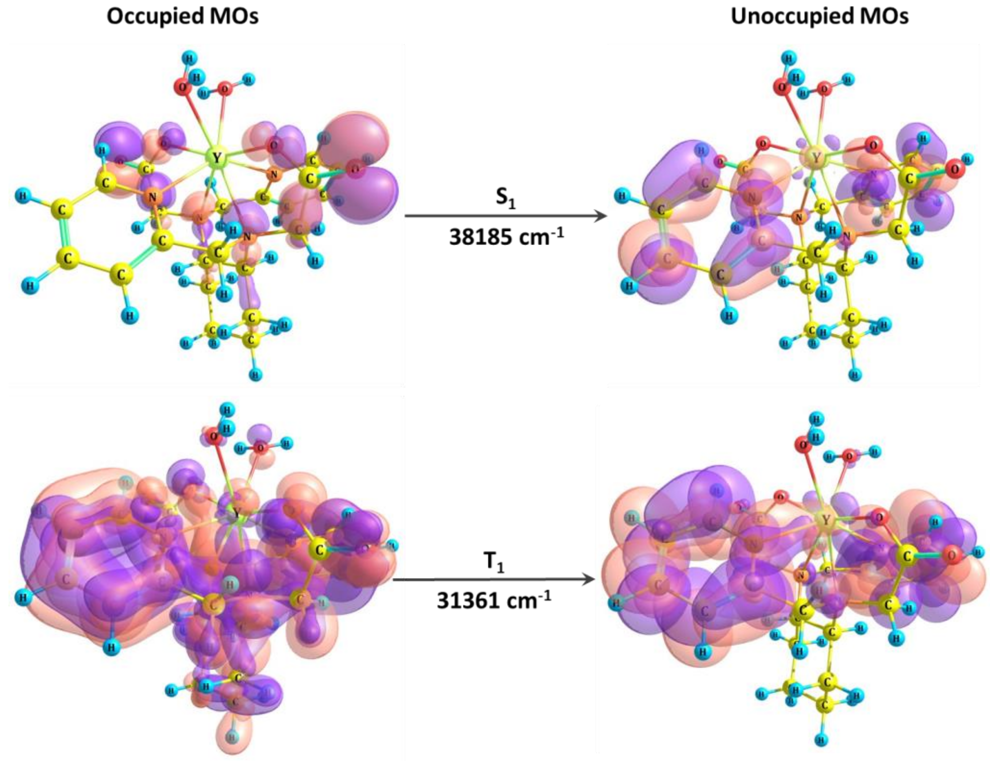

Figure S2. Kohn−Sham molecular orbitals composition of S_1 and T_1 states for *trans*-N,N-$[Y(bpcd)(H_2O)_2]^+$ (**2**).

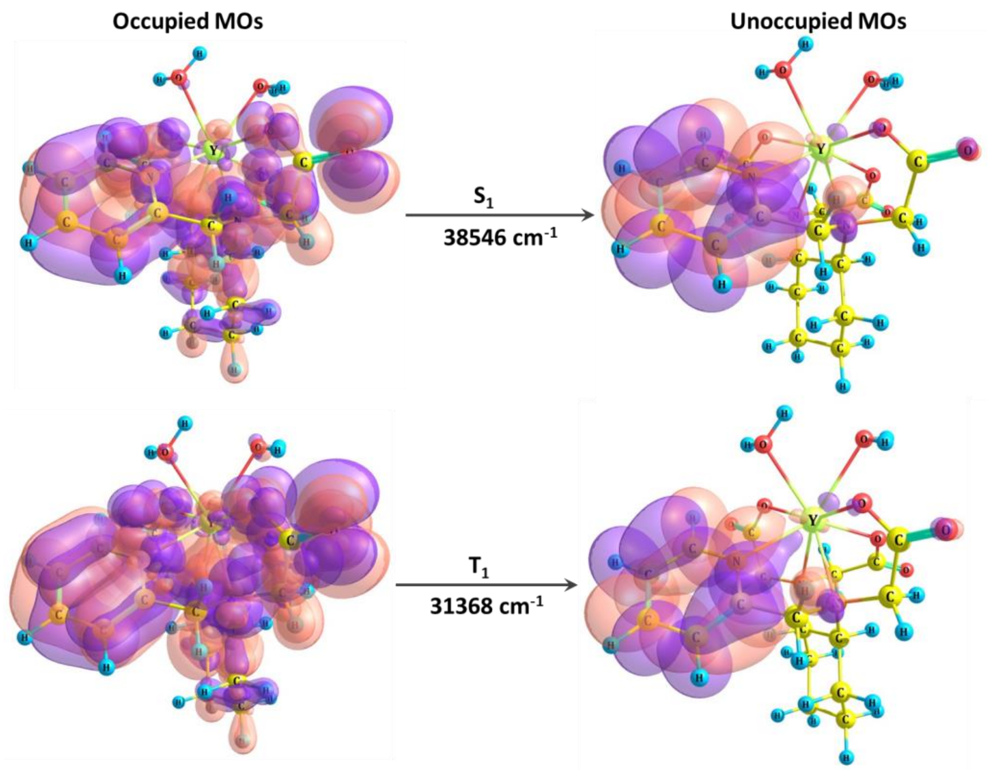

Figure S3. Kohn−Sham molecular orbitals composition of S_1 and T_1 states for *trans*-N,O-[Y(PyC3A)(H_2O)$_2$] (**3**).

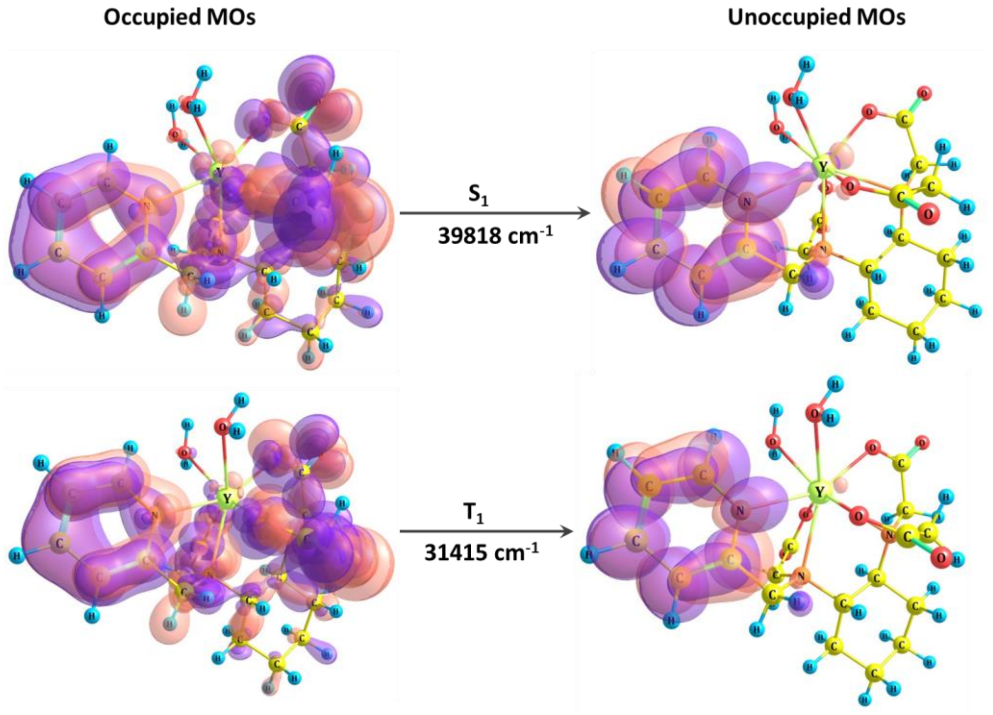

Figure S4. Kohn−Sham molecular orbitals composition of S_1 and T_1 states for *trans*-O,O-[Y(PyC3A)(H_2O)$_2$] (**4**).

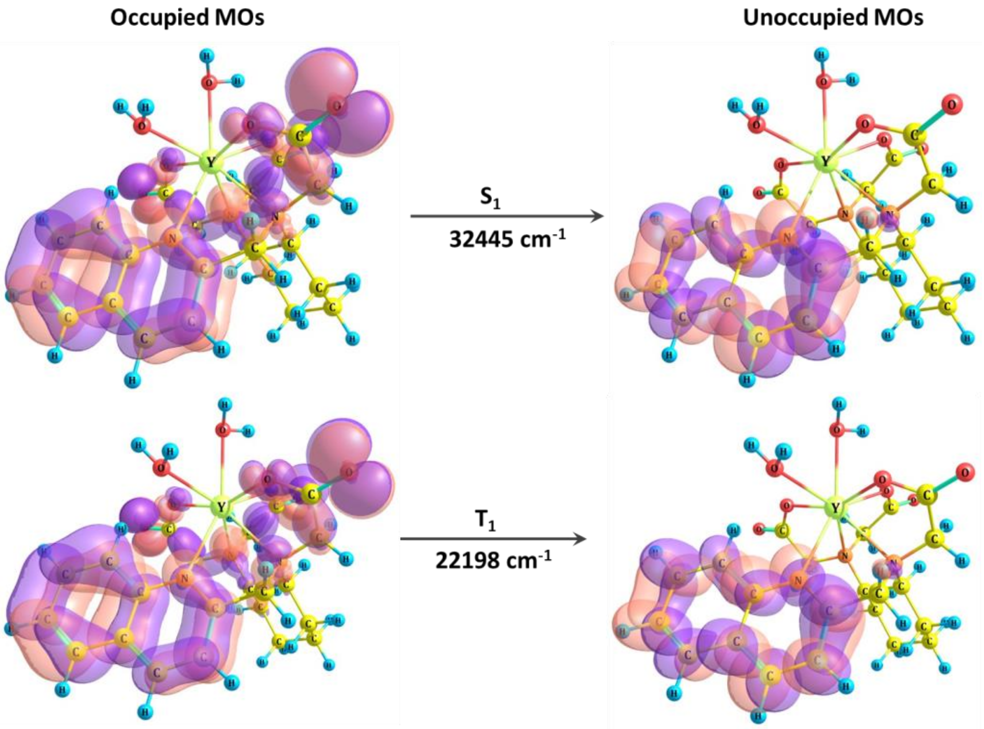

Figure S5. Kohn−Sham molecular orbitals composition of S_1 and T_1 states for *trans*-N,O-[Y(QC3A)(H_2O)$_2$] (**5**).

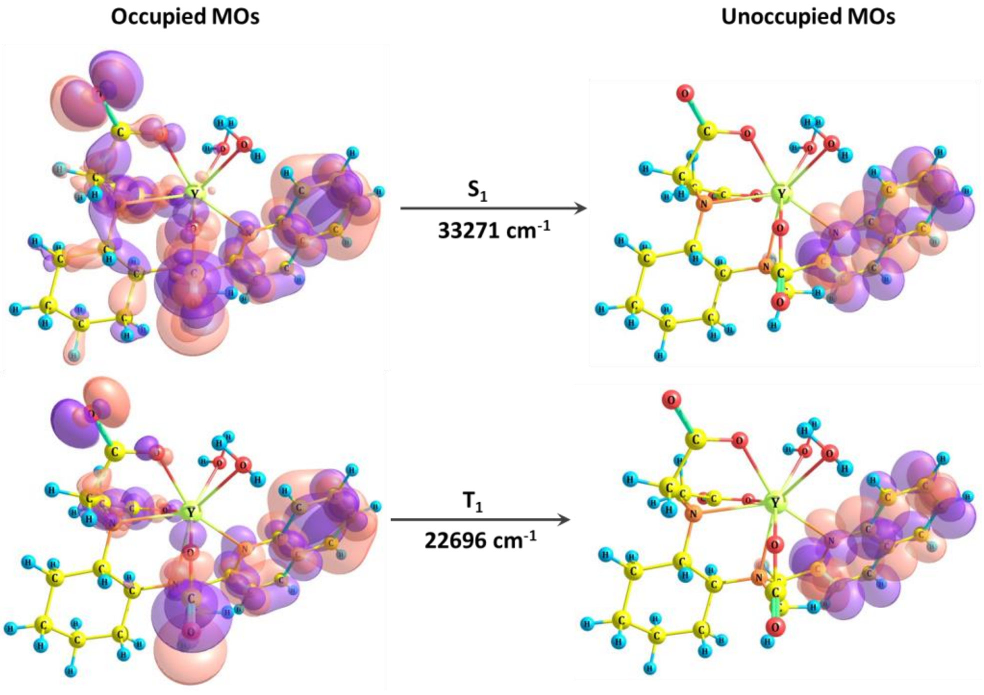

Figure S6. Kohn−Sham molecular orbitals composition of S_1 and T_1 states for *trans*-O,O-[Y(QC3A)(H_2O)$_2$] (**6**).

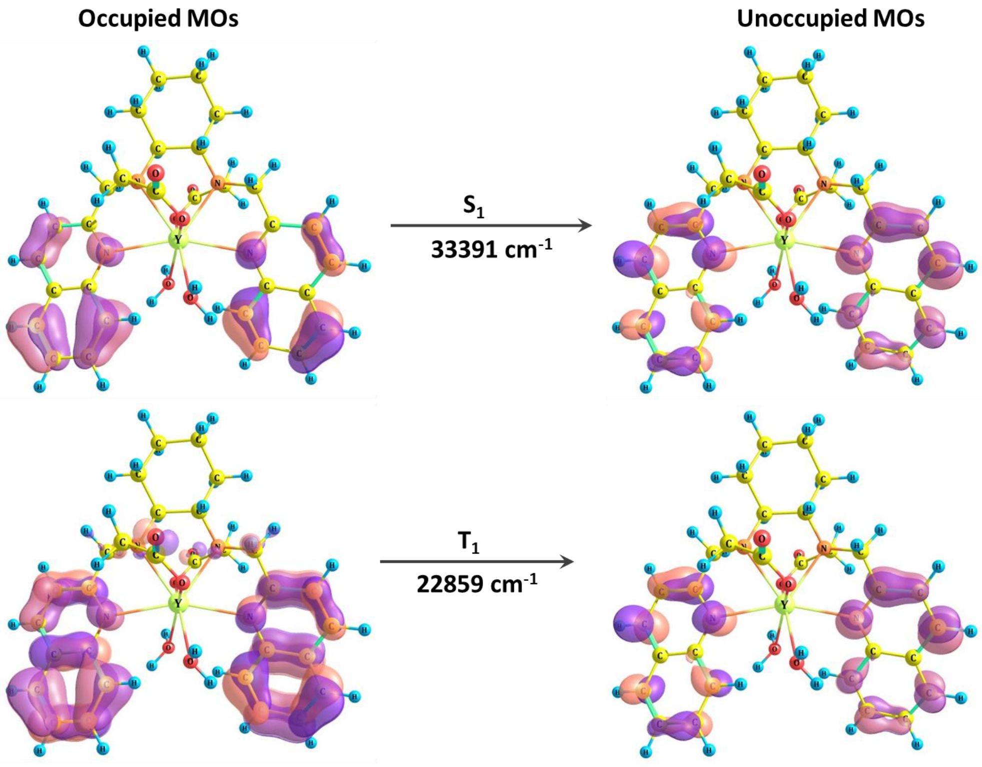

Figure S7. Kohn−Sham molecular orbitals composition of S_1 and T_1 states for *trans*-O,O-[Y(bQcd)(H_2O)$_2$]$^+$ (**7**).

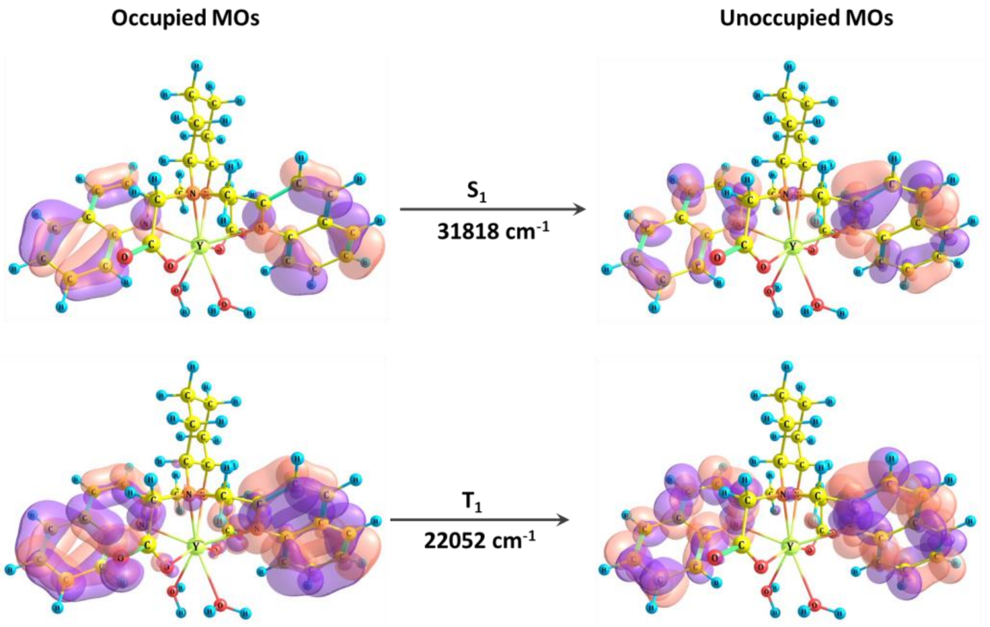

Figure S8. Kohn−Sham molecular orbitals composition of S_1 and T_1 states for *trans*-N,N-[Y(bQcd)(H_2O)$_2$]$^+$ (**8**).

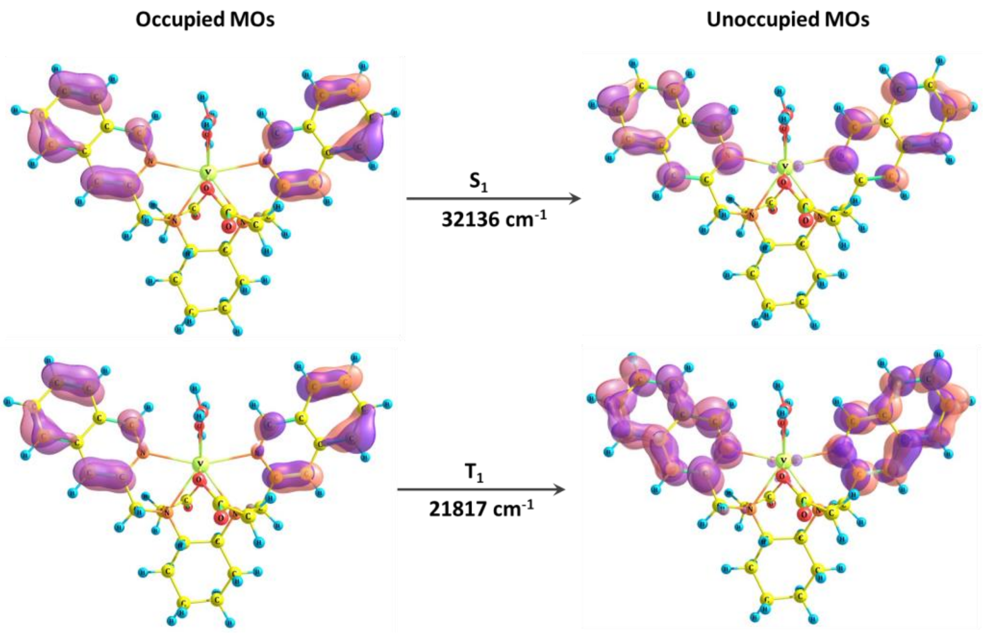

Figure S9. Kohn−Sham molecular orbitals composition of S_1 and T_1 states for *trans*-O,O-$[Y(bisoQcd)(H_2O)_2]^+$ (**9**).

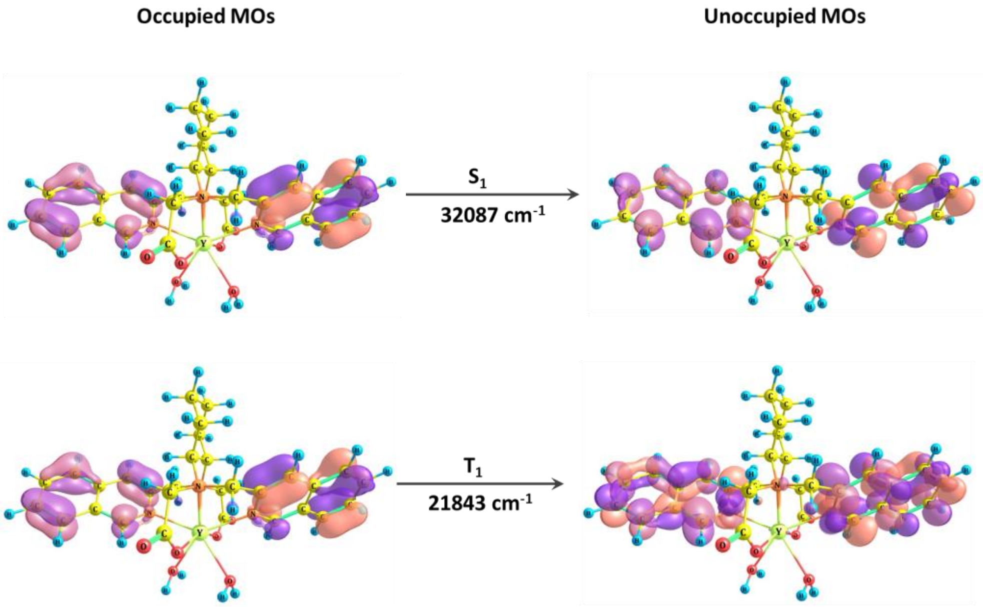

Figure S10. Kohn−Sham molecular orbitals composition of S_1 and T_1 states for *trans*-N,N-$[Y(bisoQcd)(H_2O)_2]^+$ (**10**).

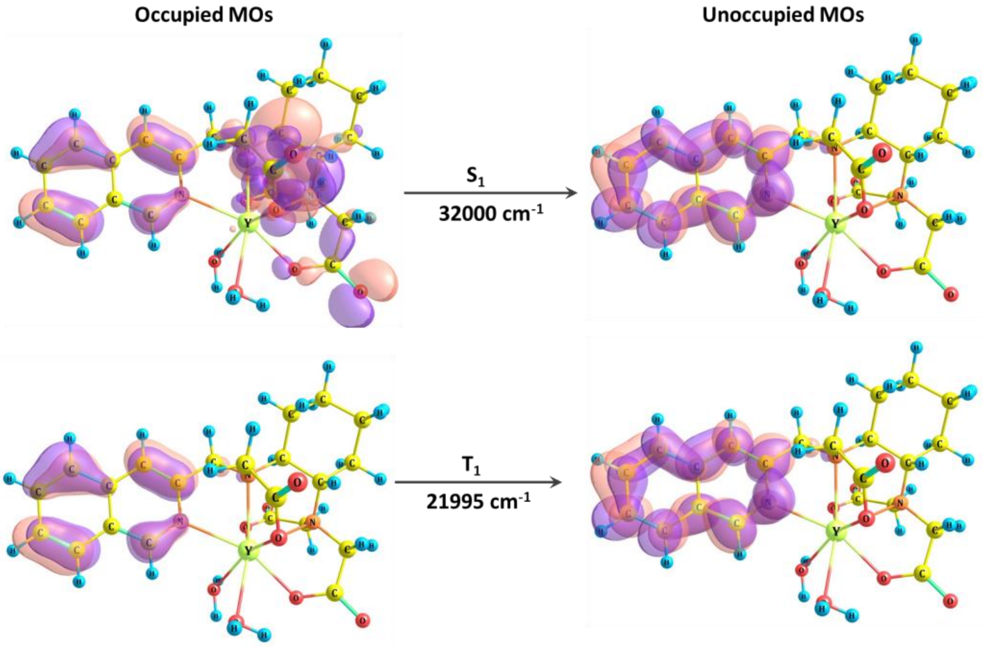

Figure S11. Kohn−Sham molecular orbitals composition of S$_1$ and T$_1$ states for *trans*-O,O-[Y(*iso*QC3A)(H_2O)$_2$] (**11**).

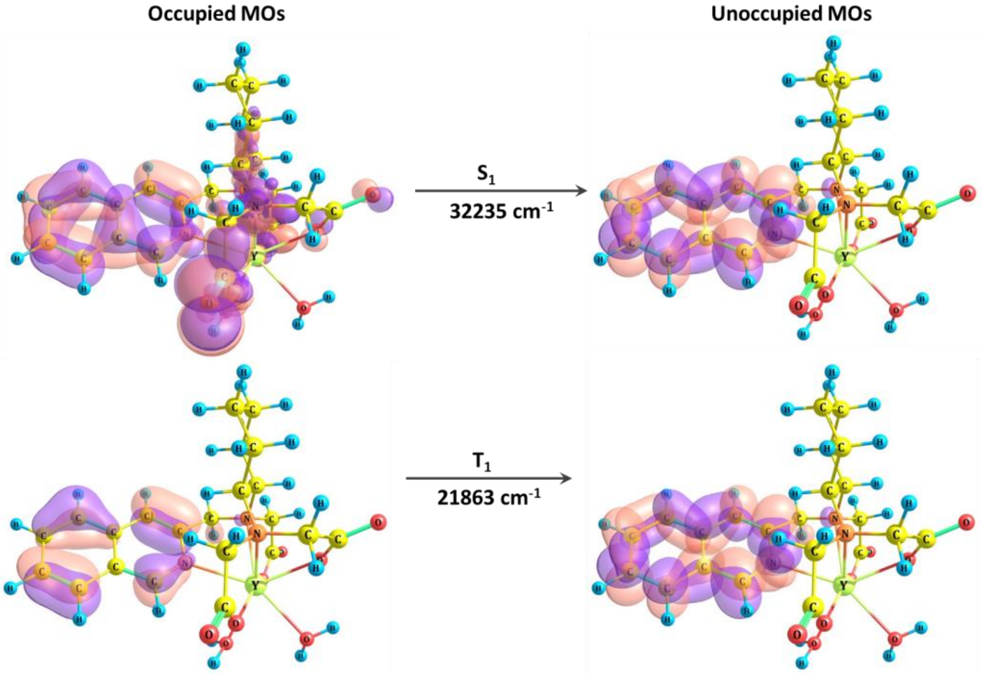

Figure S12. Kohn−Sham molecular orbitals composition of S$_1$ and T$_1$ states for *trans*-N,O-[Y(*iso*QC3A)(H_2O)$_2$] (**12**).

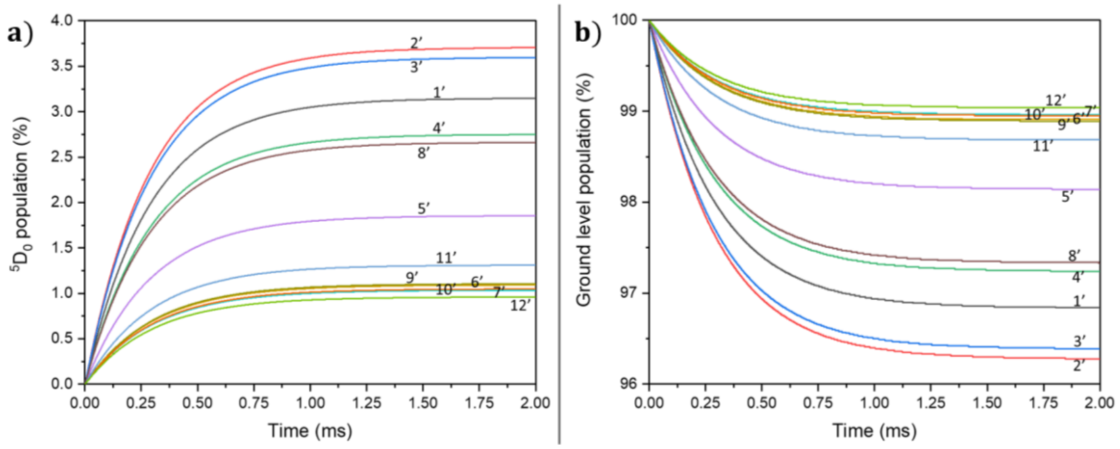

Figure S13. Simulated populations transient of a) $^5D_0$ and b) ground (S_0 and ^7F_J) levels obtained from rate equations model for Eu^{3+}-based complexes studied.

S4. IET rates

Table S3. Data obtained for ***trans*-O,O-[Eu(bpcd)(H_2O)$_2$]$^+$** (**1'**): Δ is the energy difference between donor and acceptor states, $\boldsymbol{W_{d-d}}$, $\boldsymbol{W_{d-m}}$, and $\boldsymbol{W_{ex}}$ stands for the dipole–dipole, dipole–multipole, and exchange energy transfer mechanisms (in s^{-1}), respectively. $\boldsymbol{W}$ is the sum of them in each pathway multiplied by the thermal population of the initial acceptor level (0.64 for $^7F_0$ and 0.33 for $^7F_1$) and by the barrier factor (applied only when Δ is negative).

Pathway Label	Donor	Acceptor	Δ	$\boldsymbol{W_{d-d}}$	$\boldsymbol{W_{d-m}}$	$\boldsymbol{W_{ex}}$	$\boldsymbol{W}$	$\boldsymbol{W_b}$
1	S_1	$^7F_0 \rightarrow {}^5D_0$	22119	4.71E-12	5.14E-08	0.00E+00	1.29E-10	1.09E-56
2	S_1	$^7F_0 \rightarrow {}^5D_1$	20385	0.00E+00	0.00E+00	8.63E-05	5.52E-05	1.92E-47
3	S_1	$^7F_0 \rightarrow {}^5D_2$	17929	6.18E-07	6.74E-03	0.00E+00	4.32E-03	1.96E-40
4	S_1	$^7F_0 \rightarrow {}^5L_6$	14087	2.65E+00	6.01E-02	0.00E+00	1.74E+00	7.92E-30
5	S_1	$^7F_0 \rightarrow {}^5G_6$	12660	1.21E+01	2.75E-01	0.00E+00	7.94E+00	3.40E-26
6	S_1	$^7F_0 \rightarrow {}^5D_4$	11826	6.90E+00	3.01E+01	0.00E+00	2.37E+01	5.52E-24
7	S_1	$^7F_1 \rightarrow {}^5D_0$	22491	0.00E+00	0.00E+00	1.16E-07	3.84E-08	5.48E-55
8	S_1	$^7F_1 \rightarrow {}^5D_1$	20757	1.41E-10	1.54E-06	9.08E-09	5.11E-07	2.98E-50
9	S_1	$^7F_1 \rightarrow {}^5D_2$	18301	0.00E+00	0.00E+00	5.71E-03	1.88E-03	1.44E-41
10	S_1	$^7F_1 \rightarrow {}^5D_3$	15429	1.37E-03	6.98E-01	0.00E+00	2.31E-01	1.69E-33
11	S_1	$^7F_1 \rightarrow {}^5L_6$	14459	2.32E-01	5.26E-03	0.00E+00	7.84E-02	6.00E-32
12	S_1	$^7F_1 \rightarrow {}^5L_7$	13427	4.24E+00	9.60E-02	0.00E+00	1.43E+00	1.55E-28
13	S_1	$^7F_1 \rightarrow {}^5G_2$	13392	0.00E+00	0.00E+00	1.01E+03	3.35E+02	4.28E-26
14	S_1	$^7F_1 \rightarrow {}^5G_3$	13162	1.96E-01	5.18E+01	0.00E+00	1.71E+01	6.61E-27
15	S_1	$^7F_1 \rightarrow {}^5G_6$	13032	2.57E+00	5.81E-02	0.00E+00	8.66E-01	6.22E-28
16	S_1	$^7F_1 \rightarrow {}^5G_5$	13021	5.28E+00	4.88E-01	0.00E+00	1.90E+00	1.44E-27
						$\boldsymbol{W^S}$	**3.90E+02**	
						$\boldsymbol{W_b^S}$		**5.61E-24**
17	T_1	$^7F_0 \rightarrow {}^5D_0$	14142	7.43E-07	8.11E-03	0.00E+00	2.03E-05	7.12E-35
18	T_1	$^7F_0 \rightarrow {}^5D_1$	12408	0.00E+00	0.00E+00	1.62E+04	1.03E+04	1.48E-22
19	T_1	$^7F_0 \rightarrow {}^5D_2$	9952	5.66E-04	6.18E+00	0.00E+00	3.96E+00	7.40E-21
20	T_1	$^7F_0 \rightarrow {}^5L_6$	6110	2.17E+01	4.91E-01	0.00E+00	1.42E+01	2.67E-12
21	T_1	$^7F_0 \rightarrow {}^5G_6$	4683	1.71E+01	3.89E-01	0.00E+00	1.12E+01	1.98E-09
22	T_1	$^7F_0 \rightarrow {}^5D_4$	3849	3.50E+00	1.52E+01	0.00E+00	1.20E+01	1.16E-07
23	T_1	$^7F_1 \rightarrow {}^5D_0$	14514	0.00E+00	0.00E+00	2.90E+02	9.56E+01	5.63E-29
24	T_1	$^7F_1 \rightarrow {}^5D_1$	12780	4.17E-06	4.56E-02	2.69E+00	9.02E-01	2.17E-27
25	T_1	$^7F_1 \rightarrow {}^5D_2$	10324	0.00E+00	0.00E+00	8.26E+04	2.73E+04	8.57E-18
26	T_1	$^7F_1 \rightarrow {}^5D_3$	7452	5.80E-02	2.96E+01	0.00E+00	9.79E+00	2.95E-15
27	T_1	$^7F_1 \rightarrow {}^5L_6$	6482	2.99E+00	6.78E-02	0.00E+00	1.01E+00	3.19E-14
28	T_1	$^7F_1 \rightarrow {}^5L_7$	5450	1.54E+01	3.48E-01	0.00E+00	5.19E+00	2.31E-11
29	T_1	$^7F_1 \rightarrow {}^5G_2$	5415	0.00E+00	0.00E+00	3.52E+07	1.16E+07	6.13E-05
30	T_1	$^7F_1 \rightarrow {}^5G_3$	5185	5.15E-01	1.36E+02	0.00E+00	4.49E+01	7.14E-10
31	T_1	$^7F_1 \rightarrow {}^5G_6$	5055	5.73E+00	1.30E-01	0.00E+00	1.93E+00	5.73E-11
32	T_1	$^7F_1 \rightarrow {}^5G_5$	5044	1.16E+01	1.07E+00	0.00E+00	4.19E+00	1.31E-10
						$\boldsymbol{W^T}$	**1.17E+07**	
						$\boldsymbol{W^{T'}}$	**9.56E+01**	
						$\boldsymbol{W_b^T}$		**6.14E-05**
						$\boldsymbol{W_b^{T'}}$		**5.63E-29**

Table S4. Data obtained for ***trans*-N,N-[Eu(bpcd)(H_2O)$_2$]$^+$ (2')**: Δ is the energy difference between donor and acceptor states, W_{d-d}, W_{d-m}, and W_{ex} stands for the dipole–dipole, dipole–multipole, and exchange energy transfer mechanisms (in s^{-1}), respectively. W is the sum of them in each pathway multiplied by the thermal population of the initial acceptor level (0.64 for $^7F_0$ and 0.33 for $^7F_1$) and by the barrier factor (applied only when Δ is negative).

Pathway Label	Donor	Acceptor	Δ	W_{d-d}	W_{d-m}	W_{ex}	W	W_b
1	S_1	$^7F_0 \to {}^5D_0$	20892	5.42E-10	2.89E-06	0.00E+00	7.23E-09	2.21E-52
2	S_1	$^7F_0 \to {}^5D_1$	19158	0.00E+00	0.00E+00	3.91E-03	2.50E-03	3.12E-43
3	S_1	$^7F_0 \to {}^5D_2$	16702	3.22E-05	1.72E-01	0.00E+00	1.10E-01	1.79E-36
4	S_1	$^7F_0 \to {}^5L_6$	12860	2.19E+01	8.52E-01	0.00E+00	1.46E+01	2.38E-26
5	S_1	$^7F_0 \to {}^5G_6$	11433	7.64E+01	2.98E+00	0.00E+00	5.08E+01	7.80E-23
6	S_1	$^7F_0 \to {}^5D_4$	10599	3.51E+01	2.71E+02	0.00E+00	1.96E+02	1.64E-20
7	S_1	$^7F_1 \to {}^5D_0$	21264	0.00E+00	0.00E+00	7.83E-06	2.59E-06	1.32E-50
8	S_1	$^7F_1 \to {}^5D_1$	19530	1.25E-08	6.70E-05	4.41E-07	2.22E-05	4.66E-46
9	S_1	$^7F_1 \to {}^5D_2$	17074	0.00E+00	0.00E+00	1.74E-01	5.75E-02	1.57E-37
10	S_1	$^7F_1 \to {}^5D_3$	14202	1.52E-02	1.11E+01	0.00E+00	3.67E+00	9.62E-30
11	S_1	$^7F_1 \to {}^5L_6$	13232	2.05E+00	8.00E-02	0.00E+00	7.05E-01	1.94E-28
12	S_1	$^7F_1 \to {}^5L_7$	12200	3.09E+01	1.20E+00	0.00E+00	1.06E+01	4.10E-25
13	S_1	$^7F_1 \to {}^5G_2$	12165	0.00E+00	0.00E+00	1.22E+04	4.04E+03	1.85E-22
14	S_1	$^7F_1 \to {}^5G_3$	11935	1.35E+00	5.37E+02	0.00E+00	1.78E+02	2.46E-23
15	S_1	$^7F_1 \to {}^5G_6$	11805	1.73E+01	6.75E-01	0.00E+00	5.94E+00	1.53E-24
16	S_1	$^7F_1 \to {}^5G_5$	11794	3.56E+01	5.54E+00	0.00E+00	1.36E+01	3.68E-24
						W^S	**4.51E+03**	
						W_b^S		**1.67E-20**
17	T_1	$^7F_0 \to {}^5D_0$	14068	1.73E-06	9.22E-03	0.00E+00	2.31E-05	1.15E-34
18	T_1	$^7F_0 \to {}^5D_1$	12334	0.00E+00	0.00E+00	2.01E+04	1.29E+04	2.63E-22
19	T_1	$^7F_0 \to {}^5D_2$	9878	1.25E-03	6.69E+00	0.00E+00	4.28E+00	1.14E-20
20	T_1	$^7F_0 \to {}^5L_6$	6036	1.50E+01	5.85E-01	0.00E+00	9.98E+00	2.68E-12
21	T_1	$^7F_0 \to {}^5G_6$	4609	1.17E+01	4.55E-01	0.00E+00	7.78E+00	1.96E-09
22	T_1	$^7F_0 \to {}^5D_4$	3775	2.24E+00	1.72E+01	0.00E+00	1.25E+01	1.71E-07
23	T_1	$^7F_1 \to {}^5D_0$	14440	0.00E+00	0.00E+00	3.69E+02	1.22E+02	1.02E-28
24	T_1	$^7F_1 \to {}^5D_1$	12706	9.55E-06	5.10E-02	3.36E+00	1.12E+00	3.86E-27
25	T_1	$^7F_1 \to {}^5D_2$	10250	0.00E+00	0.00E+00	1.00E+05	3.31E+04	1.48E-17
26	T_1	$^7F_1 \to {}^5D_3$	7378	4.26E-02	3.12E+01	0.00E+00	1.03E+01	4.43E-15
27	T_1	$^7F_1 \to {}^5L_6$	6408	2.08E+00	8.11E-02	0.00E+00	7.15E-01	3.22E-14
28	T_1	$^7F_1 \to {}^5L_7$	5376	1.06E+01	4.12E-01	0.00E+00	3.63E+00	2.30E-11
29	T_1	$^7F_1 \to {}^5G_2$	5341	0.00E+00	0.00E+00	4.05E+07	1.34E+07	1.00E-04
30	T_1	$^7F_1 \to {}^5G_3$	5111	3.52E-01	1.39E+02	0.00E+00	4.61E+01	1.04E-09
31	T_1	$^7F_1 \to {}^5G_6$	4981	3.92E+00	1.53E-01	0.00E+00	1.35E+00	5.68E-11
32	T_1	$^7F_1 \to {}^5G_5$	4970	7.95E+00	1.24E+00	0.00E+00	3.03E+00	1.35E-10
						W^T	**1.34E+07**	
						$W^{T'}$	**1.22E+02**	
						W_b^T		**1.01E-04**
						$W_b^{T'}$		**1.02E-28**

Table S5. Data obtained for ***trans*-N,O-[Eu(Pyc3A)(H_2O)$_2$] (3')**: Δ is the energy difference between donor and acceptor states, $\boldsymbol{W_{d-d}}$, $\boldsymbol{W_{d-m}}$, and $\boldsymbol{W_{ex}}$ stands for the dipole–dipole, dipole–multipole, and exchange energy transfer mechanisms (in s^{-1}), respectively. $\boldsymbol{W}$ is the sum of them in each pathway multiplied by the thermal population of the initial acceptor level (0.64 for $^7F_0$ and 0.33 for $^7F_1$) and by the barrier factor (applied only when Δ is negative).

Pathway Label	Donor	Acceptor	Δ	W_{d-d}	W_{d-m}	W_{ex}	W	W_b
1	S_1	$^7F_0 \rightarrow ^5D_0$	21253	3.00E-10	8.78E-07	0.00E+00	2.20E-09	1.19E-53
2	S_1	$^7F_0 \rightarrow ^5D_1$	19519	0.00E+00	0.00E+00	1.17E-03	7.50E-04	1.66E-44
3	S_1	$^7F_0 \rightarrow ^5D_2$	17063	2.25E-05	6.59E-02	0.00E+00	4.22E-02	1.22E-37
4	S_1	$^7F_0 \rightarrow ^5L_6$	13221	1.34E+01	3.44E-01	0.00E+00	8.81E+00	2.56E-27
5	S_1	$^7F_0 \rightarrow ^5G_6$	11794	5.07E+01	1.30E+00	0.00E+00	3.33E+01	9.07E-24
6	S_1	$^7F_0 \rightarrow ^5D_4$	10960	2.76E+01	1.30E+02	0.00E+00	1.01E+02	1.49E-21
7	S_1	$^7F_1 \rightarrow ^5D_0$	21625	0.00E+00	0.00E+00	2.09E-06	6.90E-07	6.27E-52
8	S_1	$^7F_1 \rightarrow ^5D_1$	19891	7.49E-09	2.19E-05	1.30E-07	7.28E-06	2.71E-47
9	S_1	$^7F_1 \rightarrow ^5D_2$	17435	0.00E+00	0.00E+00	5.87E-02	1.94E-02	9.39E-39
10	S_1	$^7F_1 \rightarrow ^5D_3$	14563	1.01E-02	4.88E+00	0.00E+00	1.61E+00	7.51E-31
11	S_1	$^7F_1 \rightarrow ^5L_6$	13593	1.23E+00	3.16E-02	0.00E+00	4.18E-01	2.04E-29
12	S_1	$^7F_1 \rightarrow ^5L_7$	12561	1.96E+01	5.03E-01	0.00E+00	6.64E+00	4.57E-26
13	S_1	$^7F_1 \rightarrow ^5G_2$	12526	0.00E+00	0.00E+00	5.42E+03	1.79E+03	1.45E-23
14	S_1	$^7F_1 \rightarrow ^5G_3$	12296	1.01E+00	2.68E+02	0.00E+00	8.87E+01	2.17E-24
15	S_1	$^7F_1 \rightarrow ^5G_6$	12166	1.13E+01	2.89E-01	0.00E+00	3.82E+00	1.74E-25
16	S_1	$^7F_1 \rightarrow ^5G_5$	12155	2.32E+01	2.46E+00	0.00E+00	8.46E+00	4.08E-25
						$\boldsymbol{W^S}$	**2.04E+03**	
						$\boldsymbol{W_b^S}$		**1.52E-21**
17	T_1	$^7F_0 \rightarrow ^5D_0$	14075	3.04E-06	8.89E-03	0.00E+00	2.22E-05	1.08E-34
18	T_1	$^7F_0 \rightarrow ^5D_1$	12341	0.00E+00	0.00E+00	1.74E+04	1.12E+04	2.21E-22
19	T_1	$^7F_0 \rightarrow ^5D_2$	9885	2.21E-03	6.48E+00	0.00E+00	4.15E+00	1.07E-20
20	T_1	$^7F_0 \rightarrow ^5L_6$	6043	1.89E+01	4.84E-01	0.00E+00	1.24E+01	3.22E-12
21	T_1	$^7F_0 \rightarrow ^5G_6$	4616	1.47E+01	3.77E-01	0.00E+00	9.67E+00	2.35E-09
22	T_1	$^7F_0 \rightarrow ^5D_4$	3782	3.18E+00	1.50E+01	0.00E+00	1.16E+01	1.54E-07
23	T_1	$^7F_1 \rightarrow ^5D_0$	14447	0.00E+00	0.00E+00	3.19E+02	1.05E+02	8.56E-29
24	T_1	$^7F_1 \rightarrow ^5D_1$	12713	1.68E-05	4.92E-02	2.91E+00	9.76E-01	3.25E-27
25	T_1	$^7F_1 \rightarrow ^5D_2$	10257	0.00E+00	0.00E+00	8.72E+04	2.88E+04	1.25E-17
26	T_1	$^7F_1 \rightarrow ^5D_3$	7385	6.25E-02	3.03E+01	0.00E+00	1.00E+01	4.16E-15
27	T_1	$^7F_1 \rightarrow ^5L_6$	6415	2.62E+00	6.71E-02	0.00E+00	8.87E-01	3.86E-14
28	T_1	$^7F_1 \rightarrow ^5L_7$	5383	1.33E+01	3.41E-01	0.00E+00	4.50E+00	2.77E-11
29	T_1	$^7F_1 \rightarrow ^5G_2$	5348	0.00E+00	0.00E+00	3.53E+07	1.17E+07	8.48E-05
30	T_1	$^7F_1 \rightarrow ^5G_3$	5118	5.09E-01	1.35E+02	0.00E+00	4.49E+01	9.83E-10
31	T_1	$^7F_1 \rightarrow ^5G_6$	4988	4.94E+00	1.26E-01	0.00E+00	1.67E+00	6.83E-11
32	T_1	$^7F_1 \rightarrow ^5G_5$	4977	1.00E+01	1.06E+00	0.00E+00	3.66E+00	1.58E-10
						$\boldsymbol{W^T}$	**1.17E+07**	
						$\boldsymbol{W^{T'}}$	**1.05E+02**	
						$\boldsymbol{W_b^T}$		**8.50E-05**
						$\boldsymbol{W_b^{T'}}$		**8.56E-29**

Table S6. Data obtained for ***trans*-O,O-[Eu(Pyc3A)(H_2O)$_2$] (4')**: Δ is the energy difference between donor and acceptor states, W_{d-d}, W_{d-m}, and W_{ex} stands for the dipole–dipole, dipole–multipole, and exchange energy transfer mechanisms (in s^{-1}), respectively. W is the sum of them in each pathway multiplied by the thermal population of the initial acceptor level (0.64 for $^7F_0$ and 0.33 for $^7F_1$) and by the barrier factor (applied only when Δ is negative).

Pathway Label	Donor	Acceptor	Δ	W_{d-d}	W_{d-m}	W_{ex}	W	W_b
1	S_1	$^7F_0 \to ^5D_0$	22525	3.52E-12	1.14E-08	0.00E+00	2.86E-11	3.46E-58
2	S_1	$^7F_0 \to ^5D_1$	20791	0.00E+00	0.00E+00	2.24E-05	1.43E-05	7.09E-49
3	S_1	$^7F_0 \to ^5D_2$	18335	5.99E-07	1.95E-03	0.00E+00	1.25E-03	8.07E-42
4	S_1	$^7F_0 \to ^5L_6$	14493	1.03E+00	2.24E-02	0.00E+00	6.77E-01	4.39E-31
5	S_1	$^7F_0 \to ^5G_6$	13066	5.17E+00	1.12E-01	0.00E+00	3.38E+00	2.06E-27
6	S_1	$^7F_0 \to ^5D_4$	12232	3.24E+00	1.32E+01	0.00E+00	1.05E+01	3.50E-25
7	S_1	$^7F_1 \to ^5D_0$	22897	0.00E+00	0.00E+00	2.64E-08	8.72E-09	1.77E-56
8	S_1	$^7F_1 \to ^5D_1$	21163	1.15E-10	3.73E-07	2.30E-09	1.24E-07	1.03E-51
9	S_1	$^7F_1 \to ^5D_2$	18707	0.00E+00	0.00E+00	1.69E-03	5.56E-04	6.04E-43
10	S_1	$^7F_1 \to ^5D_3$	15835	5.61E-04	2.36E-01	0.00E+00	7.81E-02	8.13E-35
11	S_1	$^7F_1 \to ^5L_6$	14865	8.85E-02	1.91E-03	0.00E+00	2.98E-02	3.25E-33
12	S_1	$^7F_1 \to ^5L_7$	13833	1.72E+00	3.73E-02	0.00E+00	5.81E-01	8.93E-30
13	S_1	$^7F_1 \to ^5G_2$	13798	0.00E+00	0.00E+00	4.07E+02	1.34E+02	2.45E-27
14	S_1	$^7F_1 \to ^5G_3$	13568	8.91E-02	2.02E+01	0.00E+00	6.69E+00	3.67E-28
15	S_1	$^7F_1 \to ^5G_6$	13438	1.07E+00	2.31E-02	0.00E+00	3.60E-01	3.69E-29
16	S_1	$^7F_1 \to ^5G_5$	13427	2.20E+00	1.98E-01	0.00E+00	7.92E-01	8.54E-29
						W^S	**1.58E+02**	
						W_b^S		**3.55E-25**
17	T_1	$^7F_0 \to ^5D_0$	14122	2.34E-06	7.62E-03	0.00E+00	1.91E-05	7.35E-35
18	T_1	$^7F_0 \to ^5D_1$	12388	0.00E+00	0.00E+00	1.58E+04	1.01E+04	1.59E-22
19	T_1	$^7F_0 \to ^5D_2$	9932	1.76E-03	5.73E+00	0.00E+00	3.67E+00	7.55E-21
20	T_1	$^7F_0 \to ^5L_6$	6090	2.10E+01	4.56E-01	0.00E+00	1.38E+01	2.85E-12
21	T_1	$^7F_0 \to ^5G_6$	4663	1.66E+01	3.59E-01	0.00E+00	1.09E+01	2.11E-09
22	T_1	$^7F_0 \to ^5D_4$	3829	3.54E+00	1.44E+01	0.00E+00	1.15E+01	1.21E-07
23	T_1	$^7F_1 \to ^5D_0$	14494	0.00E+00	0.00E+00	2.85E+02	9.40E+01	6.09E-29
24	T_1	$^7F_1 \to ^5D_1$	12760	1.31E-05	4.26E-02	2.63E+00	8.82E-01	2.34E-27
25	T_1	$^7F_1 \to ^5D_2$	10304	0.00E+00	0.00E+00	8.02E+04	2.65E+04	9.15E-18
26	T_1	$^7F_1 \to ^5D_3$	7432	6.48E-02	2.73E+01	0.00E+00	9.02E+00	2.99E-15
27	T_1	$^7F_1 \to ^5L_6$	6462	2.91E+00	6.30E-02	0.00E+00	9.82E-01	3.41E-14
28	T_1	$^7F_1 \to ^5L_7$	5430	1.49E+01	3.23E-01	0.00E+00	5.03E+00	2.46E-11
29	T_1	$^7F_1 \to ^5G_2$	5395	0.00E+00	0.00E+00	3.37E+07	1.11E+07	6.45E-05
30	T_1	$^7F_1 \to ^5G_3$	5165	5.47E-01	1.24E+02	0.00E+00	4.11E+01	7.18E-10
31	T_1	$^7F_1 \to ^5G_6$	5035	5.55E+00	1.20E-01	0.00E+00	1.87E+00	6.10E-11
32	T_1	$^7F_1 \to ^5G_5$	5024	1.13E+01	1.01E+00	0.00E+00	4.05E+00	1.39E-10
						W^T	**1.12E+07**	
						$W^{T'}$	**9.40E+01**	
						W_b^T		**6.46E-05**
						$W_b^{T'}$		**6.09E-29**

Table S7. Data obtained for ***trans*-N,O-[Eu(QC3A)(H_2O)$_2$] (5')**: Δ is the energy difference between donor and acceptor states, W_{d-d}, W_{d-m}, and W_{ex} stands for the dipole–dipole, dipole–multipole, and exchange energy transfer mechanisms (in s^{-1}), respectively. W is the sum of them in each pathway multiplied by the thermal population of the initial acceptor level (0.64 for $^7F_0$ and 0.33 for $^7F_1$) and by the barrier factor (applied only when Δ is negative).

Pathway Label	Donor	Acceptor	Δ	W_{d-d}	W_{d-m}	W_{ex}	W	W_b
1	S_1	$^7F_0 \rightarrow {}^5D_0$	15152	6.00E-03	1.03E+01	0.00E+00	2.58E-02	7.11E-34
2	S_1	$^7F_0 \rightarrow {}^5D_1$	13418	0.00E+00	0.00E+00	2.12E+03	1.36E+03	1.53E-25
3	S_1	$^7F_0 \rightarrow {}^5D_2$	10962	8.78E+00	1.51E+04	0.00E+00	9.66E+03	1.42E-19
4	S_1	$^7F_0 \rightarrow {}^5L_6$	7120	1.05E+05	1.02E+03	0.00E+00	6.80E+04	1.00E-10
5	S_1	$^7F_0 \rightarrow {}^5G_6$	5693	1.04E+05	1.01E+03	0.00E+00	6.72E+04	9.31E-08
6	S_1	$^7F_0 \rightarrow {}^5D_4$	4859	2.54E+04	6.81E+04	0.00E+00	5.99E+04	4.53E-06
7	S_1	$^7F_1 \rightarrow {}^5D_0$	15524	0.00E+00	0.00E+00	2.73E+01	9.02E+00	4.17E-32
8	S_1	$^7F_1 \rightarrow {}^5D_1$	13790	4.17E-02	7.16E+01	3.32E-01	2.38E+01	4.49E-28
9	S_1	$^7F_1 \rightarrow {}^5D_2$	11334	0.00E+00	0.00E+00	1.50E+04	4.94E+03	1.22E-20
10	S_1	$^7F_1 \rightarrow {}^5D_3$	8462	2.91E+02	1.06E+05	0.00E+00	3.52E+04	8.34E-14
11	S_1	$^7F_1 \rightarrow {}^5L_6$	7492	1.37E+04	1.34E+02	0.00E+00	4.57E+03	1.13E-12
12	S_1	$^7F_1 \rightarrow {}^5L_7$	6460	8.27E+04	8.06E+02	0.00E+00	2.76E+04	9.64E-10
13	S_1	$^7F_1 \rightarrow {}^5G_2$	6425	0.00E+00	0.00E+00	1.37E+07	4.52E+06	1.87E-07
14	S_1	$^7F_1 \rightarrow {}^5G_3$	6195	3.36E+03	6.92E+05	0.00E+00	2.29E+05	2.86E-08
15	S_1	$^7F_1 \rightarrow {}^5G_6$	6065	3.28E+04	3.19E+02	0.00E+00	1.09E+04	2.54E-09
16	S_1	$^7F_1 \rightarrow {}^5G_5$	6054	6.67E+04	3.66E+03	0.00E+00	2.32E+04	5.69E-09
						W^S	**5.07E+06**	
						W_b^S		**4.85E-06**
17	T_1	$^7F_0 \rightarrow {}^5D_0$	4905	1.50E+00	2.58E+03	0.00E+00	6.45E+00	3.93E-10
18	T_1	$^7F_0 \rightarrow {}^5D_1$	3171	0.00E+00	0.00E+00	3.43E+08	2.19E+08	5.46E+01
19	T_1	$^7F_0 \rightarrow {}^5D_2$	715	2.94E+00	5.06E+03	0.00E+00	3.24E+03	1.05E+02
20	T_1	$^7F_0 \rightarrow {}^5L_6$	-3127	8.19E+01	7.98E-01	0.00E+00	1.62E-05	5.29E+01
21	T_1	$^7F_0 \rightarrow {}^5G_6$	-4554	8.52E+00	8.30E-02	0.00E+00	1.80E-09	5.50E+00
22	T_1	$^7F_0 \rightarrow {}^5D_4$	-5388	5.58E-01	1.50E+00	0.00E+00	7.86E-12	1.31E+00
23	T_1	$^7F_1 \rightarrow {}^5D_0$	5277	0.00E+00	0.00E+00	1.23E+08	4.06E+07	4.15E-04
24	T_1	$^7F_1 \rightarrow {}^5D_1$	3543	1.21E+00	2.08E+03	9.68E+04	3.26E+04	1.36E-03
25	T_1	$^7F_1 \rightarrow {}^5D_2$	1087	0.00E+00	0.00E+00	9.03E+07	2.98E+07	1.63E+05
26	T_1	$^7F_1 \rightarrow {}^5D_3$	-1785	1.88E+00	6.90E+02	0.00E+00	4.36E-02	2.28E+02
27	T_1	$^7F_1 \rightarrow {}^5L_6$	-2755	1.92E+01	1.87E-01	0.00E+00	1.17E-05	6.41E+00
28	T_1	$^7F_1 \rightarrow {}^5L_7$	-3787	2.27E+01	2.22E-01	0.00E+00	9.79E-08	7.57E+00
29	T_1	$^7F_1 \rightarrow {}^5G_2$	-3822	0.00E+00	0.00E+00	3.57E+07	1.29E-01	1.18E+07
30	T_1	$^7F_1 \rightarrow {}^5G_3$	-4052	6.08E-01	1.25E+02	0.00E+00	1.51E-07	4.15E+01
31	T_1	$^7F_1 \rightarrow {}^5G_6$	-4182	4.83E+00	4.71E-02	0.00E+00	3.13E-09	1.61E+00
32	T_1	$^7F_1 \rightarrow {}^5G_5$	-4193	9.66E+00	5.30E-01	0.00E+00	6.20E-09	3.36E+00
						W^T	**2.49E+08**	
						$W^{T'}$	**4.06E+07**	
						W_b^T		**1.19E+07**
						$W_b^{T'}$		**4.15E-04**

Table S8. Data obtained for ***trans*-O,O-[Eu(QC3A)(H_2O)$_2$] (6')**: Δ is the energy difference between donor and acceptor states, $\boldsymbol{W_{d-d}}$, $\boldsymbol{W_{d-m}}$, and $\boldsymbol{W_{ex}}$ stands for the dipole–dipole, dipole–multipole, and exchange energy transfer mechanisms (in s^{-1}), respectively. $\boldsymbol{W}$ is the sum of them in each pathway multiplied by the thermal population of the initial acceptor level (0.64 for $^7F_0$ and 0.33 for $^7F_1$) and by the barrier factor (applied only when Δ is negative).

**Pathway Label**	**Donor**	**Acceptor**	**Δ**	$\boldsymbol{W_{d-d}}$	$\boldsymbol{W_{d-m}}$	$\boldsymbol{W_{ex}}$	$\boldsymbol{W}$	$\boldsymbol{W_b}$
1	S_1	$^7F_0 \rightarrow ^5D_0$	15978	8.94E-04	1.48E+00	0.00E+00	3.70E-03	1.94E-36
2	S_1	$^7F_0 \rightarrow ^5D_1$	14244	0.00E+00	0.00E+00	3.79E+02	2.43E+02	5.20E-28
3	S_1	$^7F_0 \rightarrow ^5D_2$	11788	2.23E+00	3.68E+03	0.00E+00	2.36E+03	6.60E-22
4	S_1	$^7F_0 \rightarrow ^5L_6$	7946	4.74E+04	4.16E+02	0.00E+00	3.06E+04	8.62E-13
5	S_1	$^7F_0 \rightarrow ^5G_6$	6519	5.62E+04	4.93E+02	0.00E+00	3.63E+04	9.58E-10
6	S_1	$^7F_0 \rightarrow ^5D_4$	5685	1.49E+04	3.64E+04	0.00E+00	3.28E+04	4.73E-08
7	S_1	$^7F_1 \rightarrow ^5D_0$	16350	0.00E+00	0.00E+00	3.74E+00	1.24E+00	1.09E-34
8	S_1	$^7F_1 \rightarrow ^5D_1$	14616	7.38E-03	1.22E+01	5.67E-02	4.05E+00	1.46E-30
9	S_1	$^7F_1 \rightarrow ^5D_2$	12160	0.00E+00	0.00E+00	3.49E+03	1.15E+03	5.42E-23
10	S_1	$^7F_1 \rightarrow ^5D_3$	9288	1.06E+02	3.57E+04	0.00E+00	1.18E+04	5.33E-16
11	S_1	$^7F_1 \rightarrow ^5L_6$	8318	5.90E+03	5.18E+01	0.00E+00	1.96E+03	9.28E-15
12	S_1	$^7F_1 \rightarrow ^5L_7$	7286	4.06E+04	3.56E+02	0.00E+00	1.35E+04	9.00E-12
13	S_1	$^7F_1 \rightarrow ^5G_2$	7251	0.00E+00	0.00E+00	5.97E+06	1.97E+06	1.55E-09
14	S_1	$^7F_1 \rightarrow ^5G_3$	7021	1.65E+03	3.10E+05	0.00E+00	1.03E+05	2.44E-10
15	S_1	$^7F_1 \rightarrow ^5G_6$	6891	1.69E+04	1.48E+02	0.00E+00	5.63E+03	2.49E-11
16	S_1	$^7F_1 \rightarrow ^5G_5$	6880	3.44E+04	1.68E+03	0.00E+00	1.19E+04	5.57E-11
						$\boldsymbol{W^S}$	**2.22E+06**	
						$\boldsymbol{W_b^S}$		**5.01E-08**
17	T_1	$^7F_0 \rightarrow ^5D_0$	5403	1.09E+00	1.80E+03	0.00E+00	4.51E+00	2.52E-11
18	T_1	$^7F_0 \rightarrow ^5D_1$	3669	0.00E+00	0.00E+00	2.74E+08	1.75E+08	4.00E+00
19	T_1	$^7F_0 \rightarrow ^5D_2$	1213	2.95E+00	4.88E+03	0.00E+00	3.12E+03	9.30E+00
20	T_1	$^7F_0 \rightarrow ^5L_6$	-2629	1.20E+02	1.05E+00	0.00E+00	2.59E-04	7.76E+01
21	T_1	$^7F_0 \rightarrow ^5G_6$	-4056	1.39E+01	1.22E-01	0.00E+00	3.20E-08	9.00E+00
22	T_1	$^7F_0 \rightarrow ^5D_4$	-4890	9.49E-01	2.32E+00	0.00E+00	1.36E-10	2.09E+00
23	T_1	$^7F_1 \rightarrow ^5D_0$	5775	0.00E+00	0.00E+00	8.36E+07	2.76E+07	2.59E-05
24	T_1	$^7F_1 \rightarrow ^5D_1$	4041	9.78E-01	1.62E+03	7.52E+04	2.53E+04	9.72E-05
25	T_1	$^7F_1 \rightarrow ^5D_2$	1585	0.00E+00	0.00E+00	8.47E+07	2.80E+07	1.40E+04
26	T_1	$^7F_1 \rightarrow ^5D_3$	-1287	2.40E+00	8.06E+02	0.00E+00	5.56E-01	2.67E+02
27	T_1	$^7F_1 \rightarrow ^5L_6$	-2257	2.74E+01	2.40E-01	0.00E+00	1.81E-04	9.12E+00
28	T_1	$^7F_1 \rightarrow ^5L_7$	-3289	3.51E+01	3.08E-01	0.00E+00	1.65E-06	1.17E+01
29	T_1	$^7F_1 \rightarrow ^5G_2$	-3324	0.00E+00	0.00E+00	4.88E+07	1.92E+00	1.61E+07
30	T_1	$^7F_1 \rightarrow ^5G_3$	-3554	9.27E-01	1.74E+02	0.00E+00	2.28E-06	5.77E+01
31	T_1	$^7F_1 \rightarrow ^5G_6$	-3684	7.68E+00	6.74E-02	0.00E+00	5.42E-08	2.56E+00
32	T_1	$^7F_1 \rightarrow ^5G_5$	-3695	1.54E+01	7.51E-01	0.00E+00	1.07E-07	5.32E+00
						$\boldsymbol{W^T}$	**2.03E+08**	
						$\boldsymbol{W^{T'}}$	**2.76E+07**	
						$\boldsymbol{W_b^T}$		**1.61E+07**
						$\boldsymbol{W_b^{T'}}$		**2.59E-05**

Table S9. Data obtained for ***trans*-O,O-[Eu(bQcd)(H₂O)₂]⁺ (7')**: Δ is the energy difference between donor and acceptor states, $\boldsymbol{W_{d-d}}$, $\boldsymbol{W_{d-m}}$, and $\boldsymbol{W_{ex}}$ stands for the dipole–dipole, dipole–multipole, and exchange energy transfer mechanisms (in s^{-1}), respectively. $\boldsymbol{W}$ is the sum of them in each pathway multiplied by the thermal population of the initial acceptor level (0.64 for $^7F_0$ and 0.33 for $^7F_1$) and by the barrier factor (applied only when Δ is negative).

Pathway Label	Donor	Acceptor	Δ	W_{d-d}	W_{d-m}	W_{ex}	W	W_b
1	S_1	$^7F_0 \rightarrow ^5D_0$	16098	2.19E-04	1.13E+00	0.00E+00	2.81E-03	8.30E-37
2	S_1	$^7F_0 \rightarrow ^5D_1$	14364	0.00E+00	0.00E+00	2.87E+02	1.84E+02	2.22E-28
3	S_1	$^7F_0 \rightarrow ^5D_2$	11908	5.90E-01	3.03E+03	0.00E+00	1.94E+03	3.05E-22
4	S_1	$^7F_0 \rightarrow ^5L_6$	8066	3.67E+04	3.53E+02	0.00E+00	2.37E+04	3.76E-13
5	S_1	$^7F_0 \rightarrow ^5G_6$	6639	4.47E+04	4.29E+02	0.00E+00	2.89E+04	4.29E-10
6	S_1	$^7F_0 \rightarrow ^5D_4$	5805	1.16E+04	3.23E+04	0.00E+00	2.81E+04	2.28E-08
7	S_1	$^7F_1 \rightarrow ^5D_0$	16470	0.00E+00	0.00E+00	2.73E+00	8.99E-01	4.46E-35
8	S_1	$^7F_1 \rightarrow ^5D_1$	14736	1.85E-03	9.52E+00	4.26E-02	3.16E+00	6.40E-31
9	S_1	$^7F_1 \rightarrow ^5D_2$	12280	0.00E+00	0.00E+00	2.75E+03	9.07E+02	2.40E-23
10	S_1	$^7F_1 \rightarrow ^5D_3$	9408	6.81E+01	3.08E+04	0.00E+00	1.02E+04	2.58E-16
11	S_1	$^7F_1 \rightarrow ^5L_6$	8438	4.54E+03	4.36E+01	0.00E+00	1.51E+03	4.02E-15
12	S_1	$^7F_1 \rightarrow ^5L_7$	7406	3.18E+04	3.05E+02	0.00E+00	1.06E+04	3.97E-12
13	S_1	$^7F_1 \rightarrow ^5G_2$	7371	0.00E+00	0.00E+00	5.14E+06	1.70E+06	7.53E-10
14	S_1	$^7F_1 \rightarrow ^5G_3$	7141	1.17E+03	2.78E+05	0.00E+00	9.22E+04	1.23E-10
15	S_1	$^7F_1 \rightarrow ^5G_6$	7011	1.34E+04	1.28E+02	0.00E+00	4.45E+03	1.11E-11
16	S_1	$^7F_1 \rightarrow ^5G_5$	7000	2.72E+04	1.46E+03	0.00E+00	9.45E+03	2.48E-11
						W^S	1.91E+06	
						W_b^S		2.41E-08
17	T_1	$^7F_0 \rightarrow ^5D_0$	5566	3.13E-01	1.61E+03	0.00E+00	4.02E+00	1.02E-11
18	T_1	$^7F_0 \rightarrow ^5D_1$	3832	0.00E+00	0.00E+00	2.46E+08	1.57E+08	1.64E+00
19	T_1	$^7F_0 \rightarrow ^5D_2$	1376	9.40E-01	4.83E+03	0.00E+00	3.09E+03	4.20E+00
20	T_1	$^7F_0 \rightarrow ^5L_6$	-2466	1.15E+02	1.10E+00	0.00E+00	5.44E-04	7.43E+01
21	T_1	$^7F_0 \rightarrow ^5G_6$	-3893	1.38E+01	1.33E-01	0.00E+00	6.97E-08	8.94E+00
22	T_1	$^7F_0 \rightarrow ^5D_4$	-4727	9.31E-01	2.58E+00	0.00E+00	3.21E-10	2.25E+00
23	T_1	$^7F_1 \rightarrow ^5D_0$	5938	0.00E+00	0.00E+00	7.12E+07	2.35E+07	1.01E-05
24	T_1	$^7F_1 \rightarrow ^5D_1$	4204	2.91E-01	1.49E+03	6.68E+04	2.25E+04	3.95E-05
25	T_1	$^7F_1 \rightarrow ^5D_2$	1748	0.00E+00	0.00E+00	8.01E+07	2.64E+07	6.04E+03
26	T_1	$^7F_1 \rightarrow ^5D_3$	-1124	1.88E+00	8.50E+02	0.00E+00	1.28E+00	2.81E+02
27	T_1	$^7F_1 \rightarrow ^5L_6$	-2094	2.60E+01	2.49E-01	0.00E+00	3.77E-04	8.66E+00
28	T_1	$^7F_1 \rightarrow ^5L_7$	-3126	3.41E+01	3.28E-01	0.00E+00	3.51E-06	1.14E+01
29	T_1	$^7F_1 \rightarrow ^5G_2$	-3161	0.00E+00	0.00E+00	5.22E+07	4.49E+00	1.72E+07
30	T_1	$^7F_1 \rightarrow ^5G_3$	-3391	8.18E-01	1.94E+02	0.00E+00	5.58E-06	6.44E+01
31	T_1	$^7F_1 \rightarrow ^5G_6$	-3521	7.55E+00	7.25E-02	0.00E+00	1.17E-07	2.52E+00
32	T_1	$^7F_1 \rightarrow ^5G_5$	-3532	1.51E+01	8.11E-01	0.00E+00	2.31E-07	5.25E+00
						W^T	**1.84E+08**	
						$W^{T'}$	**2.35E+07**	
						W_b^T		**1.72E+07**
						$W_b^{T'}$		**1.01E-05**

Table S10. Data obtained for ***trans*-N,N-[Eu(bQcd)(H$_2$O)$_2$]$^+$ (8')**: Δ is the energy difference between donor and acceptor states, W_{d-d}, W_{d-m}, and W_{ex} stands for the dipole–dipole, dipole–multipole, and exchange energy transfer mechanisms (in s^{-1}), respectively. W is the sum of them in each pathway multiplied by the thermal population of the initial acceptor level (0.64 for $^7F_0$ and 0.33 for $^7F_1$) and by the barrier factor (applied only when Δ is negative).

Pathway Label	Donor	Acceptor	Δ	W_{d-d}	W_{d-m}	W_{ex}	W	W_b
1	S_1	$^7F_0 \rightarrow ^5D_0$	14525	1.03E-02	4.79E+01	0.00E+00	1.20E-01	6.70E-32
2	S_1	$^7F_0 \rightarrow ^5D_1$	12791	0.00E+00	0.00E+00	8.59E+03	5.50E+03	1.26E-23
3	S_1	$^7F_0 \rightarrow ^5D_2$	10335	1.00E+01	4.67E+04	0.00E+00	2.99E+04	8.92E-18
4	S_1	$^7F_0 \rightarrow ^5L_6$	6493	1.82E+05	2.40E+03	0.00E+00	1.18E+05	3.53E-09
5	S_1	$^7F_0 \rightarrow ^5G_6$	5066	1.56E+05	2.07E+03	0.00E+00	1.01E+05	2.85E-06
6	S_1	$^7F_0 \rightarrow ^5D_4$	4232	2.84E+04	1.22E+05	0.00E+00	9.64E+04	1.48E-04
7	S_1	$^7F_1 \rightarrow ^5D_0$	14897	0.00E+00	0.00E+00	1.36E+02	4.48E+01	4.21E-30
8	S_1	$^7F_1 \rightarrow ^5D_1$	13163	6.24E-02	2.92E+02	1.40E+00	9.67E+01	3.71E-26
9	S_1	$^7F_1 \rightarrow ^5D_2$	10707	0.00E+00	0.00E+00	4.96E+04	1.64E+04	8.20E-19
10	S_1	$^7F_1 \rightarrow ^5D_3$	7835	4.17E+02	2.59E+05	0.00E+00	8.56E+04	4.11E-12
11	S_1	$^7F_1 \rightarrow ^5L_6$	6865	2.46E+04	3.25E+02	0.00E+00	8.21E+03	4.13E-11
12	S_1	$^7F_1 \rightarrow ^5L_7$	5833	1.34E+05	1.77E+03	0.00E+00	4.48E+04	3.18E-08
13	S_1	$^7F_1 \rightarrow ^5G_2$	5798	0.00E+00	0.00E+00	2.83E+07	9.33E+06	7.83E-06
14	S_1	$^7F_1 \rightarrow ^5G_3$	5568	4.05E+03	1.35E+06	0.00E+00	4.48E+05	1.13E-06
15	S_1	$^7F_1 \rightarrow ^5G_6$	5438	5.11E+04	6.76E+02	0.00E+00	1.71E+04	8.07E-08
16	S_1	$^7F_1 \rightarrow ^5G_5$	5427	1.04E+05	7.44E+03	0.00E+00	3.66E+04	1.82E-07
						W^S	**1.03E+07**	
						W_b^S		**1.60E-04**
17	T_1	$^7F_0 \rightarrow ^5D_0$	4759	6.80E-01	3.18E+03	0.00E+00	7.95E+00	9.72E-10
18	T_1	$^7F_0 \rightarrow ^5D_1$	3025	0.00E+00	0.00E+00	4.20E+08	2.69E+08	1.34E+02
19	T_1	$^7F_0 \rightarrow ^5D_2$	569	1.21E+00	5.68E+03	0.00E+00	3.63E+03	2.37E+02
20	T_1	$^7F_0 \rightarrow ^5L_6$	-3273	6.82E+01	9.02E-01	0.00E+00	6.74E-06	4.42E+01
21	T_1	$^7F_0 \rightarrow ^5G_6$	-4700	6.87E+00	9.08E-02	0.00E+00	7.24E-10	4.45E+00
22	T_1	$^7F_0 \rightarrow ^5D_4$	-5534	3.55E-01	1.53E+00	0.00E+00	3.59E-12	1.21E+00
23	T_1	$^7F_1 \rightarrow ^5D_0$	5131	0.00E+00	0.00E+00	1.58E+08	5.21E+07	1.07E-03
24	T_1	$^7F_1 \rightarrow ^5D_1$	3397	5.33E-01	2.49E+03	1.19E+05	4.02E+04	3.38E-03
25	T_1	$^7F_1 \rightarrow ^5D_2$	941	0.00E+00	0.00E+00	1.05E+08	3.48E+07	3.81E+05
26	T_1	$^7F_1 \rightarrow ^5D_3$	-1931	1.18E+00	7.33E+02	0.00E+00	2.30E-02	2.42E+02
27	T_1	$^7F_1 \rightarrow ^5L_6$	-2901	1.61E+01	2.13E-01	0.00E+00	4.90E-06	5.39E+00
28	T_1	$^7F_1 \rightarrow ^5L_7$	-3933	1.86E+01	2.47E-01	0.00E+00	4.01E-08	6.23E+00
29	T_1	$^7F_1 \rightarrow ^5G_2$	-3968	0.00E+00	0.00E+00	3.73E+07	6.70E-02	1.23E+07
30	T_1	$^7F_1 \rightarrow ^5G_3$	-4198	3.78E-01	1.26E+02	0.00E+00	7.55E-08	4.18E+01
31	T_1	$^7F_1 \rightarrow ^5G_6$	-4328	3.93E+00	5.19E-02	0.00E+00	1.27E-09	1.31E+00
32	T_1	$^7F_1 \rightarrow ^5G_5$	-4339	7.82E+00	5.62E-01	0.00E+00	2.54E-09	2.77E+00
						W^T	**3.03E+08**	
						$W^{T'}$	**5.21E+07**	
						W_b^T		**1.27E+07**
						$W_b^{T'}$		**1.07E-03**

Table S11. Data obtained for ***trans*-O,O-[Eu(bisoQcd)(H_2O)$_2$]$^+$ (9')**: Δ is the energy difference between donor and acceptor states, $\boldsymbol{W_{d-d}}$, $\boldsymbol{W_{d-m}}$, and $\boldsymbol{W_{ex}}$ stands for the dipole–dipole, dipole–multipole, and exchange energy transfer mechanisms (in s^{-1}), respectively. $\boldsymbol{W}$ is the sum of them in each pathway multiplied by the thermal population of the initial acceptor level (0.64 for $^7F_0$ and 0.33 for $^7F_1$) and by the barrier factor (applied only when Δ is negative).

Pathway Label	Donor	Acceptor	Δ	W_{d-d}	W_{d-m}	W_{ex}	W	W_b
1	S_1	$^7F_0 \to {}^5D_0$	14794	1.26E-03	7.09E+00	0.00E+00	1.77E-02	2.72E-33
2	S_1	$^7F_0 \to {}^5D_1$	13060	0.00E+00	0.00E+00	8.32E+02	5.33E+02	3.34E-25
3	S_1	$^7F_0 \to {}^5D_2$	10604	1.46E+00	8.23E+03	0.00E+00	5.27E+03	4.31E-19
4	S_1	$^7F_0 \to {}^5L_6$	6762	4.71E+04	1.34E+02	0.00E+00	3.02E+04	2.49E-10
5	S_1	$^7F_0 \to {}^5G_6$	5335	4.30E+04	1.22E+02	0.00E+00	2.76E+04	2.13E-07
6	S_1	$^7F_0 \to {}^5D_4$	4501	9.23E+03	1.44E+04	0.00E+00	1.51E+04	6.36E-06
7	S_1	$^7F_1 \to {}^5D_0$	15166	0.00E+00	0.00E+00	1.21E+01	3.98E+00	1.03E-31
8	S_1	$^7F_1 \to {}^5D_1$	13432	8.10E-03	4.56E+01	1.33E-01	1.51E+01	1.59E-27
9	S_1	$^7F_1 \to {}^5D_2$	10976	0.00E+00	0.00E+00	5.24E+03	1.73E+03	2.38E-20
10	S_1	$^7F_1 \to {}^5D_3$	8104	1.10E+02	5.05E+04	0.00E+00	1.67E+04	2.20E-13
11	S_1	$^7F_1 \to {}^5L_6$	7134	6.27E+03	1.78E+01	0.00E+00	2.07E+03	2.87E-12
12	S_1	$^7F_1 \to {}^5L_7$	6102	3.57E+04	1.01E+02	0.00E+00	1.18E+04	2.30E-09
13	S_1	$^7F_1 \to {}^5G_2$	6067	0.00E+00	0.00E+00	3.66E+06	1.21E+06	2.79E-07
14	S_1	$^7F_1 \to {}^5G_3$	5837	1.21E+03	2.89E+05	0.00E+00	9.57E+04	6.65E-08
15	S_1	$^7F_1 \to {}^5G_6$	5707	1.38E+04	3.93E+01	0.00E+00	4.58E+03	5.94E-09
16	S_1	$^7F_1 \to {}^5G_5$	5696	2.81E+04	7.59E+02	0.00E+00	9.52E+03	1.30E-08
						$\boldsymbol{W^S}$	**1.43E+06**	
						$\boldsymbol{W_b^S}$		**6.94E-06**
17	T_1	$^7F_0 \to {}^5D_0$	4550	1.78E-01	1.00E+03	0.00E+00	2.51E+00	8.37E-10
18	T_1	$^7F_0 \to {}^5D_1$	2816	0.00E+00	0.00E+00	7.64E+07	4.89E+07	6.66E+01
19	T_1	$^7F_0 \to {}^5D_2$	360	2.78E-01	1.57E+03	0.00E+00	1.00E+03	1.78E+02
20	T_1	$^7F_0 \to {}^5L_6$	-3482	2.09E+01	5.92E-02	0.00E+00	7.50E-07	1.34E+01
21	T_1	$^7F_0 \to {}^5G_6$	-4909	2.01E+00	5.70E-03	0.00E+00	7.68E-11	1.29E+00
22	T_1	$^7F_0 \to {}^5D_4$	-5743	1.15E-01	1.80E-01	0.00E+00	2.06E-13	1.89E-01
23	T_1	$^7F_1 \to {}^5D_0$	4922	0.00E+00	0.00E+00	3.07E+07	1.01E+07	5.68E-04
24	T_1	$^7F_1 \to {}^5D_1$	3188	1.34E-01	7.54E+02	2.20E+04	7.51E+03	1.72E-03
25	T_1	$^7F_1 \to {}^5D_2$	732	0.00E+00	0.00E+00	1.80E+07	5.93E+06	1.77E+05
26	T_1	$^7F_1 \to {}^5D_3$	-2140	4.07E-01	1.86E+02	0.00E+00	2.15E-03	6.15E+01
27	T_1	$^7F_1 \to {}^5L_6$	-3110	5.00E+00	1.42E-02	0.00E+00	5.51E-07	1.65E+00
28	T_1	$^7F_1 \to {}^5L_7$	-4142	5.59E+00	1.59E-02	0.00E+00	4.37E-09	1.85E+00
29	T_1	$^7F_1 \to {}^5G_2$	-4177	0.00E+00	0.00E+00	5.43E+06	3.57E-03	1.79E+06
30	T_1	$^7F_1 \to {}^5G_3$	-4407	1.25E-01	2.97E+01	0.00E+00	6.53E-09	9.86E+00
31	T_1	$^7F_1 \to {}^5G_6$	-4537	1.16E+00	3.30E-03	0.00E+00	1.37E-10	3.84E-01
32	T_1	$^7F_1 \to {}^5G_5$	-4548	2.32E+00	6.26E-02	0.00E+00	2.65E-10	7.85E-01
						$\boldsymbol{W^T}$	**5.48E+07**	
						$\boldsymbol{W^{T'}}$	**1.01E+07**	
						$\boldsymbol{W_b^T}$		**1.97E+06**
						$\boldsymbol{W_b^{T'}}$		**5.68E-04**

Table S12. Data obtained for ***trans*-N,N-[Eu(bisoQcd)(H_2O)$_2$]$^+$ (10')**: Δ is the energy difference between donor and acceptor states, W_{d-d}, W_{d-m}, and W_{ex} stands for the dipole–dipole, dipole–multipole, and exchange energy transfer mechanisms (in s^{-1}), respectively. W is the sum of them in each pathway multiplied by the thermal population of the initial acceptor level (0.64 for $^7F_0$ and 0.33 for $^7F_1$) and by the barrier factor (applied only when Δ is negative).

Pathway Label	Donor	Acceptor	Δ	W_{d-d}	W_{d-m}	W_{ex}	W	W_b
1	S_1	$^7F_0 \rightarrow {}^5D_0$	14843	1.01E-03	6.78E+00	0.00E+00	1.70E-02	2.06E-33
2	S_1	$^7F_0 \rightarrow {}^5D_1$	13109	0.00E+00	0.00E+00	7.67E+02	4.91E+02	2.44E-25
3	S_1	$^7F_0 \rightarrow {}^5D_2$	10653	1.21E+00	8.12E+03	0.00E+00	5.20E+03	3.37E-19
4	S_1	$^7F_0 \rightarrow {}^5L_6$	6811	7.14E+04	1.31E+02	0.00E+00	4.58E+04	2.99E-10
5	S_1	$^7F_0 \rightarrow {}^5G_6$	5384	6.60E+04	1.21E+02	0.00E+00	4.23E+04	2.59E-07
6	S_1	$^7F_0 \rightarrow {}^5D_4$	4550	1.46E+04	1.42E+04	0.00E+00	1.84E+04	6.14E-06
7	S_1	$^7F_1 \rightarrow {}^5D_0$	15215	0.00E+00	0.00E+00	1.10E+01	3.62E+00	7.38E-32
8	S_1	$^7F_1 \rightarrow {}^5D_1$	13481	6.59E-03	4.41E+01	1.23E-01	1.46E+01	1.22E-27
9	S_1	$^7F_1 \rightarrow {}^5D_2$	11025	0.00E+00	0.00E+00	4.91E+03	1.62E+03	1.77E-20
10	S_1	$^7F_1 \rightarrow {}^5D_3$	8153	1.64E+02	5.07E+04	0.00E+00	1.68E+04	1.76E-13
11	S_1	$^7F_1 \rightarrow {}^5L_6$	7183	9.48E+03	1.73E+01	0.00E+00	3.14E+03	3.43E-12
12	S_1	$^7F_1 \rightarrow {}^5L_7$	6151	5.44E+04	9.96E+01	0.00E+00	1.80E+04	2.78E-09
13	S_1	$^7F_1 \rightarrow {}^5G_2$	6116	0.00E+00	0.00E+00	3.56E+06	1.17E+06	2.15E-07
14	S_1	$^7F_1 \rightarrow {}^5G_3$	5886	1.86E+03	2.95E+05	0.00E+00	9.80E+04	5.40E-08
15	S_1	$^7F_1 \rightarrow {}^5G_6$	5756	2.12E+04	3.87E+01	0.00E+00	7.00E+03	7.19E-09
16	S_1	$^7F_1 \rightarrow {}^5G_5$	5745	4.30E+04	7.44E+02	0.00E+00	1.44E+04	1.56E-08
						W^S	1.45E+06	
						W_b^S		6.70E-06
17	T_1	$^7F_0 \rightarrow {}^5D_0$	4524	1.63E-01	1.09E+03	0.00E+00	2.73E+00	1.03E-09
18	T_1	$^7F_0 \rightarrow {}^5D_1$	2790	0.00E+00	0.00E+00	7.86E+07	5.03E+07	7.77E+01
19	T_1	$^7F_0 \rightarrow {}^5D_2$	334	2.50E-01	1.68E+03	0.00E+00	1.07E+03	2.16E+02
20	T_1	$^7F_0 \rightarrow {}^5L_6$	-3508	3.29E+01	6.01E-02	0.00E+00	1.04E-06	2.11E+01
21	T_1	$^7F_0 \rightarrow {}^5G_6$	-4935	3.14E+00	5.75E-03	0.00E+00	1.06E-10	2.01E+00
22	T_1	$^7F_0 \rightarrow {}^5D_4$	-5769	1.84E-01	1.79E-01	0.00E+00	2.24E-13	2.33E-01
23	T_1	$^7F_1 \rightarrow {}^5D_0$	4896	0.00E+00	0.00E+00	3.19E+07	1.05E+07	6.68E-04
24	T_1	$^7F_1 \rightarrow {}^5D_1$	3162	1.22E-01	8.16E+02	2.27E+04	7.75E+03	2.01E-03
25	T_1	$^7F_1 \rightarrow {}^5D_2$	706	0.00E+00	0.00E+00	1.83E+07	6.04E+06	2.05E+05
26	T_1	$^7F_1 \rightarrow {}^5D_3$	-2166	6.36E-01	1.97E+02	0.00E+00	2.01E-03	6.52E+01
27	T_1	$^7F_1 \rightarrow {}^5L_6$	-3136	7.88E+00	1.44E-02	0.00E+00	7.65E-07	2.61E+00
28	T_1	$^7F_1 \rightarrow {}^5L_7$	-4168	8.77E+00	1.60E-02	0.00E+00	6.04E-09	2.90E+00
29	T_1	$^7F_1 \rightarrow {}^5G_2$	-4203	0.00E+00	0.00E+00	5.42E+06	3.15E-03	1.79E+06
30	T_1	$^7F_1 \rightarrow {}^5G_3$	-4433	1.96E-01	3.12E+01	0.00E+00	6.06E-09	1.04E+01
31	T_1	$^7F_1 \rightarrow {}^5G_6$	-4563	1.82E+00	3.33E-03	0.00E+00	1.89E-10	6.02E-01
32	T_1	$^7F_1 \rightarrow {}^5G_5$	-4574	3.63E+00	6.29E-02	0.00E+00	3.62E-10	1.22E+00
						W^T	5.63E+07	
						$W^{T'}$	1.05E+07	
						W_b^T		1.99E+06
						$W_b^{T'}$		6.68E-04

Table S13. Data obtained for ***trans*-O,O-[Eu(isoQC3A) $(H_2O)_2$] (11')**: Δ is the energy difference between donor and acceptor states, $\boldsymbol{W_{d-d}}$, $\boldsymbol{W_{d-m}}$, and $\boldsymbol{W_{ex}}$ stands for the dipole–dipole, dipole–multipole, and exchange energy transfer mechanisms (in s^{-1}), respectively. $\boldsymbol{W}$ is the sum of them in each pathway multiplied by the thermal population of the initial acceptor level (0.64 for $^7F_0$ and 0.33 for $^7F_1$) and by the barrier factor (applied only when Δ is negative).

Pathway Label	Donor	Acceptor	Δ	W_{d-d}	W_{d-m}	W_{ex}	W	W_b
1	S_1	$^7F_0 \rightarrow ^5D_0$	14707	9.03E-03	8.07E+00	0.00E+00	2.02E-02	4.71E-33
2	S_1	$^7F_0 \rightarrow ^5D_1$	12973	0.00E+00	0.00E+00	9.17E+02	5.87E+02	5.60E-25
3	S_1	$^7F_0 \rightarrow ^5D_2$	10517	9.91E+00	8.85E+03	0.00E+00	5.67E+03	7.06E-19
4	S_1	$^7F_0 \rightarrow ^5L_6$	6675	6.27E+04	1.30E+02	0.00E+00	4.02E+04	5.03E-10
5	S_1	$^7F_0 \rightarrow ^5G_6$	5248	5.62E+04	1.17E+02	0.00E+00	3.60E+04	4.23E-07
6	S_1	$^7F_0 \rightarrow ^5D_4$	4414	1.27E+04	1.40E+04	0.00E+00	1.71E+04	1.09E-05
7	S_1	$^7F_1 \rightarrow ^5D_0$	15079	0.00E+00	0.00E+00	1.37E+01	4.51E+00	1.77E-31
8	S_1	$^7F_1 \rightarrow ^5D_1$	13345	5.71E-02	5.10E+01	1.48E-01	1.69E+01	2.71E-27
9	S_1	$^7F_1 \rightarrow ^5D_2$	10889	0.00E+00	0.00E+00	5.62E+03	1.85E+03	3.87E-20
10	S_1	$^7F_1 \rightarrow ^5D_3$	8017	2.05E+02	5.25E+04	0.00E+00	1.74E+04	3.48E-13
11	S_1	$^7F_1 \rightarrow ^5L_6$	7047	8.39E+03	1.75E+01	0.00E+00	2.77E+03	5.82E-12
12	S_1	$^7F_1 \rightarrow ^5L_7$	6015	4.71E+04	9.80E+01	0.00E+00	1.56E+04	4.62E-09
13	S_1	$^7F_1 \rightarrow ^5G_2$	5980	0.00E+00	0.00E+00	3.67E+06	1.21E+06	4.25E-07
14	S_1	$^7F_1 \rightarrow ^5G_3$	5750	1.95E+03	2.91E+05	0.00E+00	9.68E+04	1.02E-07
15	S_1	$^7F_1 \rightarrow ^5G_6$	5620	1.82E+04	3.78E+01	0.00E+00	6.01E+03	1.18E-08
16	S_1	$^7F_1 \rightarrow ^5G_5$	5609	3.69E+04	7.50E+02	0.00E+00	1.24E+04	2.58E-08
						$\boldsymbol{W^S}$	**1.46E+06**	
						$\boldsymbol{W_b^S}$		**1.19E-05**
17	T_1	$^7F_0 \rightarrow ^5D_0$	4702	9.41E-01	8.41E+02	0.00E+00	2.11E+00	3.39E-10
18	T_1	$^7F_0 \rightarrow ^5D_1$	2968	0.00E+00	0.00E+00	6.61E+07	4.23E+07	2.78E+01
19	T_1	$^7F_0 \rightarrow ^5D_2$	512	1.62E+00	1.45E+03	0.00E+00	9.28E+02	7.96E+01
20	T_1	$^7F_0 \rightarrow ^5L_6$	-3330	2.75E+01	5.72E-02	0.00E+00	2.04E-06	1.76E+01
21	T_1	$^7F_0 \rightarrow ^5G_6$	-4757	2.73E+00	5.69E-03	0.00E+00	2.17E-10	1.75E+00
22	T_1	$^7F_0 \rightarrow ^5D_4$	-5591	1.71E-01	1.88E-01	0.00E+00	5.21E-13	2.30E-01
23	T_1	$^7F_1 \rightarrow ^5D_0$	5074	0.00E+00	0.00E+00	2.53E+07	8.35E+06	2.26E-04
24	T_1	$^7F_1 \rightarrow ^5D_1$	3340	7.29E-01	6.52E+02	1.89E+04	6.44E+03	7.12E-04
25	T_1	$^7F_1 \rightarrow ^5D_2$	884	0.00E+00	0.00E+00	1.63E+07	5.38E+06	7.76E+04
26	T_1	$^7F_1 \rightarrow ^5D_3$	-1988	7.13E-01	1.82E+02	0.00E+00	4.36E-03	6.04E+01
27	T_1	$^7F_1 \rightarrow ^5L_6$	-2958	6.53E+00	1.36E-02	0.00E+00	1.49E-06	2.16E+00
28	T_1	$^7F_1 \rightarrow ^5L_7$	-3990	7.48E+00	1.56E-02	0.00E+00	1.21E-08	2.47E+00
29	T_1	$^7F_1 \rightarrow ^5G_2$	-4025	0.00E+00	0.00E+00	5.52E+06	7.54E-03	1.82E+06
30	T_1	$^7F_1 \rightarrow ^5G_3$	-4255	2.06E-01	3.07E+01	0.00E+00	1.40E-08	1.02E+01
31	T_1	$^7F_1 \rightarrow ^5G_6$	-4385	1.57E+00	3.26E-03	0.00E+00	3.82E-10	5.19E-01
32	T_1	$^7F_1 \rightarrow ^5G_5$	-4396	3.13E+00	6.36E-02	0.00E+00	7.36E-10	1.06E+00
						$\boldsymbol{W^T}$	**4.77E+07**	
						$\boldsymbol{W^{T'}}$	**8.35E+06**	
						$\boldsymbol{W_b^T}$		**1.90E+06**
						$\boldsymbol{W_b^{T'}}$		**2.26E-04**

Table S14. Data obtained for ***trans*-N,O-[Eu(isoQC3A) (H_2O)$_2$] (12')**: Δ is the energy difference between donor and acceptor states, $\boldsymbol{W_{d-d}}$, $\boldsymbol{W_{d-m}}$, and $\boldsymbol{W_{ex}}$ stands for the dipole–dipole, dipole–multipole, and exchange energy transfer mechanisms (in s^{-1}), respectively. $\boldsymbol{W}$ is the sum of them in each pathway multiplied by the thermal population of the initial acceptor level (0.64 for $^7F_0$ and 0.33 for $^7F_1$) and by the barrier factor (applied only when Δ is negative).

**Pathway Label**	**Donor**	**Acceptor**	**Δ**	$\boldsymbol{W_{d-d}}$	$\boldsymbol{W_{d-m}}$	$\boldsymbol{W_{ex}}$	$\boldsymbol{W}$	$\boldsymbol{W_b}$
1	S_1	$^7F_0 \rightarrow {}^5D_0$	14942	2.65E-03	4.59E+00	0.00E+00	1.15E-02	8.67E-34
2	S_1	$^7F_0 \rightarrow {}^5D_1$	13208	0.00E+00	0.00E+00	5.94E+02	3.80E+02	1.17E-25
3	S_1	$^7F_0 \rightarrow {}^5D_2$	10752	3.39E+00	5.86E+03	0.00E+00	3.75E+03	1.51E-19
4	S_1	$^7F_0 \rightarrow {}^5L_6$	6910	4.38E+04	1.08E+02	0.00E+00	2.81E+04	1.14E-10
5	S_1	$^7F_0 \rightarrow {}^5G_6$	5483	4.14E+04	1.02E+02	0.00E+00	2.65E+04	1.01E-07
6	S_1	$^7F_0 \rightarrow {}^5D_4$	4649	9.94E+03	1.24E+04	0.00E+00	1.43E+04	2.96E-06
7	S_1	$^7F_1 \rightarrow {}^5D_0$	15314	0.00E+00	0.00E+00	8.21E+00	2.71E+00	3.43E-32
8	S_1	$^7F_1 \rightarrow {}^5D_1$	13580	1.76E-02	3.05E+01	9.44E-02	1.01E+01	5.23E-28
9	S_1	$^7F_1 \rightarrow {}^5D_2$	11124	0.00E+00	0.00E+00	3.93E+03	1.30E+03	8.76E-21
10	S_1	$^7F_1 \rightarrow {}^5D_3$	8252	1.23E+02	3.81E+04	0.00E+00	1.26E+04	8.17E-14
11	S_1	$^7F_1 \rightarrow {}^5L_6$	7282	5.79E+03	1.42E+01	0.00E+00	1.91E+03	1.30E-12
12	S_1	$^7F_1 \rightarrow {}^5L_7$	6250	3.37E+04	8.29E+01	0.00E+00	1.12E+04	1.07E-09
13	S_1	$^7F_1 \rightarrow {}^5G_2$	6215	0.00E+00	0.00E+00	3.07E+06	1.01E+06	1.15E-07
14	S_1	$^7F_1 \rightarrow {}^5G_3$	5985	1.34E+03	2.29E+05	0.00E+00	7.61E+04	2.60E-08
15	S_1	$^7F_1 \rightarrow {}^5G_6$	5855	1.32E+04	3.24E+01	0.00E+00	4.37E+03	2.78E-09
16	S_1	$^7F_1 \rightarrow {}^5G_5$	5844	2.68E+04	6.36E+02	0.00E+00	9.07E+03	6.10E-09
						$\boldsymbol{W^S}$	**1.20E+06**	
						$\boldsymbol{W_b^S}$		**3.21E-06**
17	T_1	$^7F_0 \rightarrow {}^5D_0$	4570	5.20E-01	9.00E+02	0.00E+00	2.25E+00	6.83E-10
18	T_1	$^7F_0 \rightarrow {}^5D_1$	2836	0.00E+00	0.00E+00	7.29E+07	4.67E+07	5.79E+01
19	T_1	$^7F_0 \rightarrow {}^5D_2$	380	8.21E-01	1.42E+03	0.00E+00	9.11E+02	1.47E+02
20	T_1	$^7F_0 \rightarrow {}^5L_6$	-3462	2.30E+01	5.64E-02	0.00E+00	9.05E-07	1.47E+01
21	T_1	$^7F_0 \rightarrow {}^5G_6$	-4889	2.22E+00	5.45E-03	0.00E+00	9.32E-11	1.42E+00
22	T_1	$^7F_0 \rightarrow {}^5D_4$	-5723	1.41E-01	1.75E-01	0.00E+00	2.42E-13	2.02E-01
23	T_1	$^7F_1 \rightarrow {}^5D_0$	4942	0.00E+00	0.00E+00	2.92E+07	9.62E+06	4.90E-04
24	T_1	$^7F_1 \rightarrow {}^5D_1$	3208	3.92E-01	6.78E+02	2.10E+04	7.15E+03	1.49E-03
25	T_1	$^7F_1 \rightarrow {}^5D_2$	752	0.00E+00	0.00E+00	1.73E+07	5.70E+06	1.55E+05
26	T_1	$^7F_1 \rightarrow {}^5D_3$	-2120	5.48E-01	1.70E+02	0.00E+00	2.16E-03	5.63E+01
27	T_1	$^7F_1 \rightarrow {}^5L_6$	-3090	5.49E+00	1.35E-02	0.00E+00	6.65E-07	1.82E+00
28	T_1	$^7F_1 \rightarrow {}^5L_7$	-4122	6.16E+00	1.51E-02	0.00E+00	5.28E-09	2.04E+00
29	T_1	$^7F_1 \rightarrow {}^5G_2$	-4157	0.00E+00	0.00E+00	5.29E+06	3.83E-03	1.75E+06
30	T_1	$^7F_1 \rightarrow {}^5G_3$	-4387	1.60E-01	2.74E+01	0.00E+00	6.62E-09	9.09E+00
31	T_1	$^7F_1 \rightarrow {}^5G_6$	-4517	1.28E+00	3.15E-03	0.00E+00	1.65E-10	4.24E-01
32	T_1	$^7F_1 \rightarrow {}^5G_5$	-4528	2.56E+00	6.06E-02	0.00E+00	3.20E-10	8.65E-01
						$\boldsymbol{W^T}$	**5.24E+07**	
						$\boldsymbol{W^{T'}}$	**9.62E+06**	
						$\boldsymbol{W_b^T}$		**1.90E+06**
						$\boldsymbol{W_b^{T'}}$		**4.90E-04**

S5. ISC rates and decay lifetimes of the S_1 and T_1

According to the Marcus–Levich framework [20–22], the W_{ISC} can be evaluated as [23]:

$$W_{ISC} = \frac{2\pi}{\hbar}\langle S_1|\hat{H}_{SO}|T_1\rangle^2 \frac{1}{\sqrt{4\pi\lambda_M k_B T}} e^{-\left[\frac{(\Delta E_{ST}+\lambda_M)^2}{4\lambda_M k_B T}\right]} \quad \text{(S10)}$$

where $\langle S_1|\hat{H}_{SO}|T_1\rangle$ is the spin-orbital coupling matrix element (SOCME), λ_M is the so-called Markus reorganization energy, ΔE_{ST} is the energy difference between S_1 and T_1 states, k_B is the constant of Boltzmann, $\hbar$ is the reduced Planck's constant, and T is the temperature (T=300 K was considered in our calculations). λ_M is calculated as an average of the reorganization energies as follows [24]:

$$\lambda_M = \frac{[E(4) - E(1)] + [E(3) - E(2)]}{2} \quad \text{(S11)}$$

where $E(X)$ are energies in different points as shown in Figure S14.

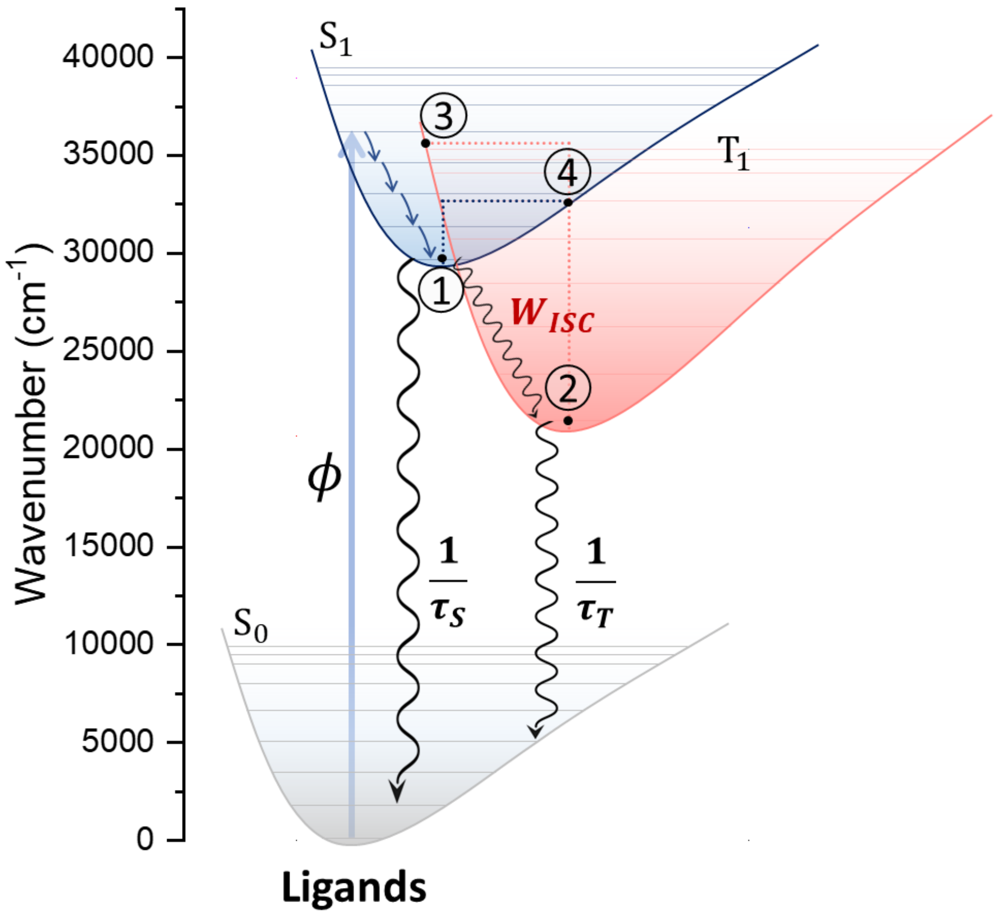

Figure S14. Sketch of the potential energy surfaces for the ground (S_0) and excited (S_1 and T_1) states.

Table S15. Values of $\langle S_1|\hat{H}_{SO}|T_1\rangle$ (in cm^{-1}), reorganization energy λ_M (in cm^{-1}), and $S_1 \rightarrow T_1$ intersystem crossing rate W_{ISC} (in s^{-1}) for some studied compounds.

Group	Isomer	$\langle S_1\|\hat{H}_{SO}\|T_1\rangle$	λ_M	W_{ISC}
I	**1'**	2.38	4016	3.94×10^{7}
	**2'**	-	-	-
	**3'**	-	-	-
	**4'**	4.39	-	-
II	**5'**	1.77	3051	1.50×10^{7}
	**6'**	-	-	-
	**7'**	-	-	-
	**8'**	4.11	-	-
III	**9'**	4.44	-	-
	**10'**	2.41	-	-
	**11'**	2.34	3043	6.72×10^{4}
	**12'**	9.16	-	-

Due to the tough task in the convergence of the SOC-TD-DFT calculations, especially the relaxations of the T_1 state (energy in point 4 in Figure S14), it was not possible to obtain the λ_M for all complexes. However, a value of W_{ISC} for one complex of each group was obtained, thus, we consider the W_{ISC} for isomers **1'**, **5'**, and **11'** as representative of their respective groups.

Considering the SOC calculations to estimate the ISC rates, the S_1 and T_1 lifetimes (τ_S and τ_T) were estimated from the dipole strengths S of the vertical (non-relaxed) $S_0 \rightarrow S_1$ and $S_0 \rightarrow T_1$ transitions [18,25]:

$$\tau = \frac{3}{4}\frac{\hbar c^3}{(2\pi c\sigma)^3 S} \tag{S12}$$

where c is the speed of light and σ is the energy of the transition. Table S16 shows the values of S (in esu^2·cm^2) and the values of τ_S and τ_T (in s).

Table S16. Values of dipole strength (in esu^2·cm^2) of $S_0 \rightarrow S_1$ (S_S) and $S_0 \rightarrow T_1$ (S_T) transitions and the corresponding lifetimes (τ_S and τ_T, in s) for the studied compounds.

		S_1		T_1	
Group	Isomer	S_S	τ_S	S_T	τ_T
I	**1'**	4.60×10^{-37}	1.13×10^{-7}	5.78×10^{-40}	1.78×10^{-4}
	**2'**	-	-	-	-
	**3'**	-	-	-	-
	**4'**	3.01×10^{-37}	1.68×10^{-7}	8.54×10^{-42}	1.20×10^{-2}
II	**5'**	3.45×10^{-37}	2.71×10^{-7}	1.38×10^{-40}	2.11×10^{-3}
	**6'**	-	-	-	-
	**7'**	-	-	-	-
	**8'**	1.45×10^{-37}	6.83×10^{-7}	2.33×10^{-39}	1.28×10^{-4}
III	**9'**	8.25×10^{-37}	1.17×10^{-7}	9.76×10^{-40}	3.14×10^{-4}
	**10'**	4.42×10^{-37}	2.17×10^{-7}	2.32×10^{-39}	1.32×10^{-4}
	**11'**	1.71×10^{-37}	5.67×10^{-7}	1.81×10^{-40}	1.65×10^{-3}
	**12'**	5.59×10^{-37}	1.70×10^{-7}	1.11×10^{-39}	2.75×10^{-4}

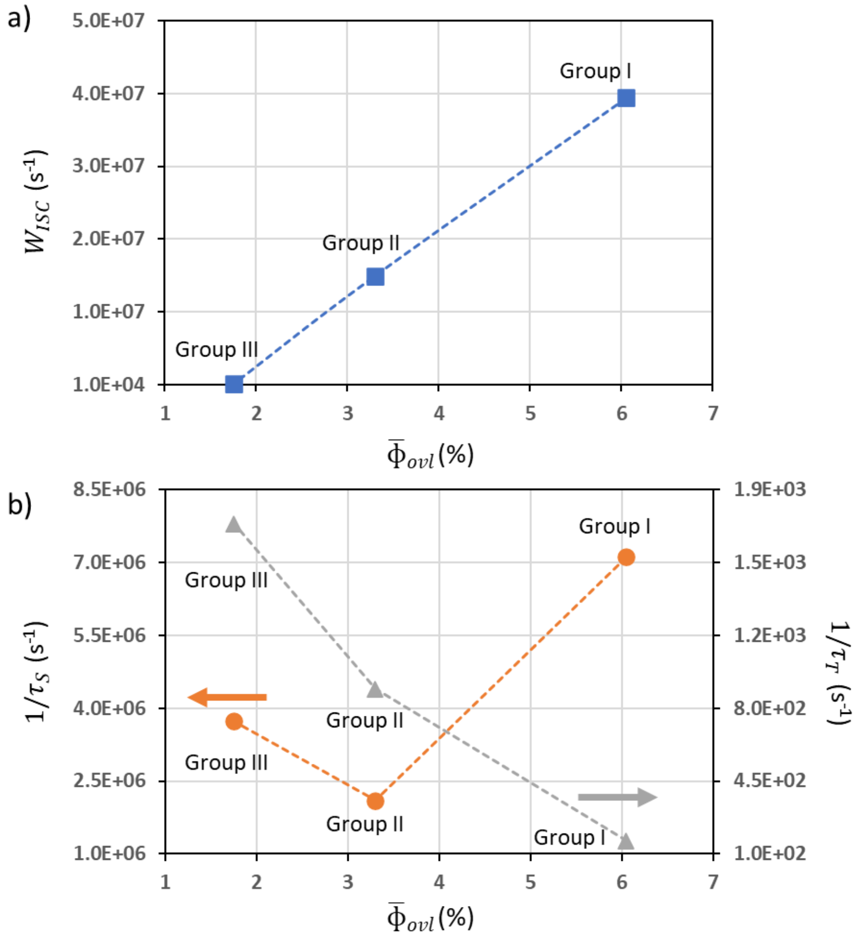

Figure S15. Trends between the average of experimental overall quantum yields ($\overline{\Phi}_{ovl}$) and computationally obtained W_{ISC} (a), $1/\tau_S$ and $1/\tau_T$ (b) for the group of Eu(III) complexes.

S6. Experimental quantum yields

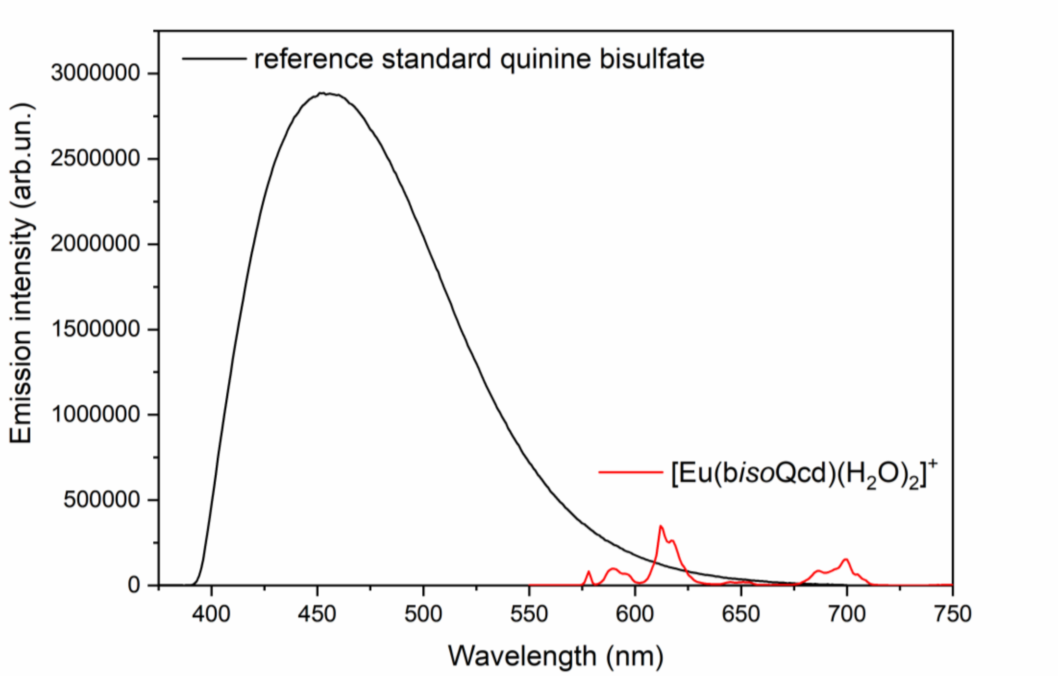

Figure S16. Overlap of the emission spectra of the reference standard and $[Eu(b\textit{iso}Qcd)(H_2O)_2]^+$ complex for the Quantum Yield measurement. Slits width was adjusted as 3/3 nm for excitation and 1.5/1.5 nm for emission. $A_s = 0.06$; $A_u = 0.064$. λ_{exc} = 347 nm and 328 nm, for the standard and the complex, respectively.

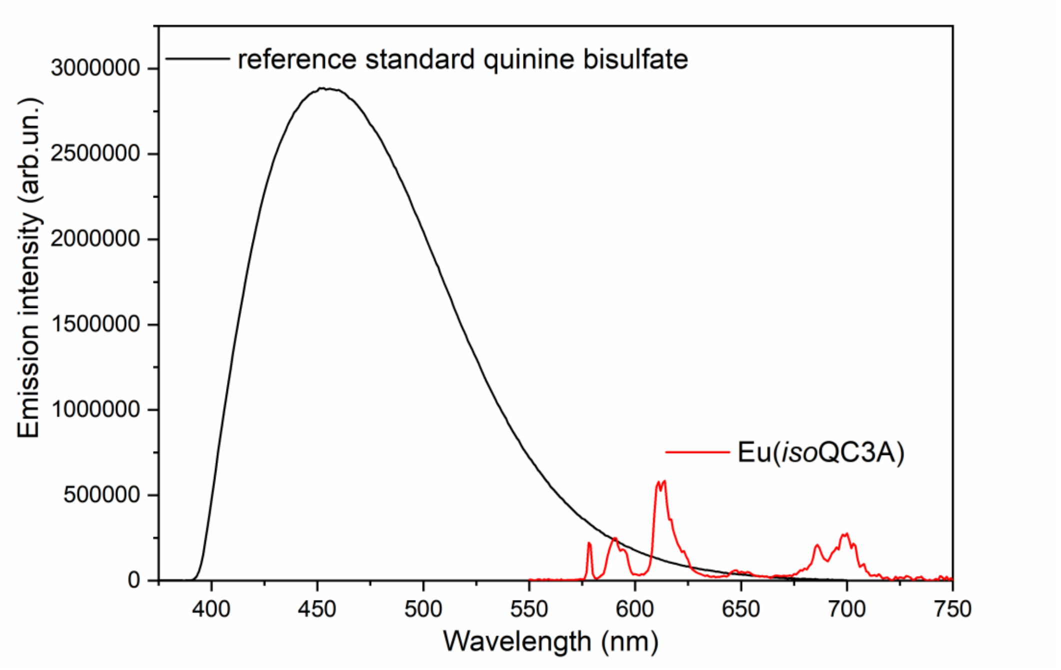

Figure S17. Overlap of the emission spectra of the reference standard and $Eu(\textit{iso}QC3A)(H_2O)_2$ complex for the Quantum Yield measurement. Slits width was adjusted as 3/3 nm for excitation and 1.5/1.5 nm for emission. $A_s = 0.06$; $A_u = 0.076$. λ_{exc} = 347 nm and 328 nm, for the standard and the complex, respectively.

References

(1) Judd, B. R. Optical Absorption Intensities of Rare-Earth Ions. *Phys. Rev.* **1962**, *127* (3), 750–761. https://doi.org/10.1103/PhysRev.127.750.

(2) Jørgensen, C. K.; Judd, B. R. Hypersensitive Pseudoquadrupole Transitions in Lanthanides. *Mol. Phys.* **1964**, *8* (3), 281–290. https://doi.org/10.1080/00268976400100321.

(3) Ofelt, G. S. Intensities of Crystal Spectra of Rare-Earth Ions. *J. Chem. Phys.* **1962**, *37* (3), 511–520. https://doi.org/10.1063/1.1701366.

(4) Wybourne, B. G. *Spectroscopic Properties of Rare Earths*; John Wiley & Sons: New York, 1965.

(5) de Sá, G. F.; Malta, O. L.; de Mello Donegá, C.; Simas, A. M.; Longo, R. L.; Santa-Cruz, P. A.; da Silva, E. F. Spectroscopic Properties and Design of Highly Luminescent Lanthanide Coordination Complexes. *Coord. Chem. Rev.* **2000**, *196* (1), 165–195. https://doi.org/10.1016/S0010-8545(99)00054-5.

(6) Moura Jr., R. T.; Carneiro Neto, A. N.; Longo, R. L.; Malta, O. L. On the Calculation and Interpretation of Covalency in the Intensity Parameters of 4f–4f Transitions in Eu3+ Complexes Based on the Chemical Bond Overlap Polarizability. *J. Lumin.* **2016**, *170,* 420–430. https://doi.org/10.1016/j.jlumin.2015.08.016.

(7) Kariaka, N.; Trush, V. A.; Dyakonenko, V. V.; Shishkina, S. V.; Smola, S. S.; Rusakova, N. V.; Sliva, T. Y.; Gawryszewska, P.; Neto, A. N. C.; Malta, O. L.; Amirkhanov, V. M. New Luminescent Lanthanide Tetrakis-complexes $NEt_4[LnL_4]$ Based on Dimethyl-N-benzoylamidophosphate. *ChemPhysChem* **2022**. https://doi.org/10.1002/cphc.202200129.

(8) Carneiro Neto, A. N.; Moura, R. T.; Aguiar, E. C.; Santos, C. V; de Medeiros, M. A. F. L. B. Theoretical Study of Geometric and Spectroscopic Properties of Eu(III) Complexes with Ruhemann's Purple Ligands. *J. Lumin.* **2018**, *201* (83), 451–459. https://doi.org/10.1016/j.jlumin.2018.05.014.

(9) Malta, O. L.; Batista, H. J.; Carlos, L. D. Overlap Polarizability of a Chemical Bond: A Scale of Covalency and Application to Lanthanide Compounds. *Chem. Phys.* **2002**, *282* (1), 21–30. https://doi.org/10.1016/S0301-0104(02)00631-6.

(10) Moura, R. T.; Malta, O. L.; Longo, R. L. The Chemical Bond Overlap Plasmon as a Tool for Quantifying Covalency in Solid State Materials and Its Applications to Spectroscopy. *Int. J. Quantum Chem.* **2011**, *111* (7–8), 1626–1638. https://doi.org/10.1002/qua.22782.

(11) Moura Jr., R. T.; Duarte, G. C. S.; da Silva, T. E.; Malta, O. L.; Longo, R. L. Features of Chemical Bonds Based on the Overlap Polarizabilities: Diatomic and Solid-State Systems with the Frozen-Density Embedding Approach. *Phys. Chem. Chem. Phys.* **2015**, *17* (12), 7731–7742. https://doi.org/10.1039/C4CP05283H.

(12) Ramalho, J. F. C. B.; Carneiro Neto, A. N.; Carlos, L. D.; André, P. S.; Ferreira, R. A. S. Lanthanides

for the New Generation of Optical Sensing and Internet of Things. In *Handbook on the Physics and Chemistry of Rare Earths*; Bünzli, J.-C. G., Pecharsky, V. K., Eds.; Elsevier B.V, 2022. https://doi.org/10.1016/bs.hpcre.2021.12.001.

(13) Hecht, E. *Optics*, fifth edit.; Pearson Education Limited, 2015.

(14) Carnall, W. T.; Crosswhite, H.; Crosswhite, H. M. *Energy Level Structure and Transition Probabilities in the Spectra of the Trivalent Lanthanides in LaF_3*; Argonne, IL, United States, 1978. https://doi.org/10.2172/6417825.

(15) Ofelt, G. S. Structure of the f^6 Configuration with Application to Rare-Earth Ions. *J. Chem. Phys.* **1963**, *38* (9), 2171–2180. https://doi.org/10.1063/1.1733947.

(16) Blois, L.; Carneiro Neto, A. N.; Longo, R. L.; Costa, I. F.; Paolini, T. B.; Brito, H. F.; Malta, O. L. Об Экспериментальном Определении Параметров Интенсивности 4f-4f-Переходов По Спектрам Излучения Соединений Европия (III). *Оптика и спектроскопия* **2022**, *130* (1), 207. https://doi.org/10.21883/OS.2022.01.51909.35-21.

(17) Blois, L.; Neto, A. N. C.; Longo, R. L.; Costa, I. F.; Paolini, T. B.; Brito, H. F.; Malta, O. L. On the Experimental Determination of 4f–4f Intensity Parameters from the Emission Spectra of Europium (III) Compounds. *Opt. Spectrosc.* **2022**, *130* (1), 10–17. https://doi.org/10.1134/S0030400X2201009X.

(18) Carneiro Neto, A. N.; Teotonio, E. E. S.; de Sá, G. F.; Brito, H. F.; Legendziewicz, J.; Carlos, L. D.; Felinto, M. C. F. C.; Gawryszewska, P.; Moura Jr., R. T.; Longo, R. L.; Faustino, W. M.; Malta, O. L. Modeling Intramolecular Energy Transfer in Lanthanide Chelates: A Critical Review and Recent Advances. In *Handbook on the Physics and Chemistry of Rare Earths, volume 56*; Bünzli, J.-C. G., Pecharsky, V. K., Eds.; Elsevier, 2019; pp 55–162. https://doi.org/10.1016/bs.hpcre.2019.08.001.

(19) Moura Jr., R. T.; Carneiro Neto, A. N.; Aguiar, E. C.; Santos-Jr., C. V.; de Lima, E. M.; Faustino, W. M.; Teotonio, E. E. S.; Brito, H. F.; Felinto, M. C. F. C.; Ferreira, R. A. S.; Carlos, L. D.; Longo, R. L.; Malta, O. L. JOYSpectra: A Web Platform for Luminescence of Lanthanides. *Opt. Mater. X* **2021**, *11*, 100080. https://doi.org/10.1016/j.omx.2021.100080.

(20) Marcus, R. A. On the Theory of Oxidation-Reduction Reactions Involving Electron Transfer. I. *J. Chem. Phys.* **1956**, *24* (5), 966–978. https://doi.org/10.1063/1.1742723.

(21) Marcus, R. A. Electron Transfer Reactions in Chemistry: Theory and Experiment (Nobel Lecture). *Angew. Chemie Int. Ed. English* **1993**, *32* (8), 1111–1121. https://doi.org/10.1002/anie.199311113.

(22) Bixon, M.; Jortner, J. Intramolecular Radiationless Transitions. *J. Chem. Phys.* **1968**, *48* (2), 715–726. https://doi.org/10.1063/1.1668703.

(23) Wang, L.; Ou, Q.; Peng, Q.; Shuai, Z. Theoretical Characterizations of TADF Materials: Roles of ΔG and the Singlet–Triplet Excited States Interconversion. *J. Phys. Chem. A* **2021**, *125* (7), 1468–1475. https://doi.org/10.1021/acs.jpca.0c09767.

(24) Brédas, J. L.; Beljonne, D.; Coropceanu, V.; Cornil, J. Charge-Transfer and Energy-Transfer Processes

in π-Conjugated Oligomers and Polymers: A Molecular Picture. *Chem. Rev.* **2004**, *104* (11), 4971–5003. https://doi.org/10.1021/cr040084k.

(25) Hebestreit, M.-L.; Schneider, M.; Lartian, H.; Betz, V.; Heinrich, M.; Lindic, M.; Choi, M. Y.; Schmitt, M. Structures, Dipole Moments and Excited State Lifetime of Isolated 4-Cyanoindole in Its Ground and Lowest Electronically Excited Singlet States. *Phys. Chem. Chem. Phys.* **2019**, *21* (27), 14766–14774. https://doi.org/10.1039/C9CP01618J.
